# Supplementary material for: Rhodium(III)-Catalyzed [4+2] Annulation via C-H Activation: Synthesis of Multi-Substituted Naphthalenone Sulfoxonium Ylides
Source: Molecules. 2019 May 16;24(10):1884. doi: 10.3390/molecules24101884 (PMC6572249; doi:10.3390/molecules24101884)
Supplement: Supplementary file 1 [file molecules-24-01884-s001.pdf]

Supporting Information

Rhodium(III)-Catalyzed Synthesis of Naphthalenone Sulfoxonium Ylides via C-H Activation and Cascade [4+2] Annulation

Xiaohan Song [1,2], Xu Han [1,2], Rui Zhang,[1,2], Hong Liu [1,2,*] and Jiang Wang [1,2,*]

[1]State Key Laboratory of Drug Research and CAS Key Laboratory of Receptor Research, Shanghai Institute of Materia Medica, Chinese Academy of Sciences, Shanghai, Shanghai 201203, China.

[2]University of Chinese Academy of Sciences, No.19A Yuquan Road, Beijing 100049, China.

*Correspondence: jwang@simm.ac.cn and hliu@simm.ac.cn.

Contents

(A) Mechanistic Studies ········ **S3**

(B) X-ray Crystallographic Data ········ **S5**

(C) Copies of ^{1}H NMR and ^{13}C NMR Spectra for the Products ········ **S6**

(D) Copies of ^{19}F NMR Spectra for the Products ········ **S37**

(E) Copies of Mass Spectra for the Products ········ **S40**

(A) Mechanistic Studies

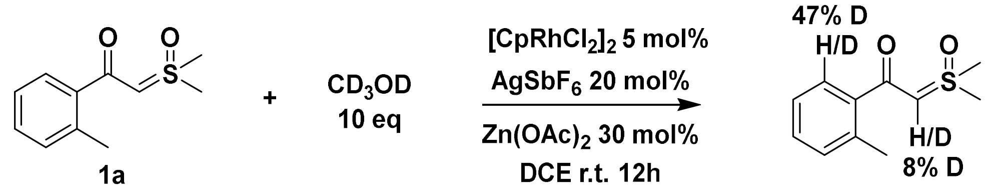

A tube was charged with $[Cp^*RhCl_2]_2$ (6.0 mg, 5 mol%), $AgSbF_6$ (14 mg, 20 mol%), $Zn(OAc)_2$ (14 mg, 30 mol%), sulfoxonium ylide (**1a**, 0.2 mmol), CD_3OD (72 mg, 10 eq) and DCE (3 mL). The reaction mixture was stirred at r.t. for 12 h under air condition. After that, the solvent was removed under reduced pressure and the residue was purified by silica gel chromatography using DCM/MeOH (96:4) to afford the product, which was characterized by ^1H NMR spectroscopy. ^1H NMR analysis of **1a** revealed 47% deuteration at the 6-position of phenyl ring and 8% deuteration at the α-position of the carbonyl.

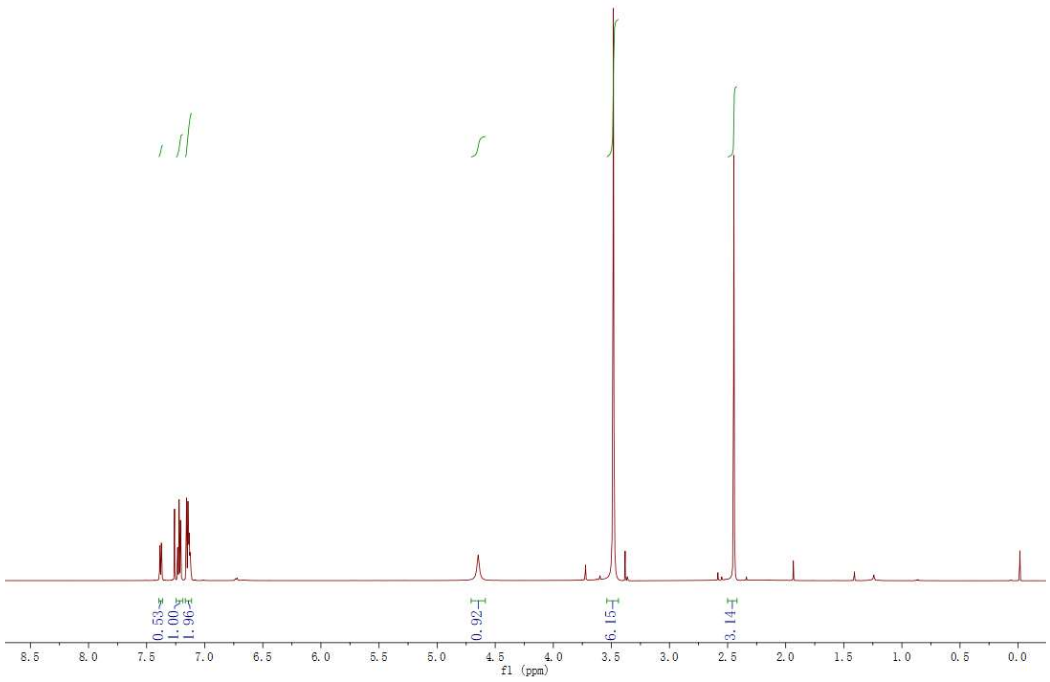

1a + 2k —standard conditions→ 3ak

1a-d_7 + 2k —standard conditions→ 3ak-d_6

KIE = 2.8

Two tubes were charged with $[Cp^*RhCl_2]_2$ (6.0 mg, 5 mol %), $AgSbF_6$ (14 mg, 20 mol%), $Zn(OAc)_2$ (14 mg, 30 mol%), sulfoxonium ylide (**1a** or **1a-*d*$_7$** , 0.2 mmol), α-diazocarbonyl compounds (**2g**, 0.24 mmol) and DCE (3 mL). The reaction mixture was stirred at r.t. for 2 h under air condition. After that, the solvent was removed under reduced pressure and the residue was purified by silica gel chromatography using DCM/MeOH (99:1) to afford the product. The KIE value was determined to be k_H/k_D = 2.8 on the basis of ^{1}H NMR analysis.

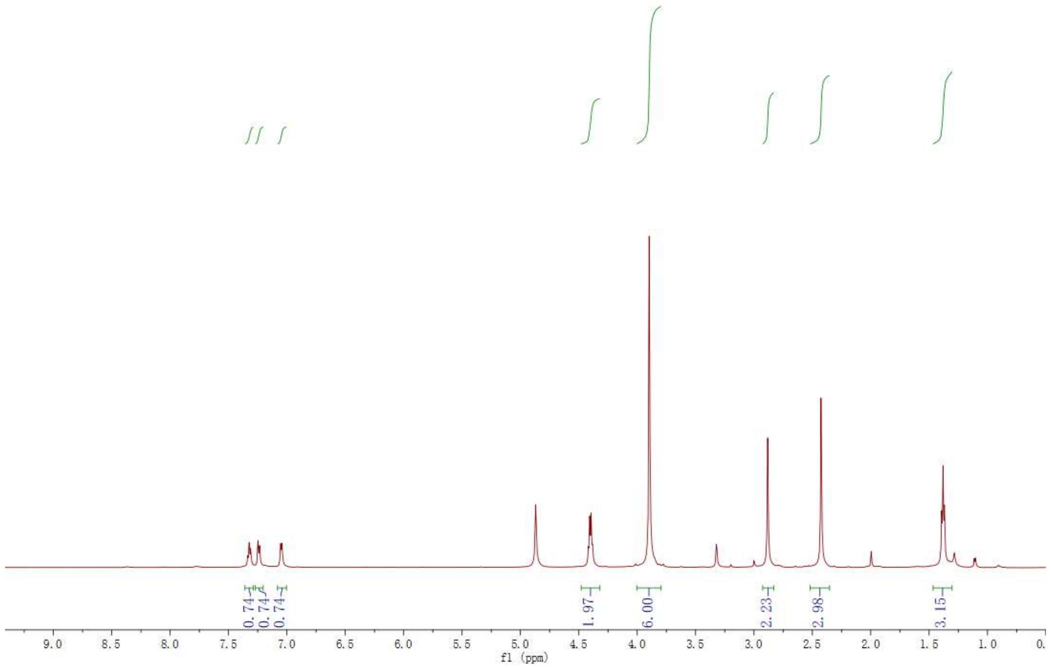

(B) X-ray Crystallographic Data

X-ray Single Crystal Structure Analysis of **3ac**

X-ray crystallographic data of **3ac** was solutions at T = 293(2) K: $C_{22}H_{21}ClO_4S$, *Mr* = 416.90, monoclinic. Space group *P-1*, a = 8.8958(3) Å, b = 8.9638(3) Å, c = 13.8898(6) Å, α = 98.8930°, β = 102.5920°, γ = 104.1250°, *V* = 1022.68(7) Å^3, *Z* = 2.

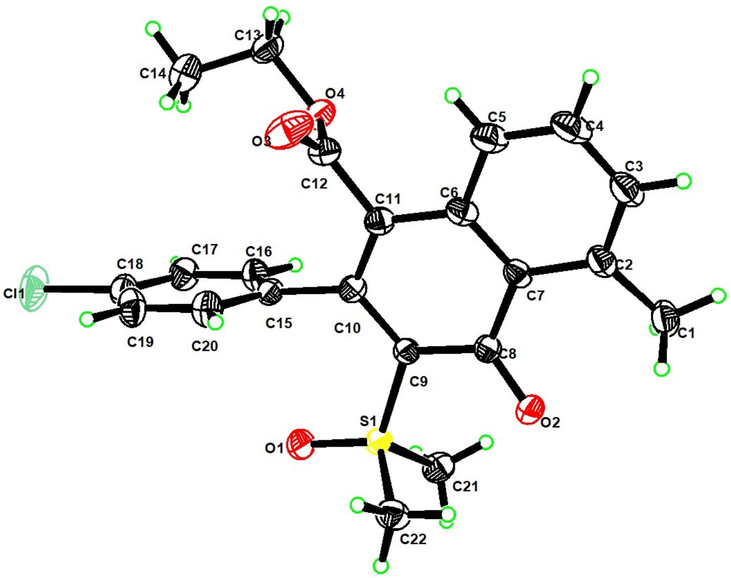

Figure S1: The crystal structure of **3ac** by X-ray analysis.

These data can be obtained free of charge from the Cambridge Crystallographic Data Centre via www.ccdc.cam.ac.uk/data_request/cif, the CCDC number is 1899265.

(C) Copies of ^{1}H NMR and ^{13}C NMR Spectra for the Products

Ethyl 3-(dimethyl(oxo)-λ^6-sulfanylidene)-5-methyl-4-oxo-2-phenyl-3,4-dihydronaphthalene-1-carboxylate (**3aa**)

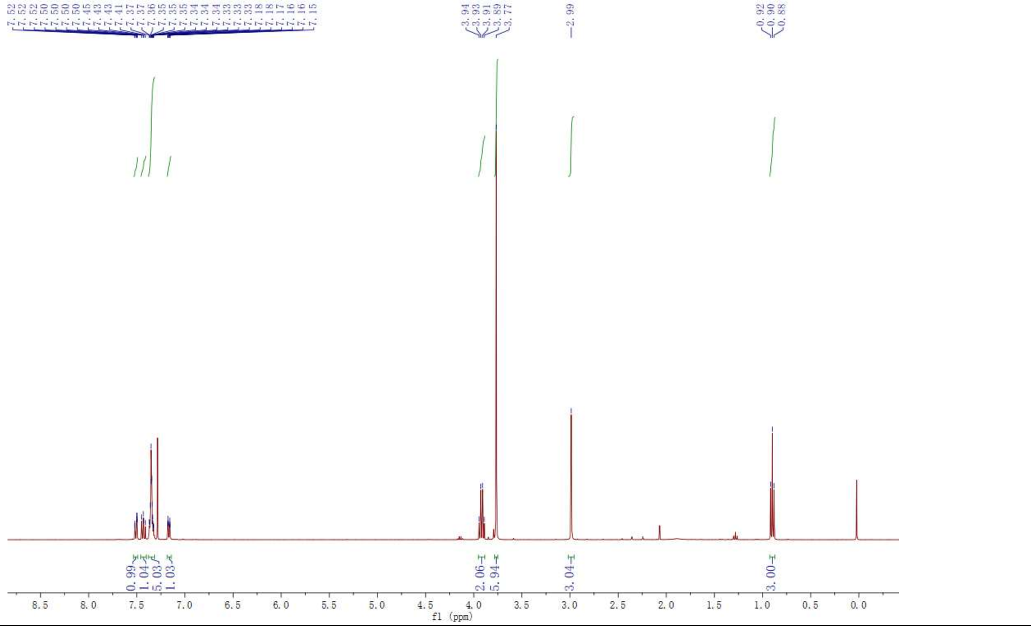

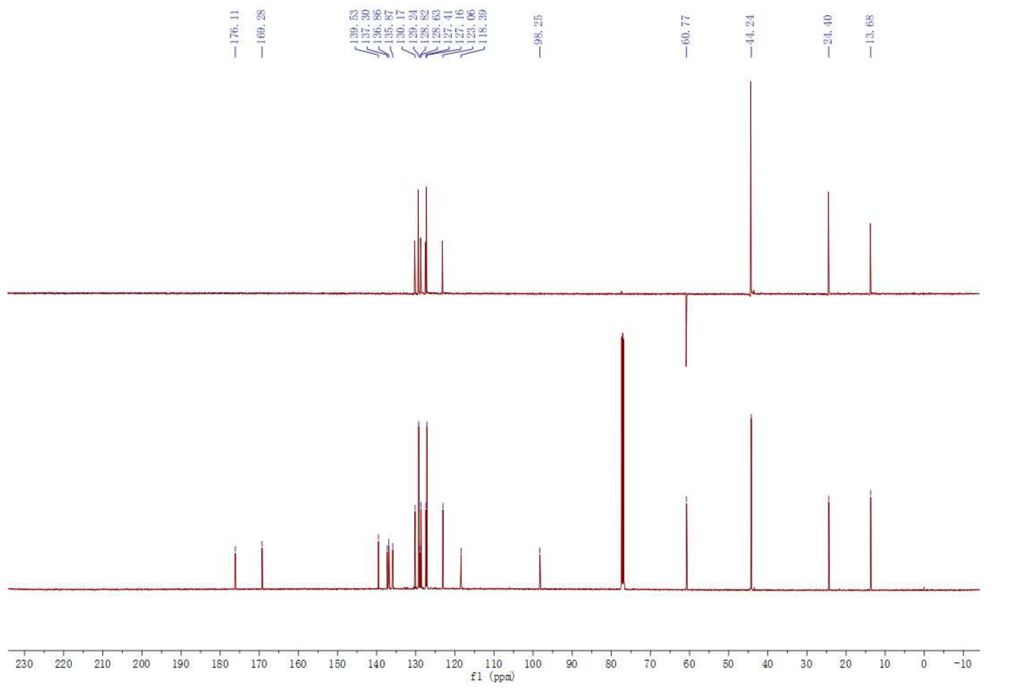

Ethyl 5-chloro-3-(dimethyl(oxo)-λ^6-sulfanylidene)-4-oxo-2-phenyl-3,4-dihydronaphthalene-1-carboxylate (**3ba**)

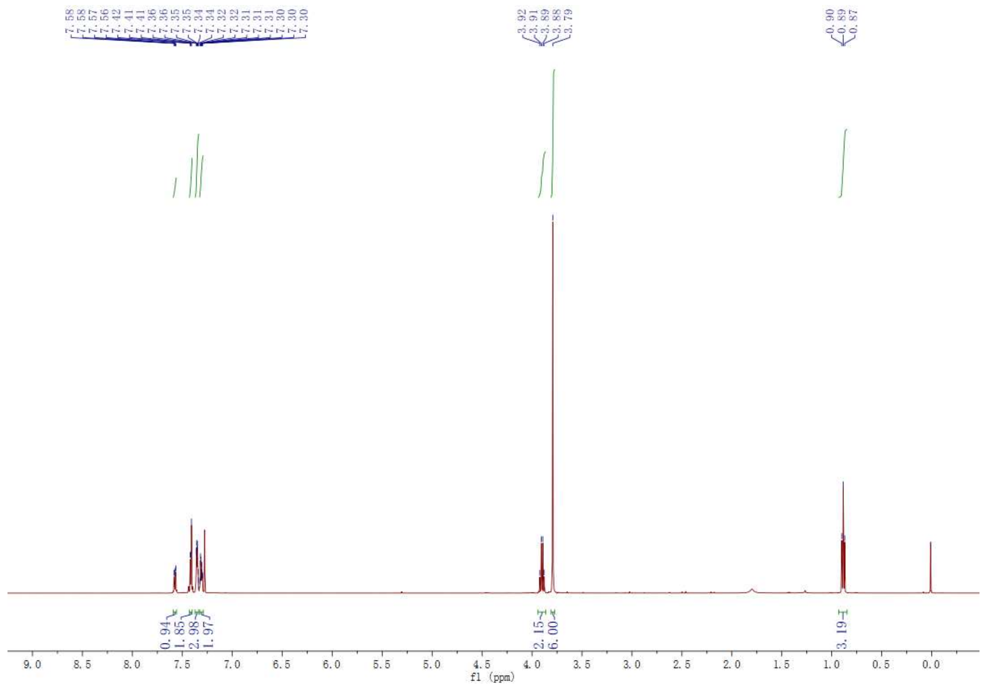

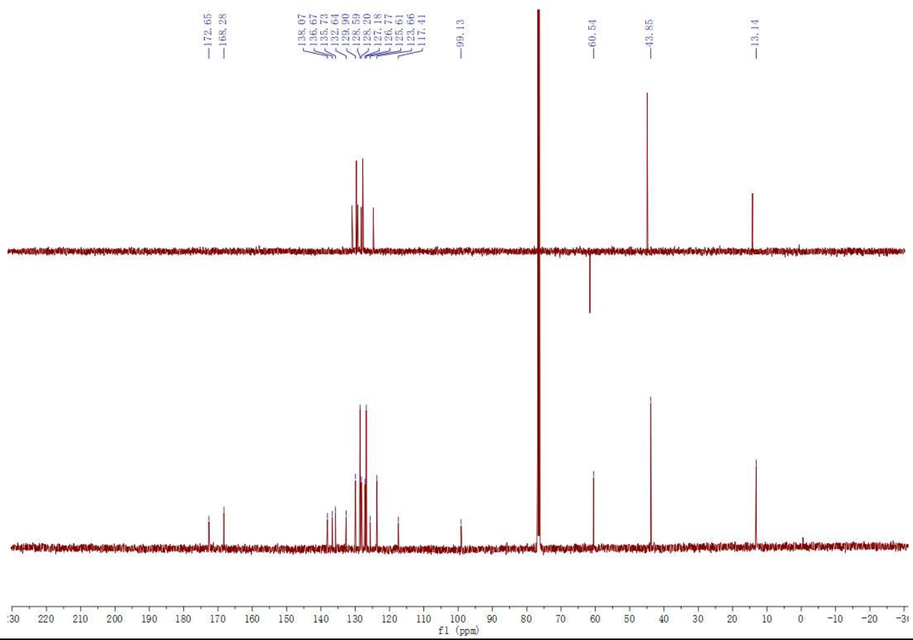

Ethyl 5-bromo-3-(dimethyl(oxo)-λ^6-sulfanylidene)-4-oxo-2-phenyl-3,4-dihydronaphthalene-1-carboxylate(**3ca**)

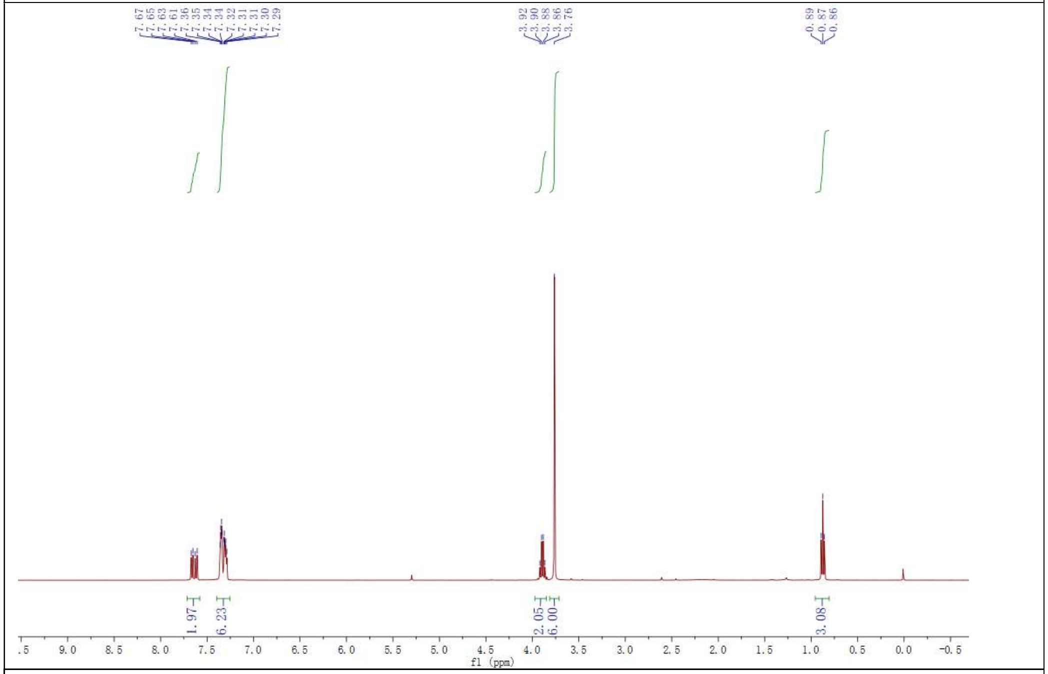

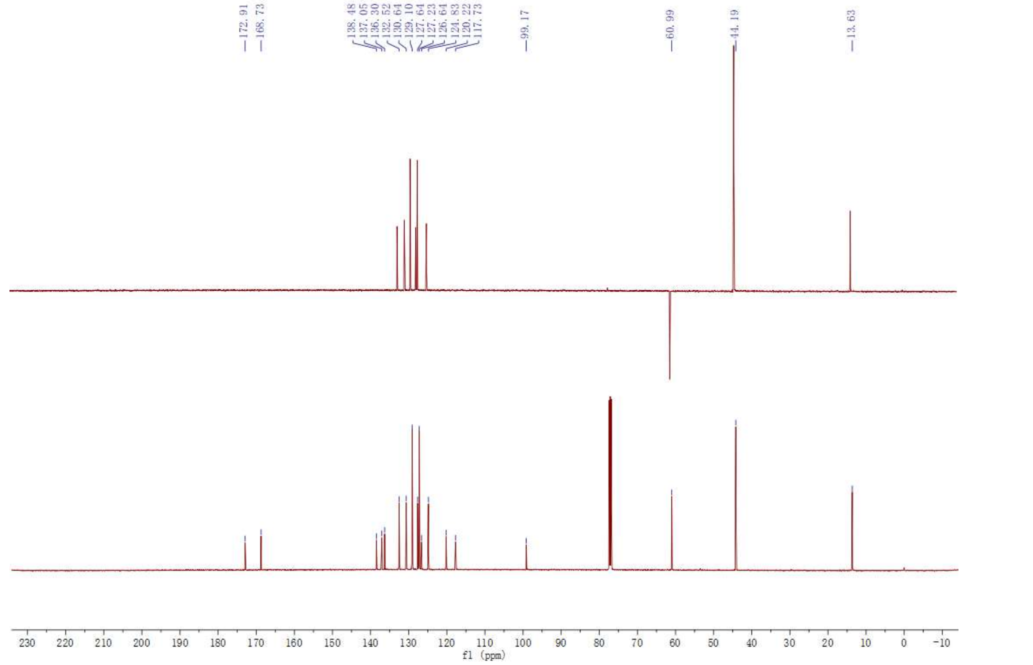

Ethyl 3-(dimethyl(oxo)-λ[6]-sulfanylidene)-4-oxo-2-phenyl-5-(trifluoromethyl)-3,4-dihydronaphthalene-1-carboxylate(**3da**)

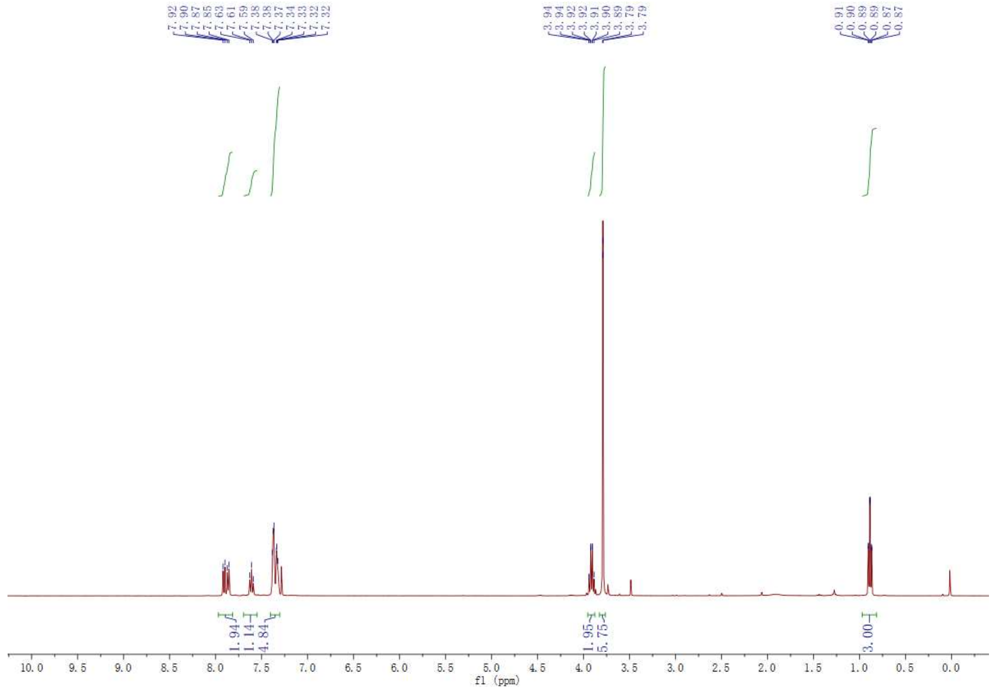

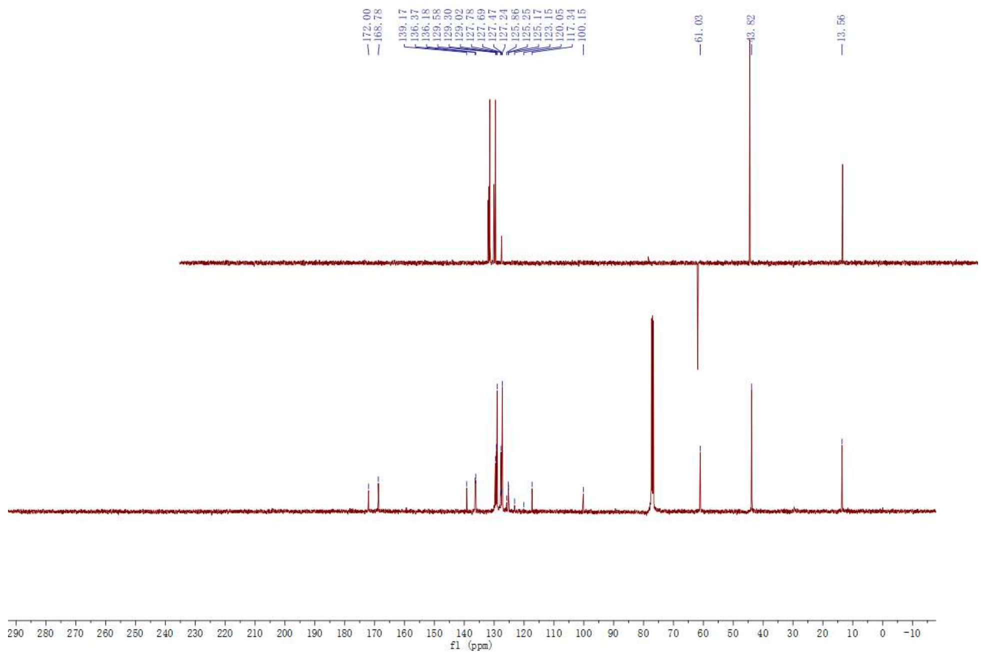

Ethyl 5-chloro-3-(dimethyl(oxo)-λ^6-sulfanylidene)-7-methyl-4-oxo-2-phenyl-3,4-dihydronaphthalene-1-carboxylate(**3ea**)

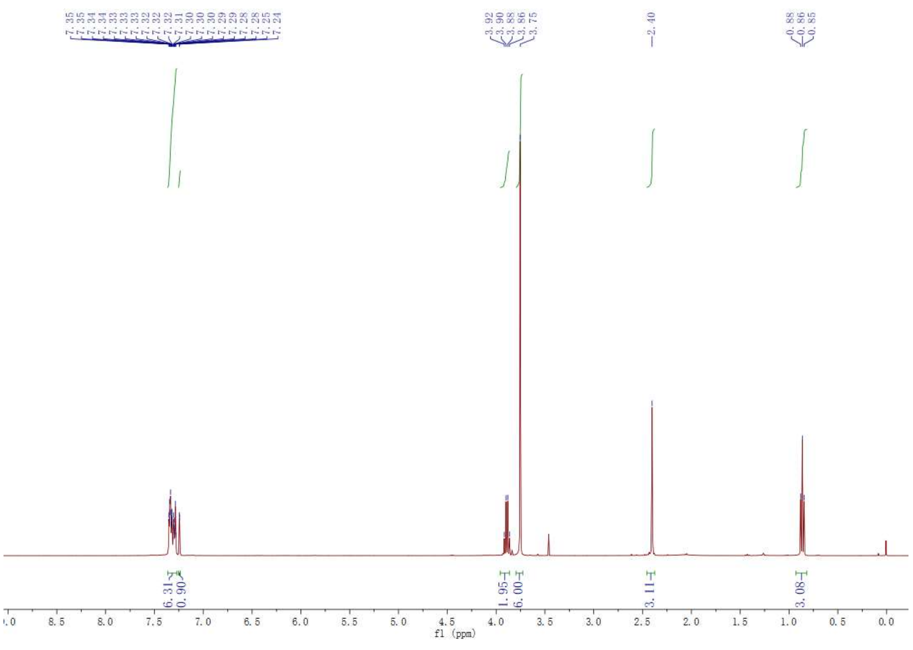

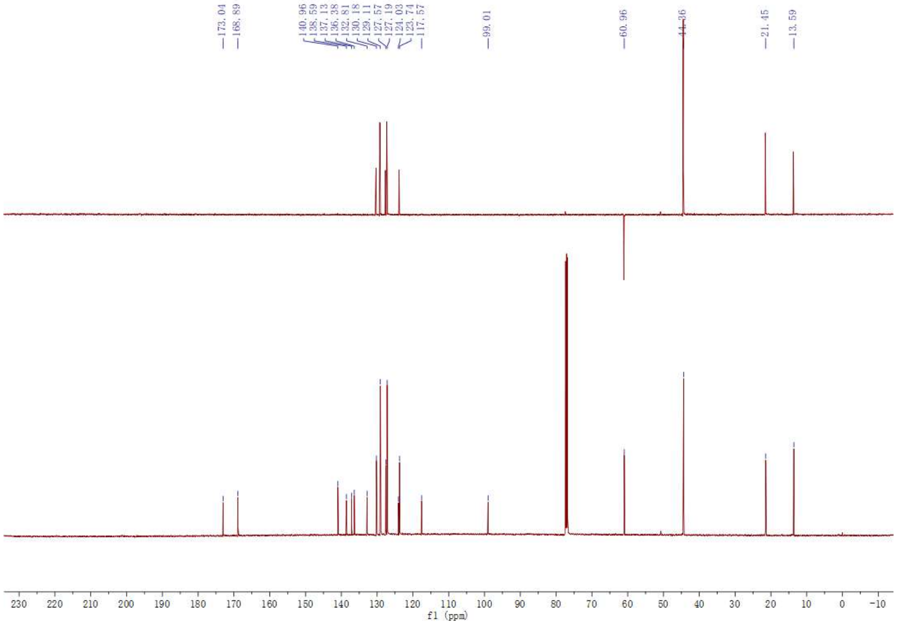

Ethyl 5-chloro-3-(dimethyl(oxo)-λ^6-sulfanylidene)-7-fluoro-4-oxo-2-phenyl-3,4-dihydronaphthalene-1-carboxylate(**3fa**)

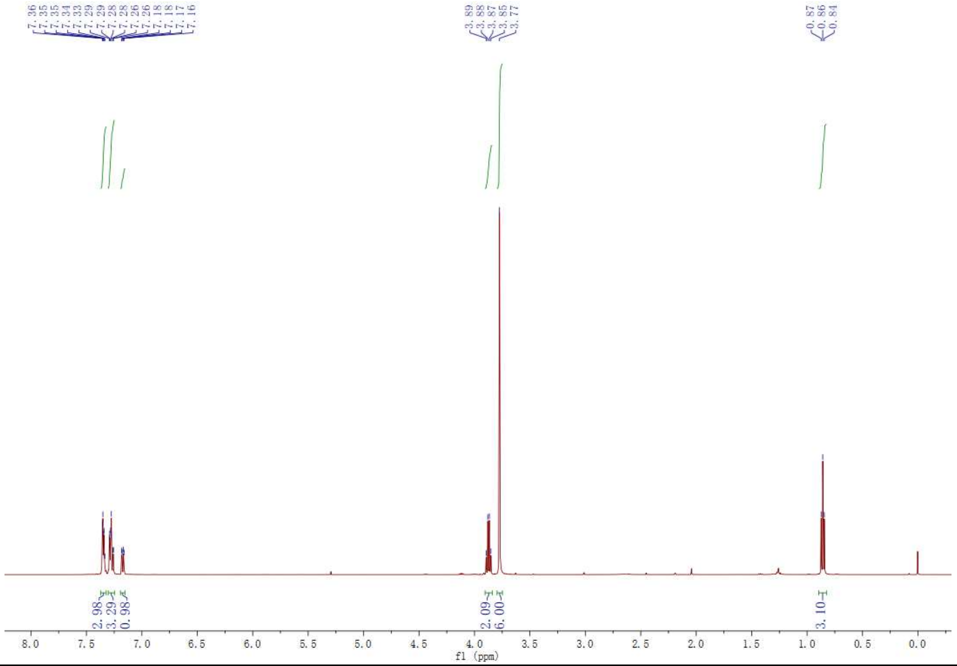

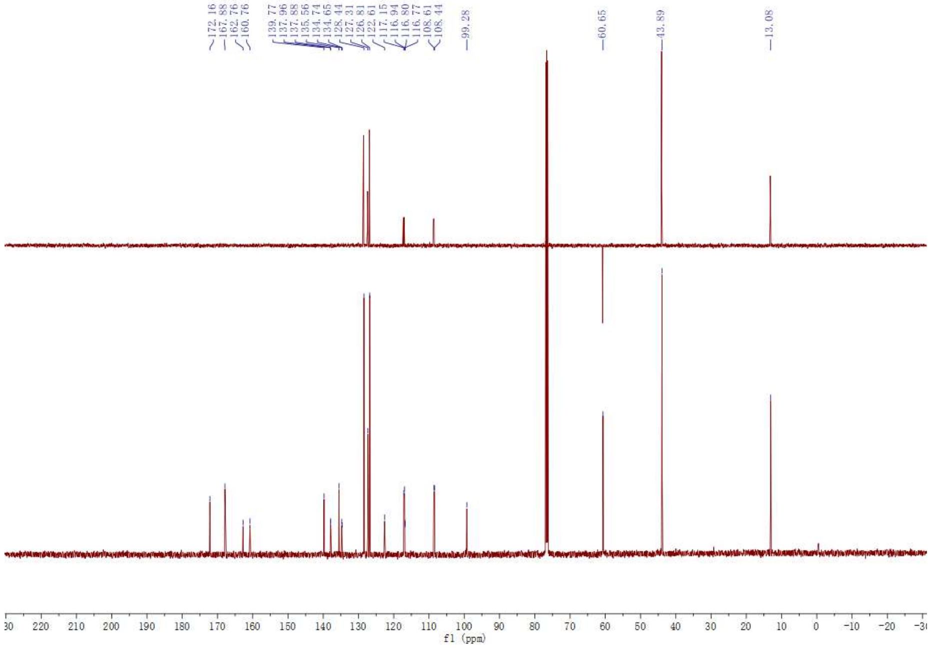

Ethyl 5,7-dichloro-3-(dimethyl(oxo)-λ^6-sulfanylidene)-4-oxo-2-phenyl-3,4-dihy-dro naphthalene-1-carboxylate(**3ga**)

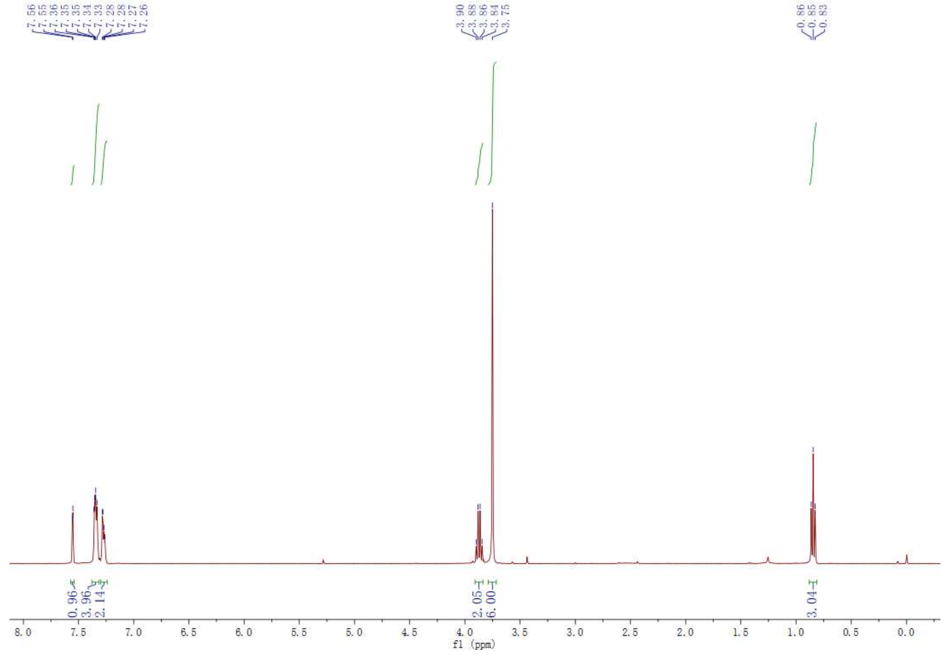

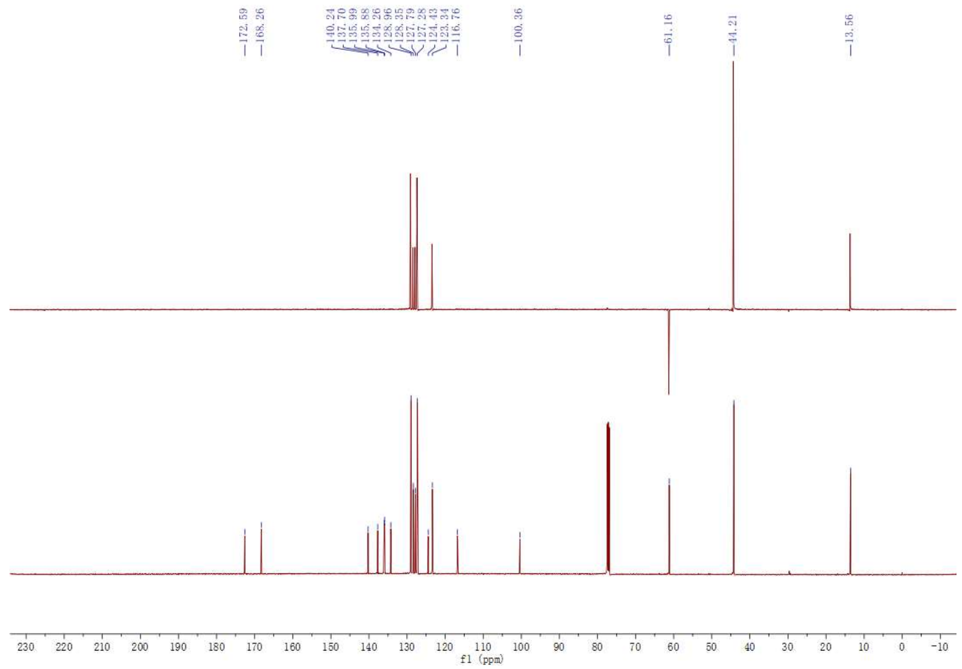

Ethyl 5-chloro-3-(dimethyl(oxo)-λ^6-sulfanylidene)-7-methoxy-4-oxo-2-phenyl- 3,4-dihydronaphthalene-1-carboxylate(**3ha**)

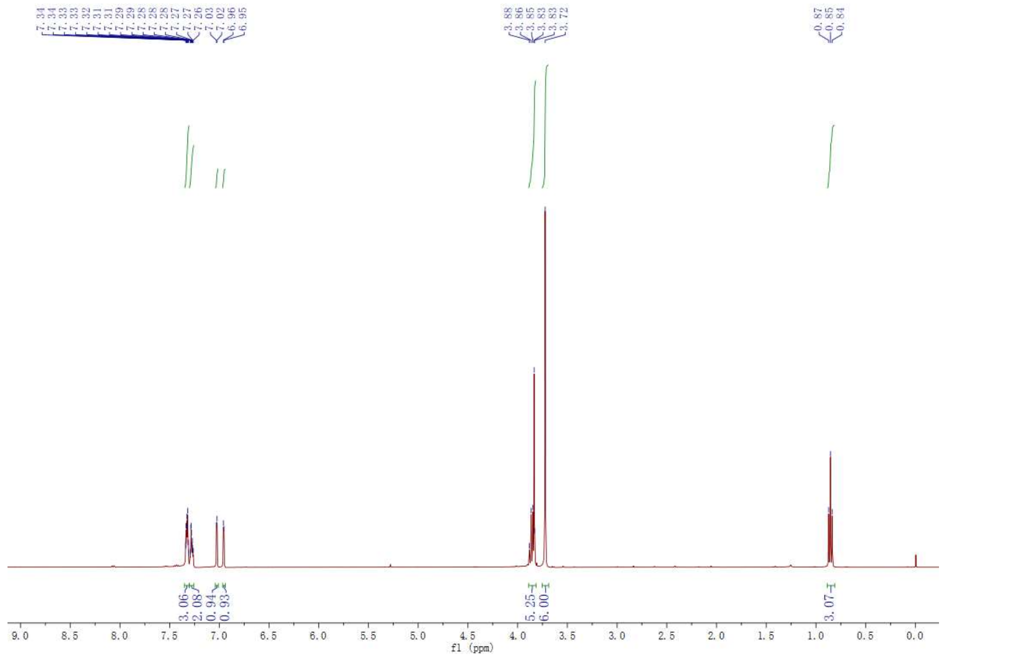

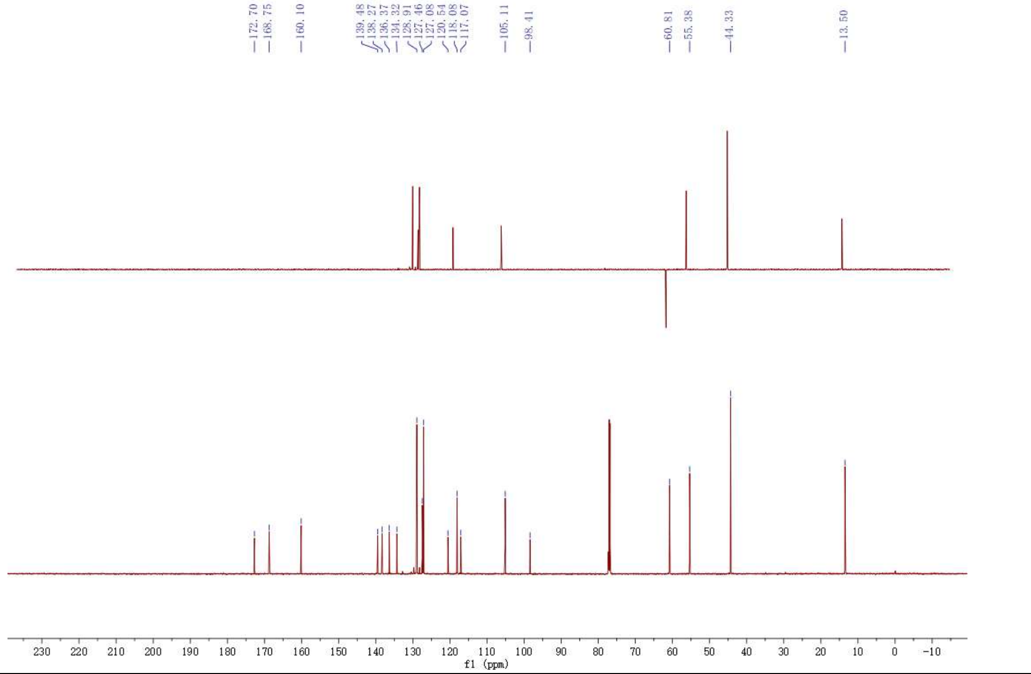

Ethyl 5-chloro-3-(dimethyl(oxo)-λ^6-sulfanylidene)-4-oxo-2-phenyl-7-(trifluoromethyl)-3,4-dihydronaphthalene-1-carboxylate(**3ia**)

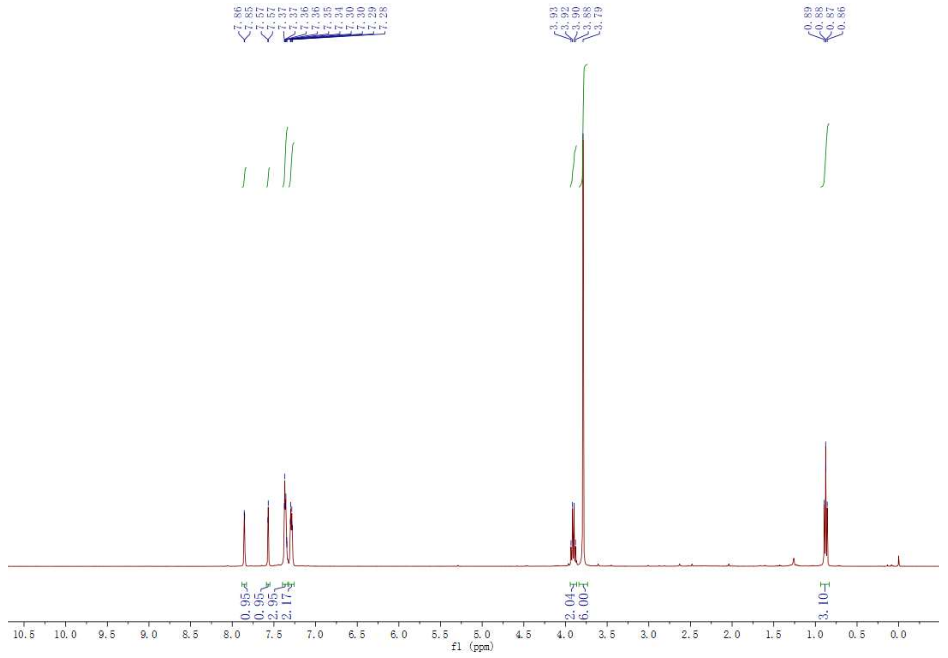

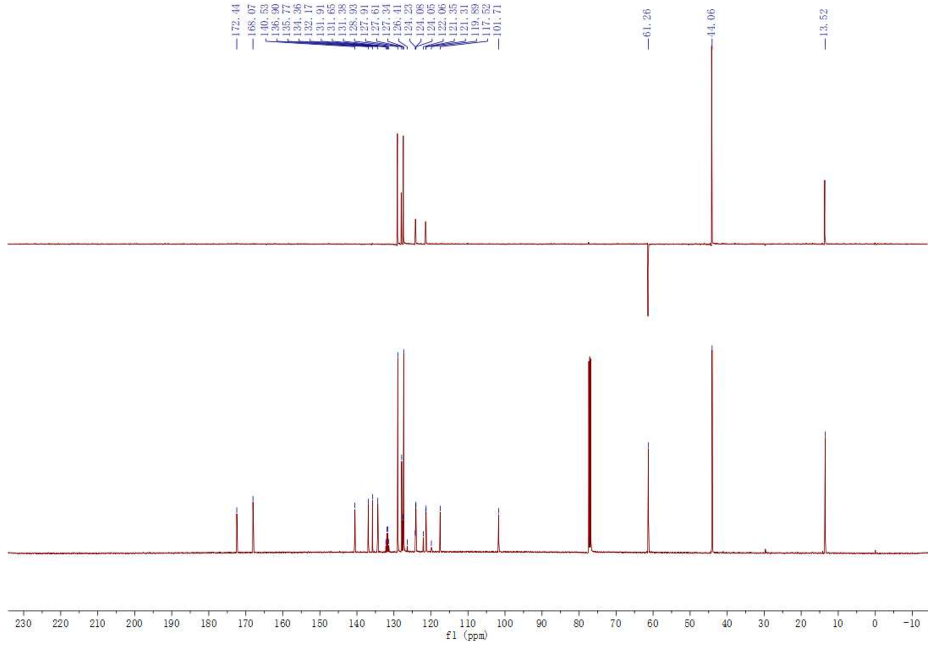

Ethyl 6-bromo-5-chloro-3-(dimethyl(oxo)-λ^6-sulfanylidene)-4-oxo-2-phenyl-3,4-dihydronaphthalene-1-carboxylate (**3ja**)

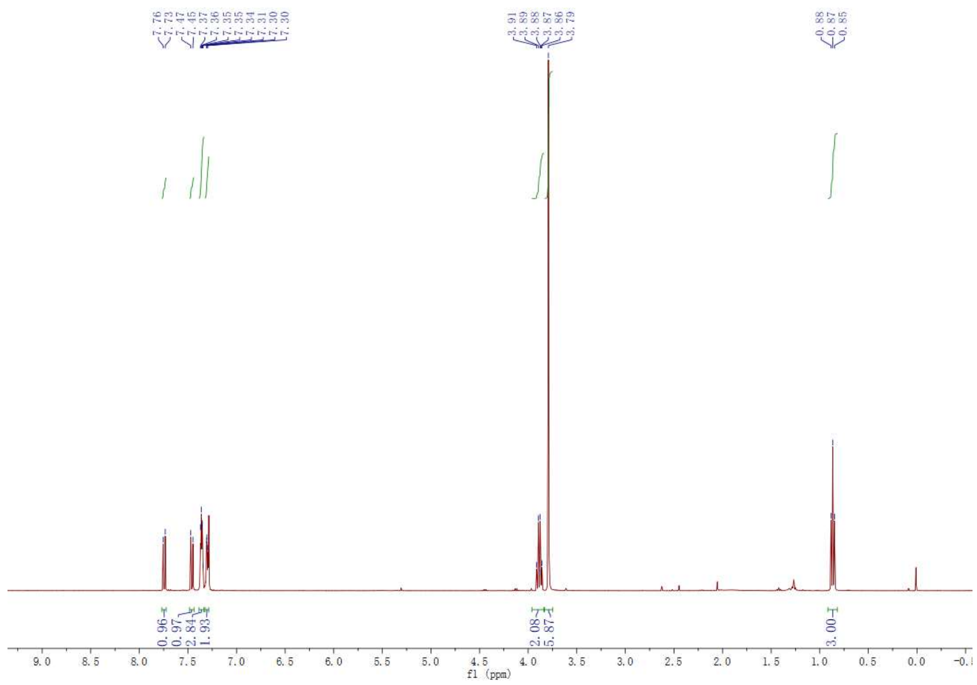

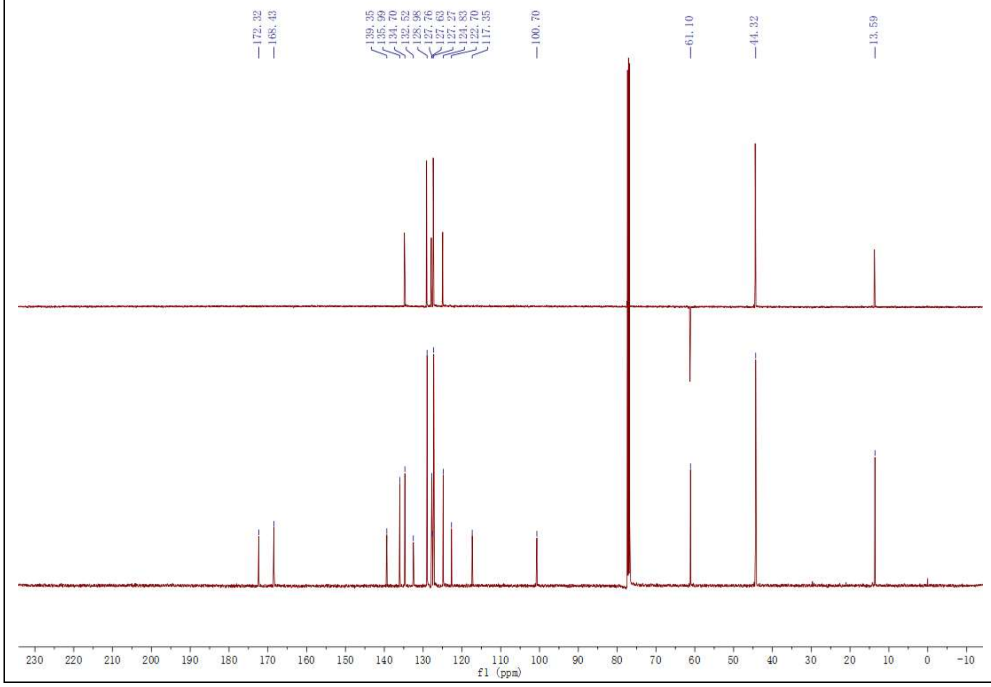

Ethyl 5-chloro-3-(dimethyl(oxo)-λ^{6}-sulfanylidene)-6-methyl-4-oxo-2-phenyl-3,4-dihydronaphthalene-1-carboxylate (**3ka**)

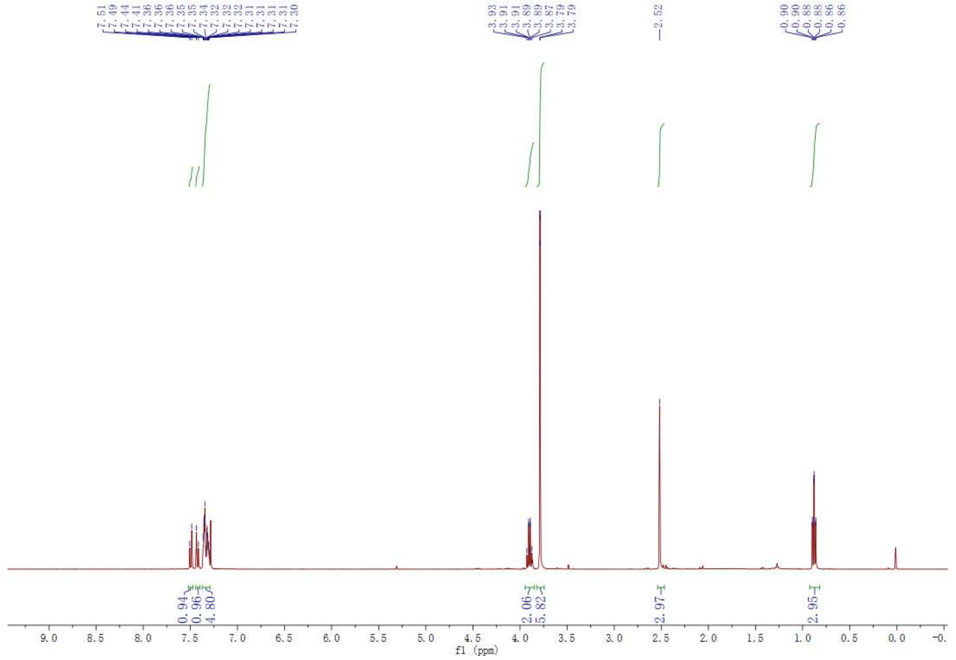

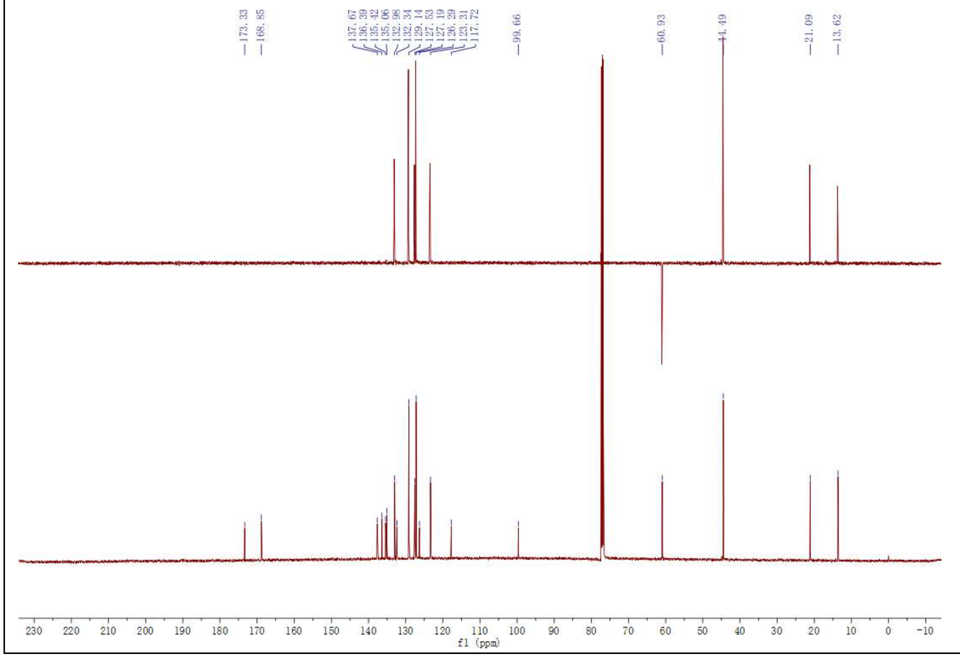

Ethyl 5-chloro-3-(dimethyl(oxo)-λ^6-sulfanylidene)-8-methoxy-4-oxo-2-phenyl-3,4-dihydronaphthalene-1-carboxylate (**3la**)

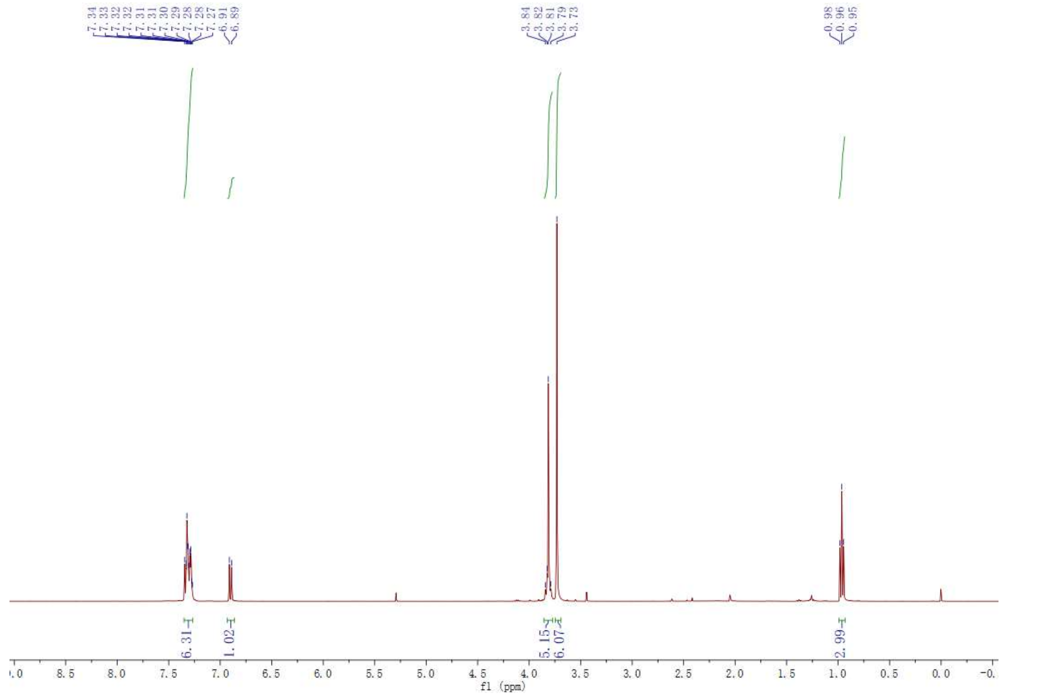

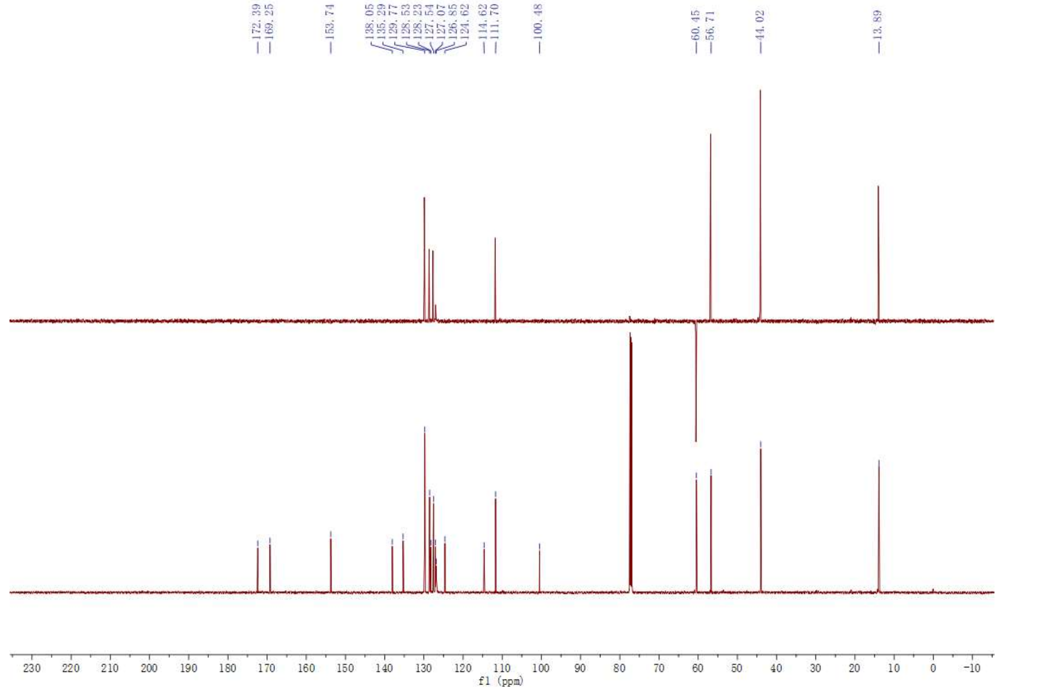

Ethyl 3-(dimethyl(oxo)-λ6-sulfanylidene)-5-(1-ethoxy-1,3-dioxo-3-phenylpro pan-2-yl)-4-oxo-2-phenyl-3,4-dihydronaphthalene-1-carboxylate(**3ma**)

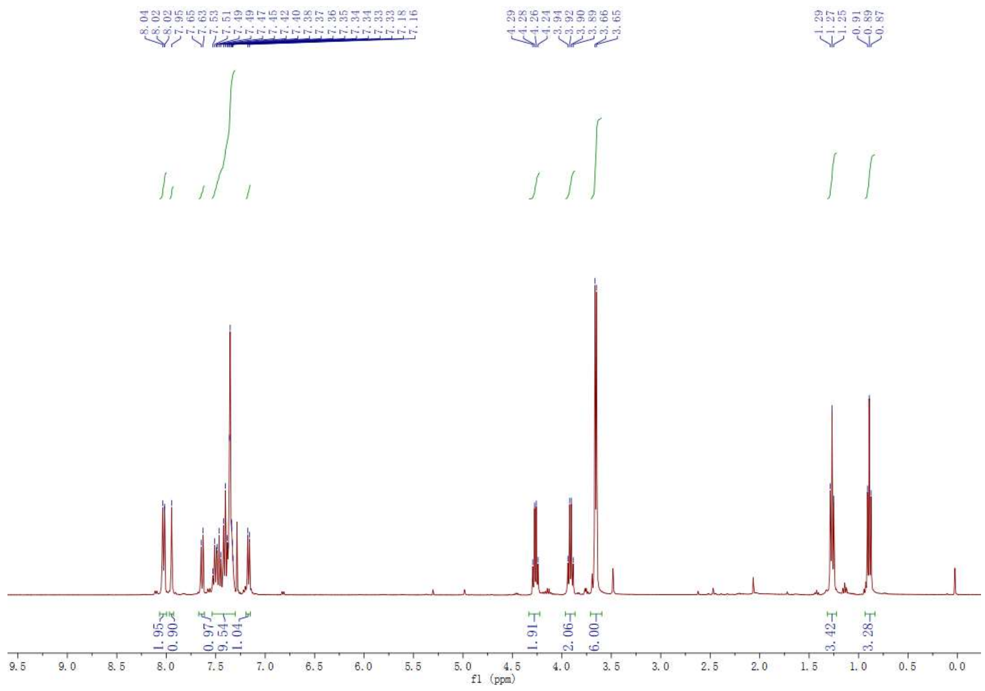

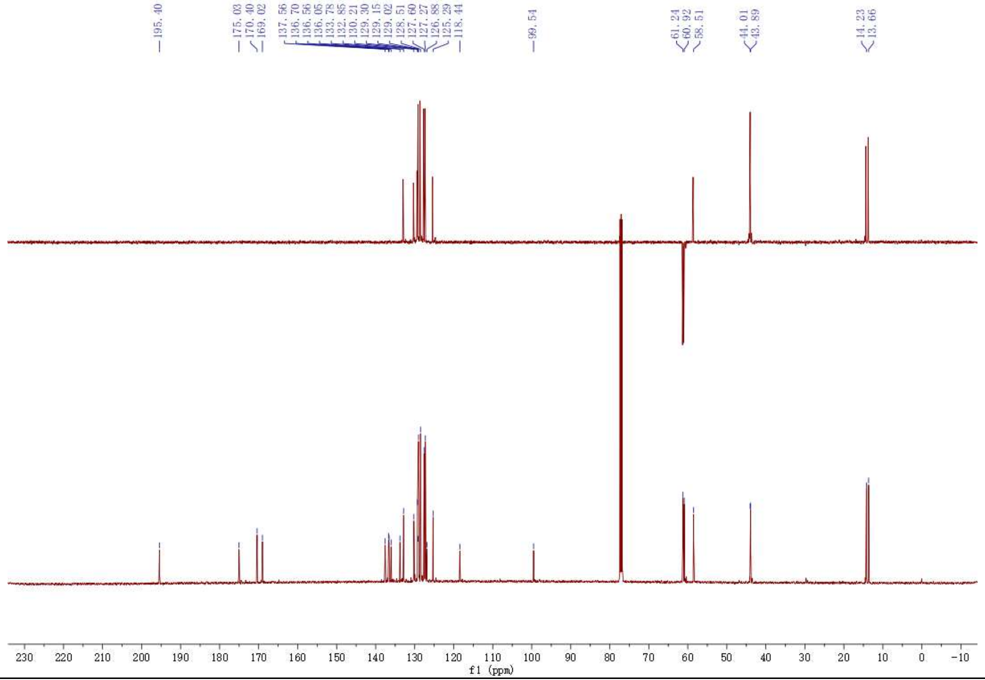

Ethyl 3-(dimethyl(oxo)-λ[6]-sulfanylidene)-5-(1-ethoxy-1,3-dioxo-3-phenylpropan -2-yl)-7-methoxy-4-oxo-2-phenyl-3,4-dihydronaphthalene-1-carboxylate (**3na**)

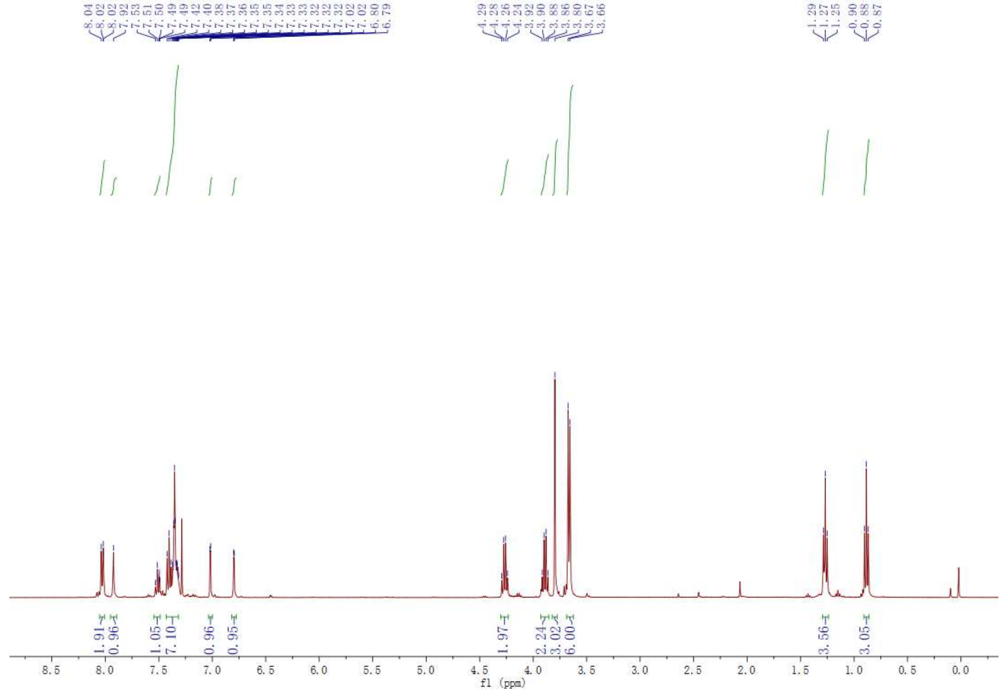

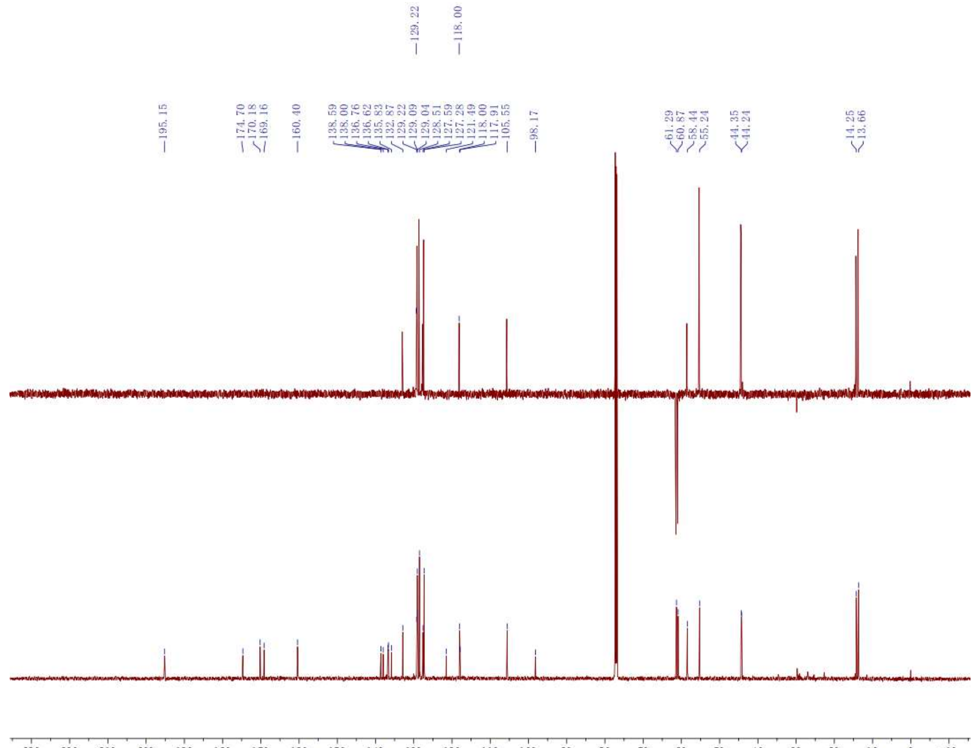

Ethyl 7-(tert-butyl)-3-(dimethyl(oxo)-l6-sulfanylidene)-5-(1-ethoxy-1,3-dioxo-3-phenylpropan-2-yl)-4-oxo-2-phenyl-3,4-dihydronaphthalene-1-carboxylate (**3oa**)

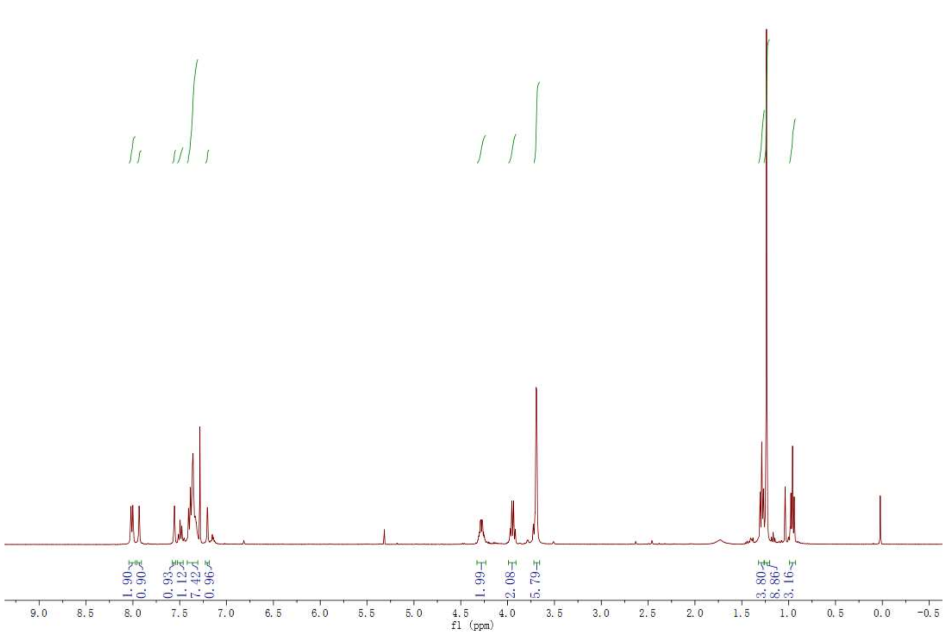

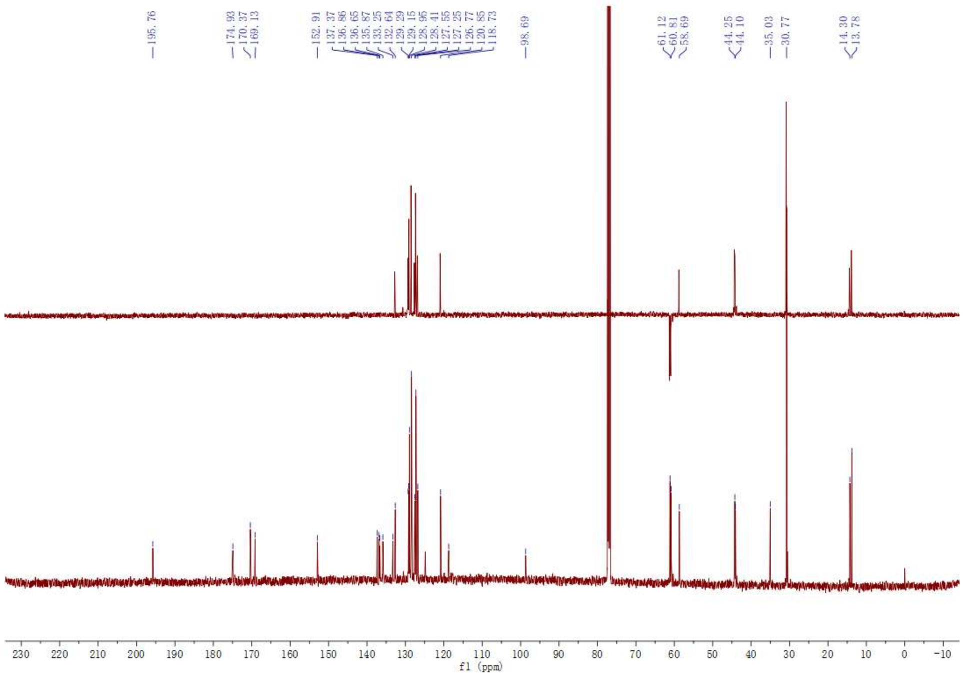

Ethyl 7-bromo-3-(dimethyl(oxo)-l6-sulfanylidene)-5-(1-ethoxy-1,3-dioxo-3-phenylpropan-2-yl)-4-oxo-2-phenyl-3,4-dihydronaphthalene-1-carboxylate (**3pa**)

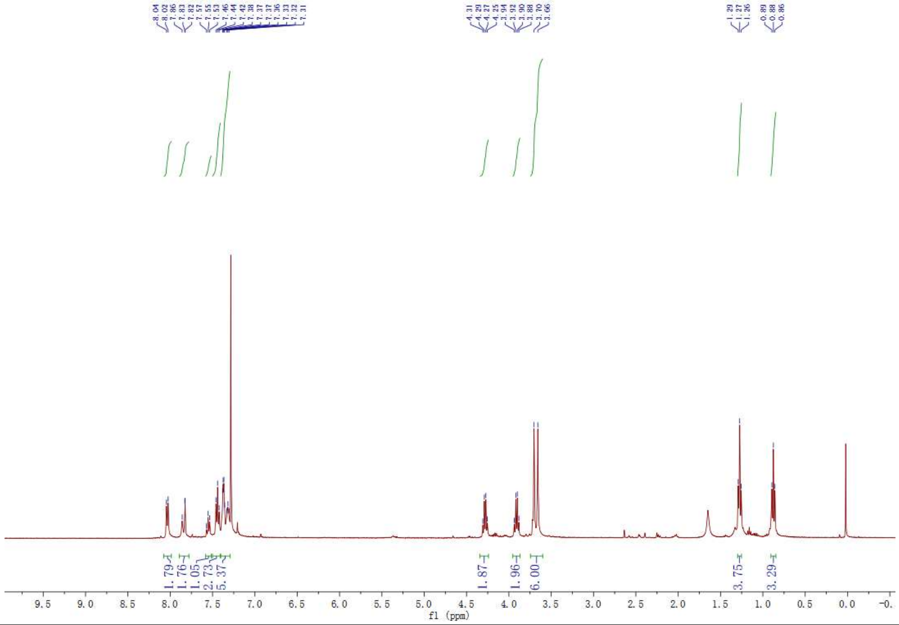

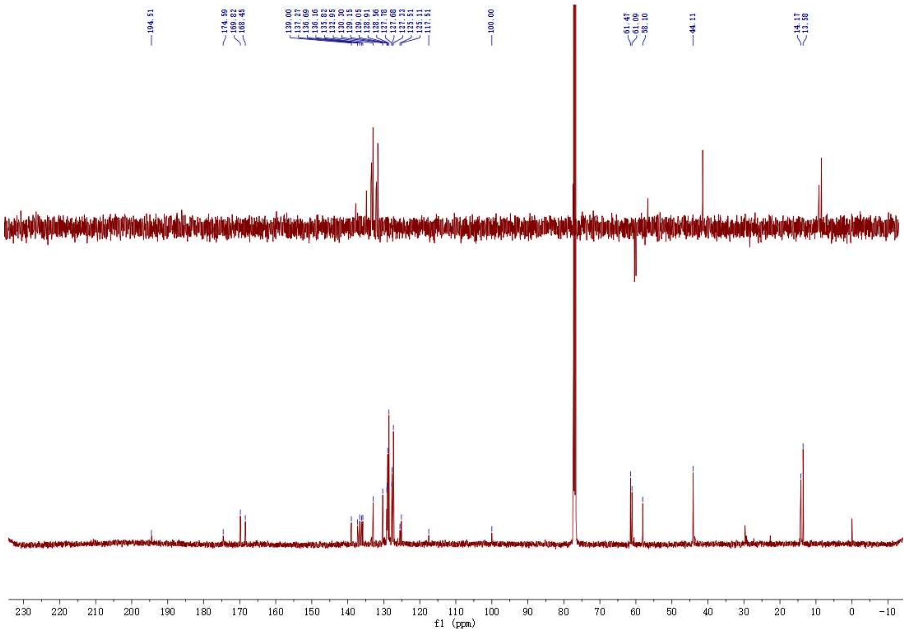

Ethyl 3-(dimethyl(oxo)-λ^6-sulfanylidene)-2-(4-fluorophenyl)-5-methyl-4-oxo-3,4-dihydronaphthalene-1-carboxylate(**3ab**)

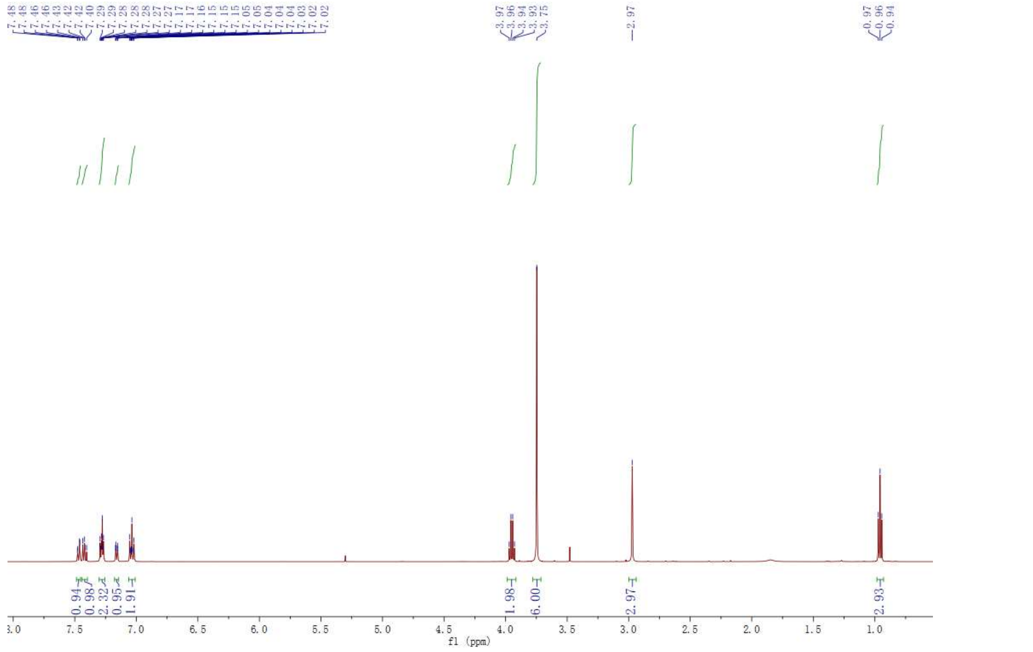

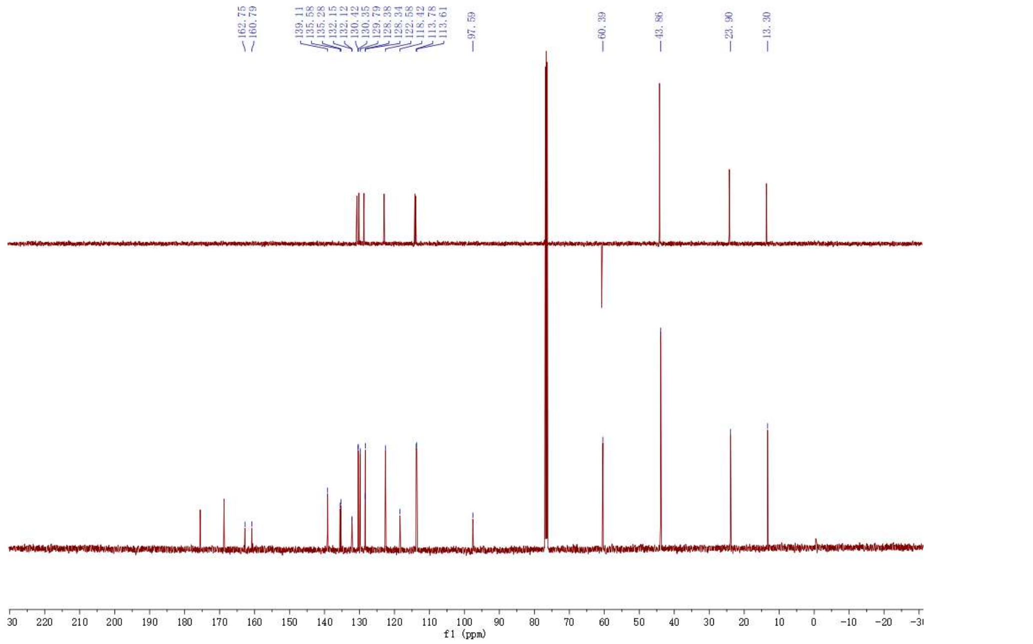

Ethyl 2-(4-chlorophenyl)-3-(dimethyl(oxo)-λ^6-sulfanylidene)-5-methyl-4-oxo-3,4-dihydronaphthalene-1-carboxylate(**3ac**)

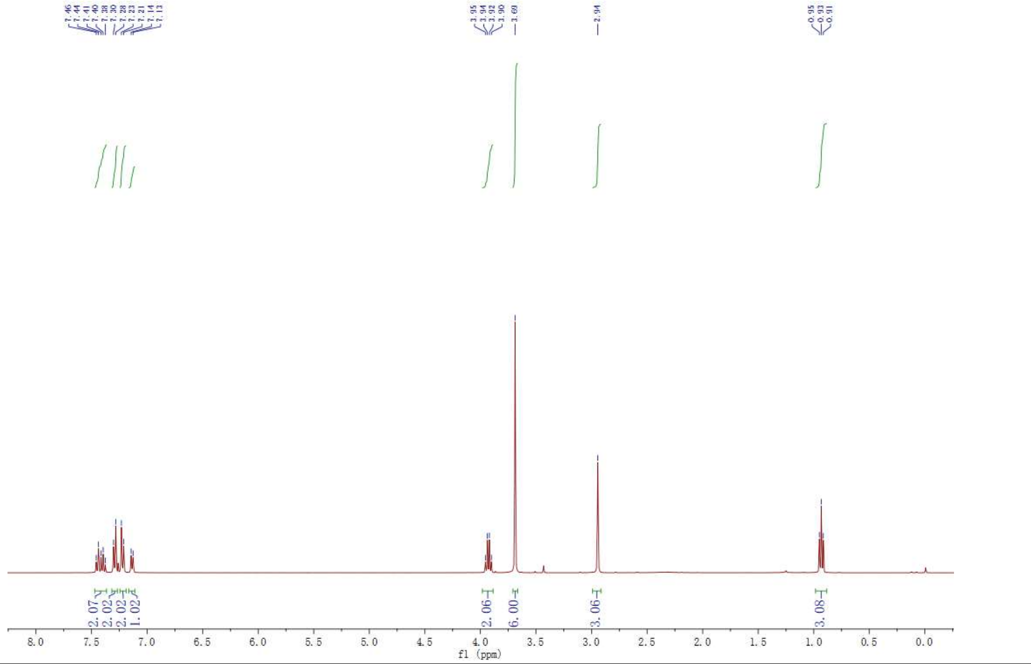

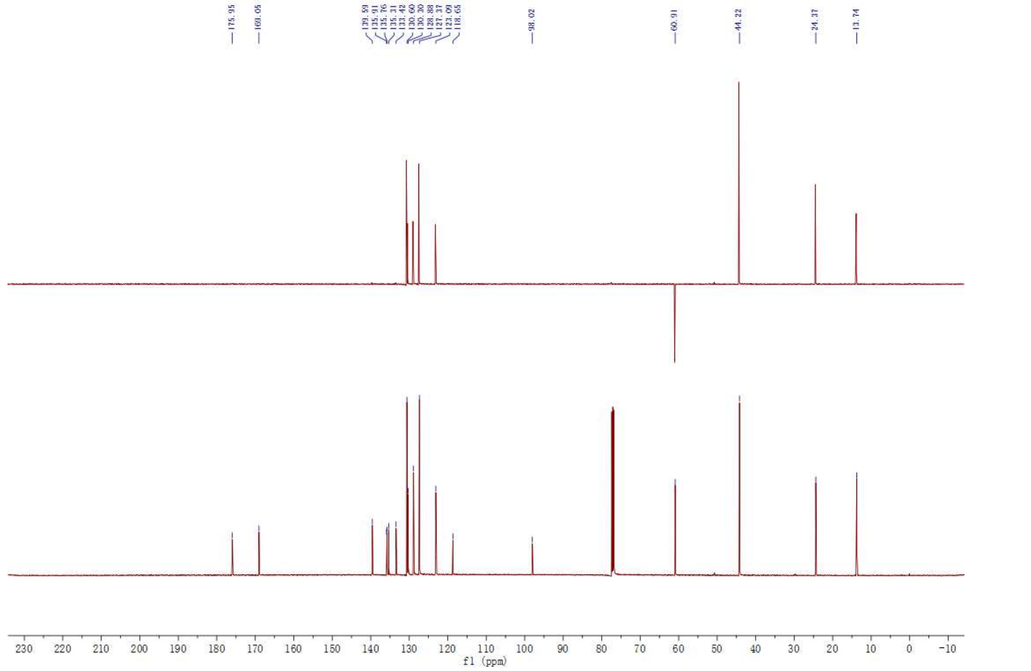

Ethyl 2-(4-bromophenyl)-3-(dimethyl(oxo)-λ^6-sulfanylidene)-5-methyl-4-oxo-3,4-dihydronaphthalene-1-carboxylate(**3ad**)

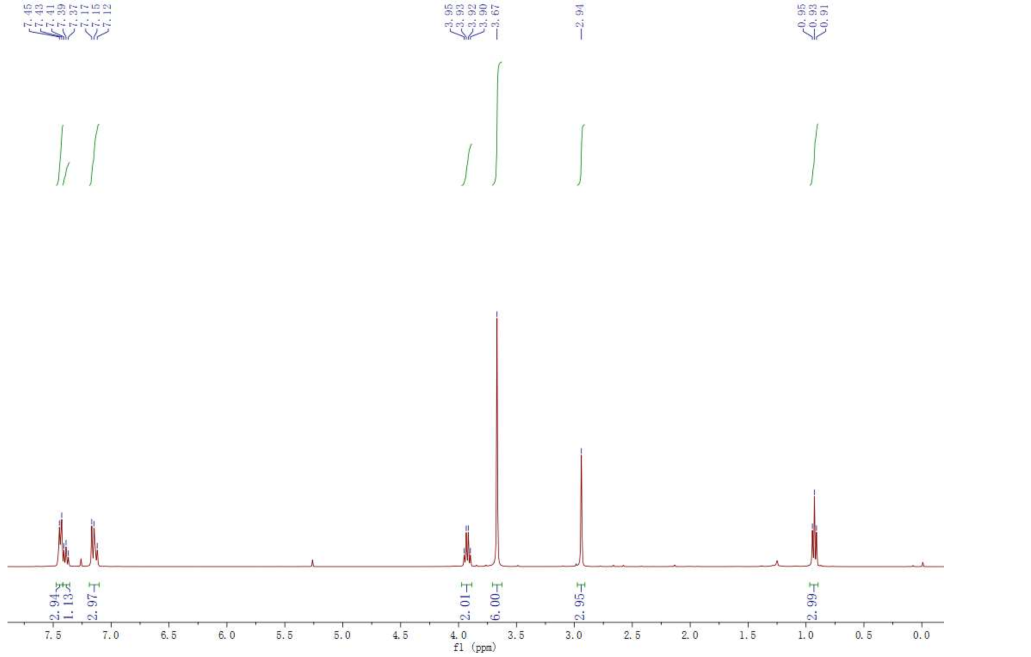

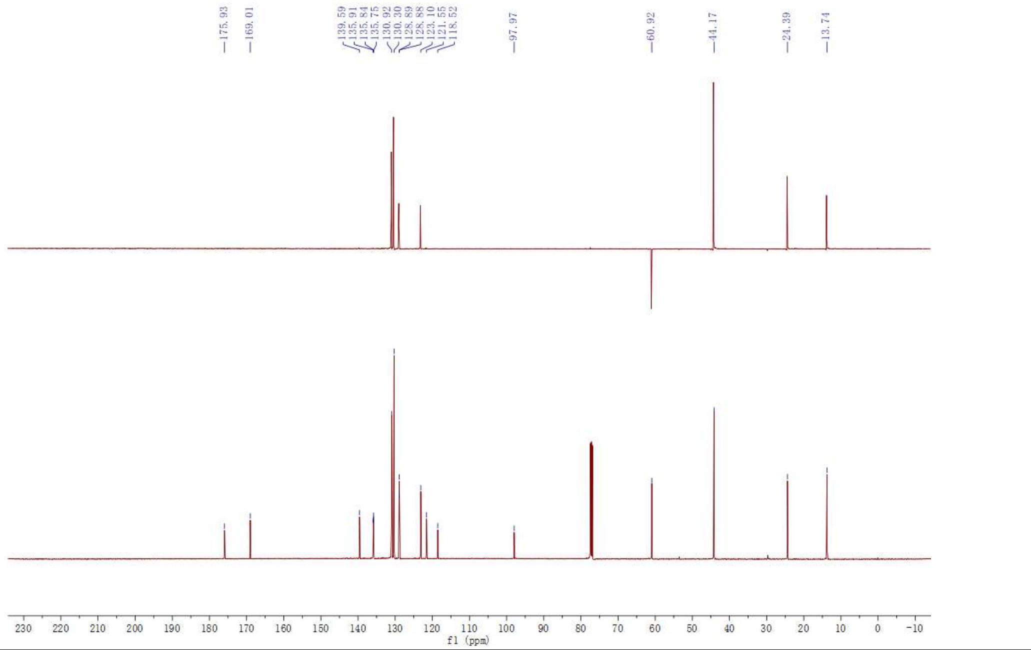

Ethyl 3-(dimethyl(oxo)-λ[6]-sulfanylidene)-5-methyl-4-oxo-2-(4-(trifluoromethyl) ph-enyl)-3,4-dihydronaphthalene-1-carboxylate(**3ae**)

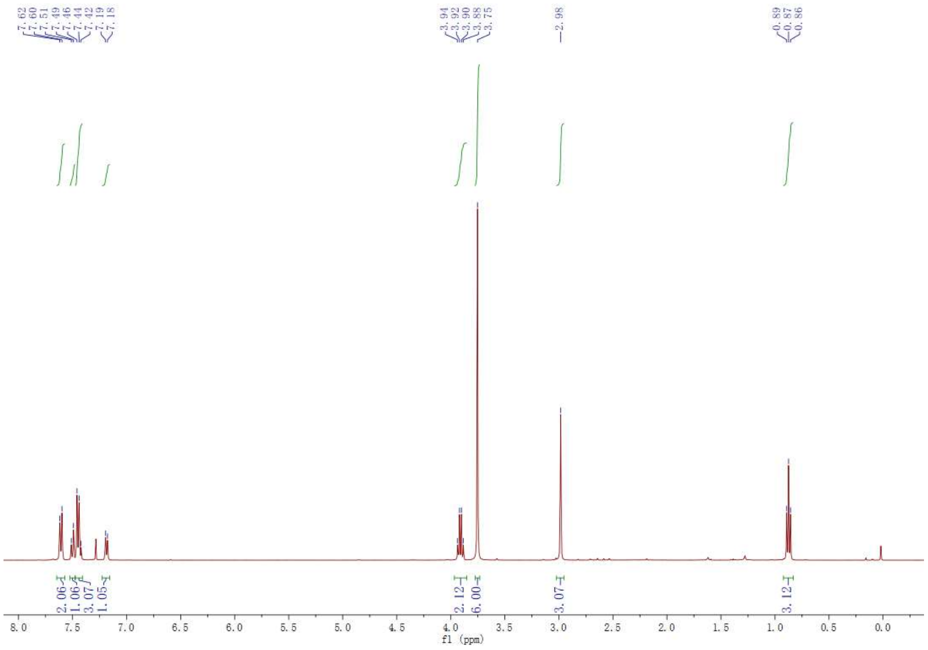

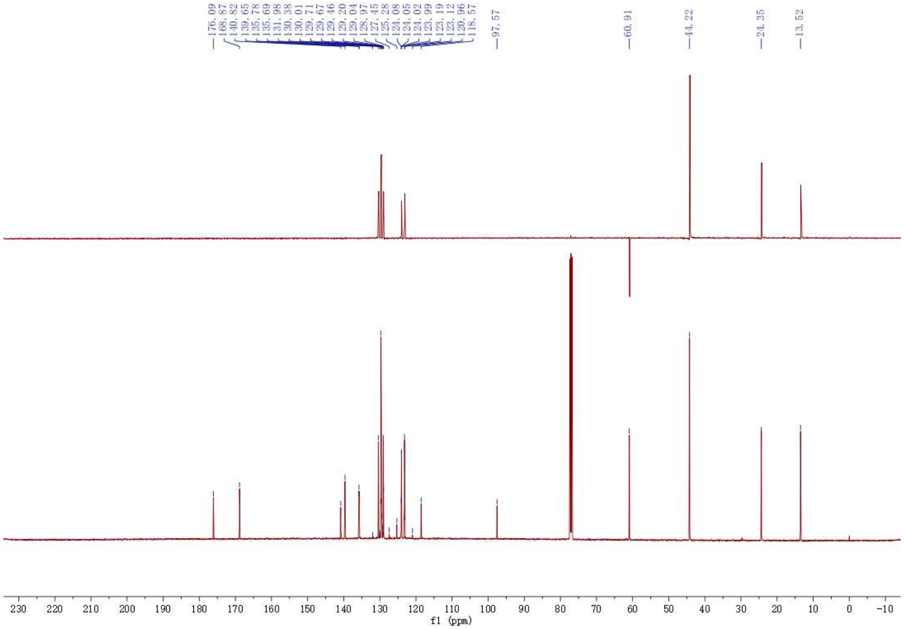

Ethyl 3-(dimethyl(oxo)-λ[6]-sulfanylidene)-2-(4-methoxyphenyl)-5-methyl-4-oxo-3,4-dihydronaphthalene-1-carboxylate(**3af**)

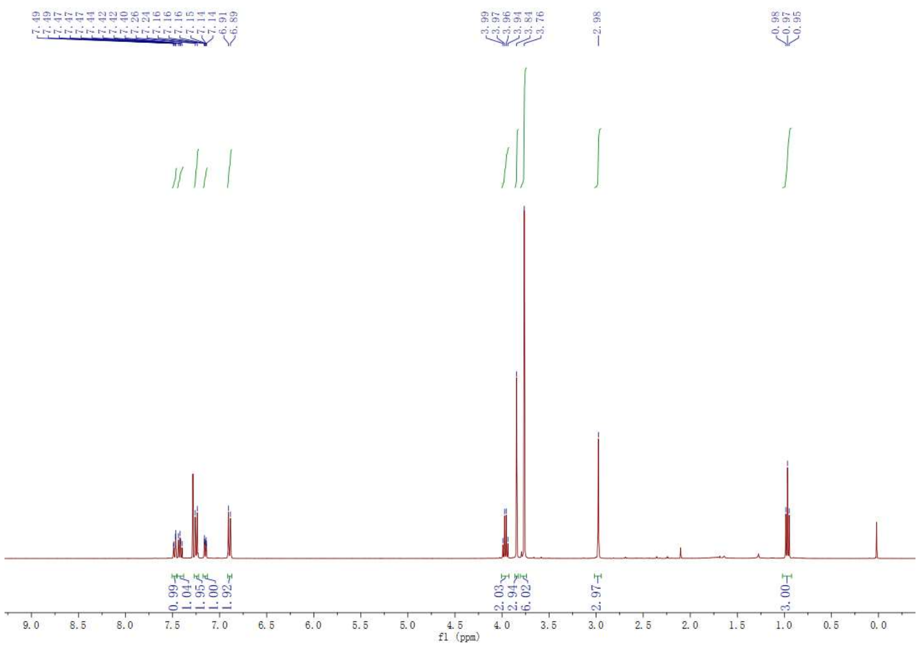

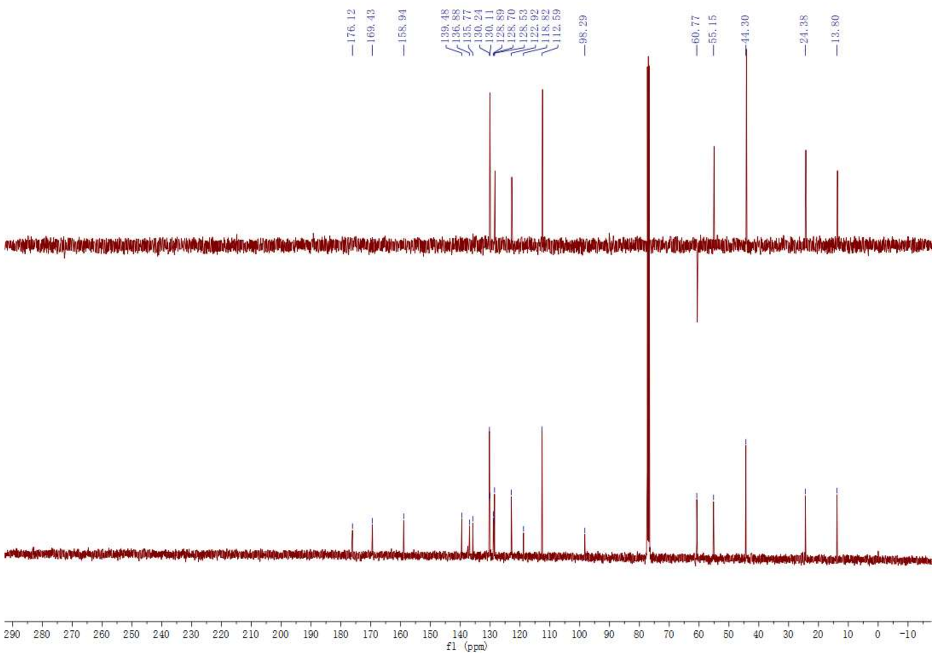

Ethyl 3-(dimethyl(oxo)-λ^6-sulfanylidene)-2-(3-methoxyphenyl)-5-methyl-4-oxo-3,4-dihydronaphthalene-1-carboxylate (**3ag**)

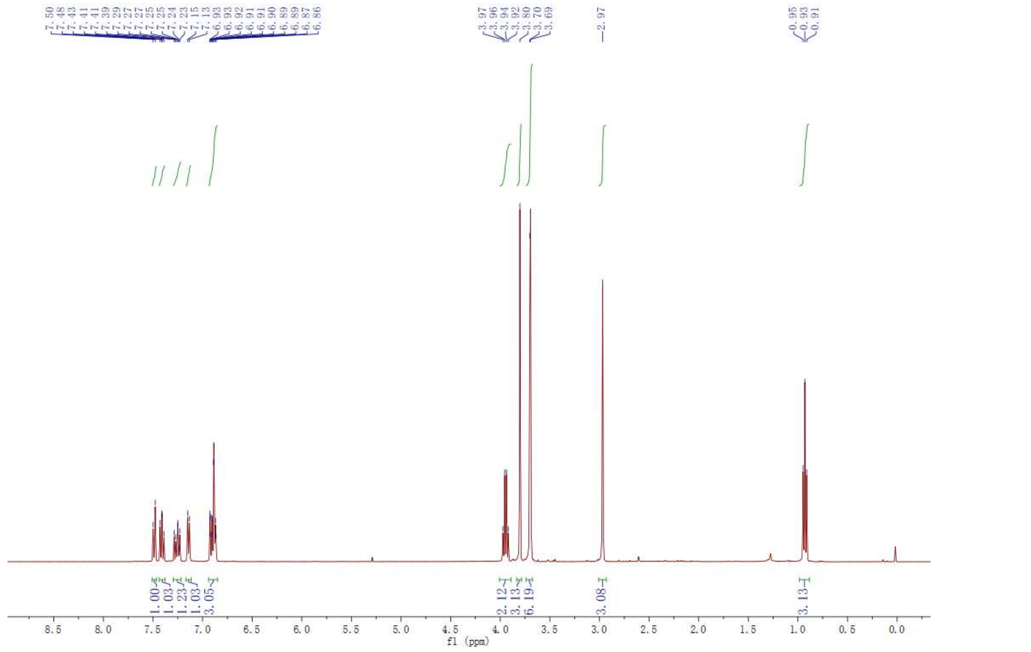

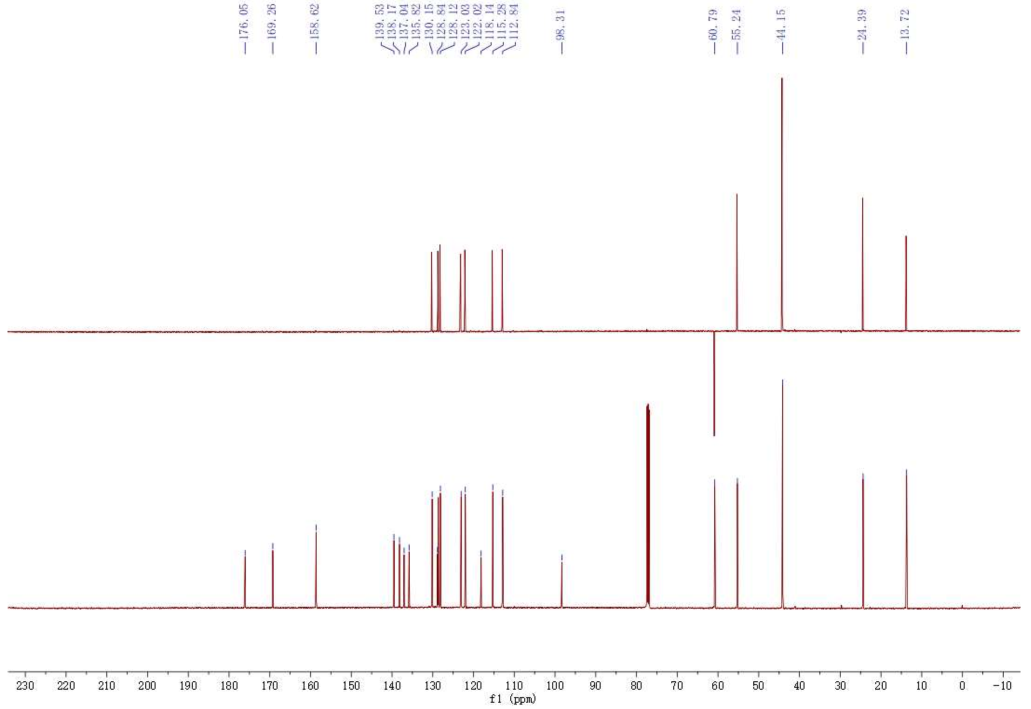

Ethyl 2-(3-bromophenyl)-3-(dimethyl(oxo)-λ^6-sulfanylidene)-5-methyl-4-oxo- 3,4-dihydronaphthalene-1-carboxylate (**3ah**)

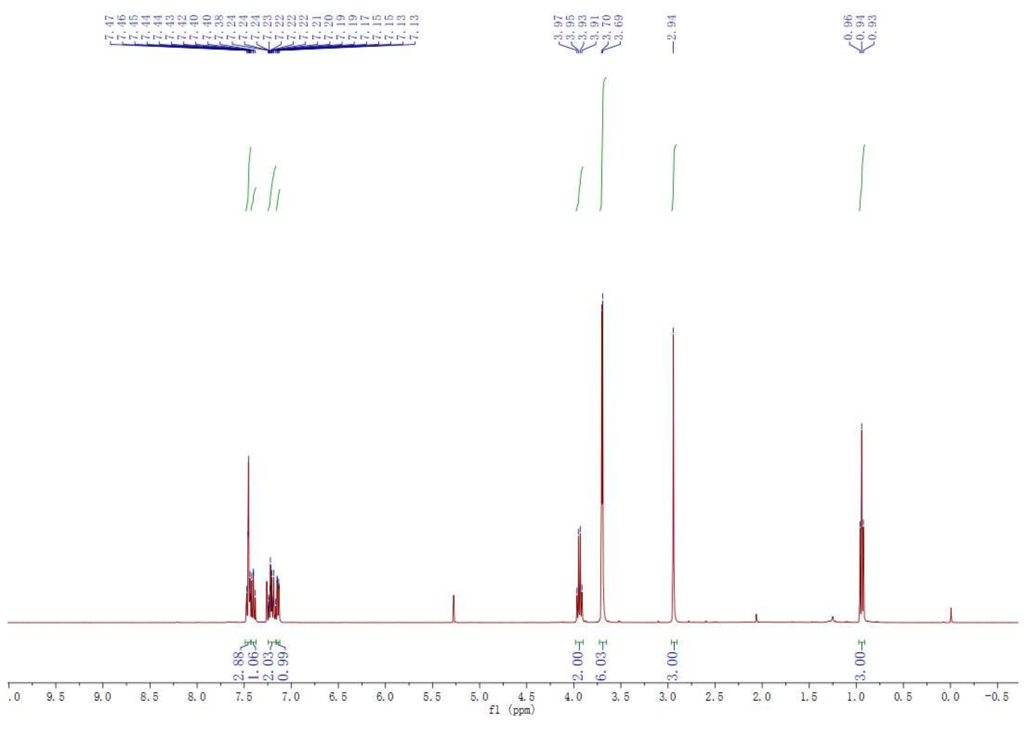

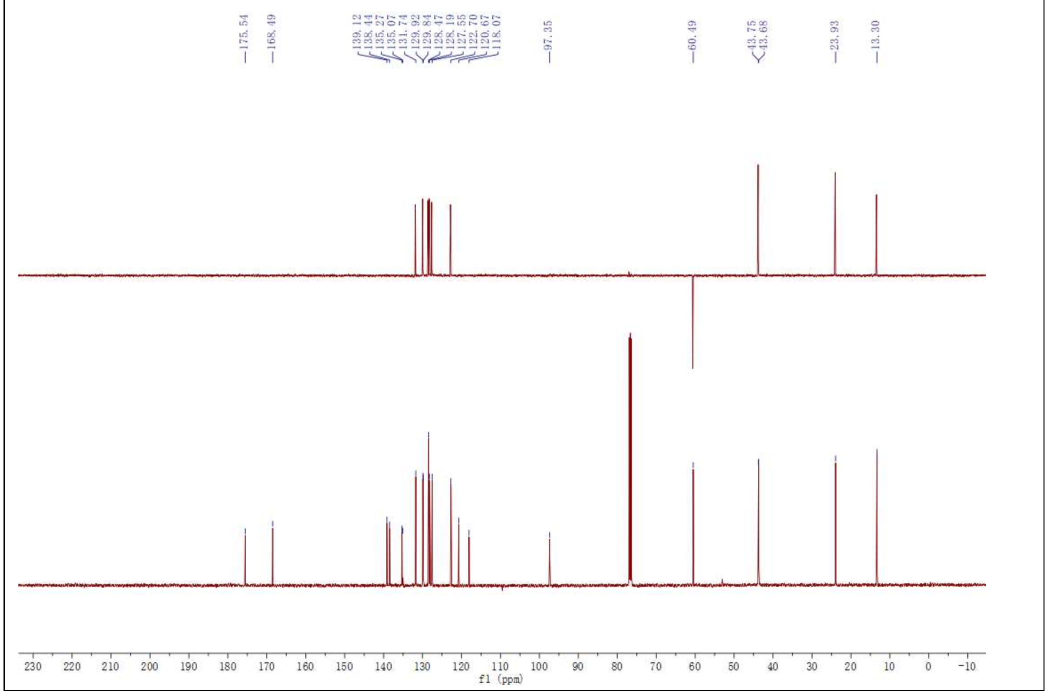

Ethyl 2-(2-chlorophenyl)-3-(dimethyl(oxo)-λ^6-sulfanylidene)-5-methyl-4-oxo- 3,4-dihydronaphthalene-1-carboxylate (**3ai**)

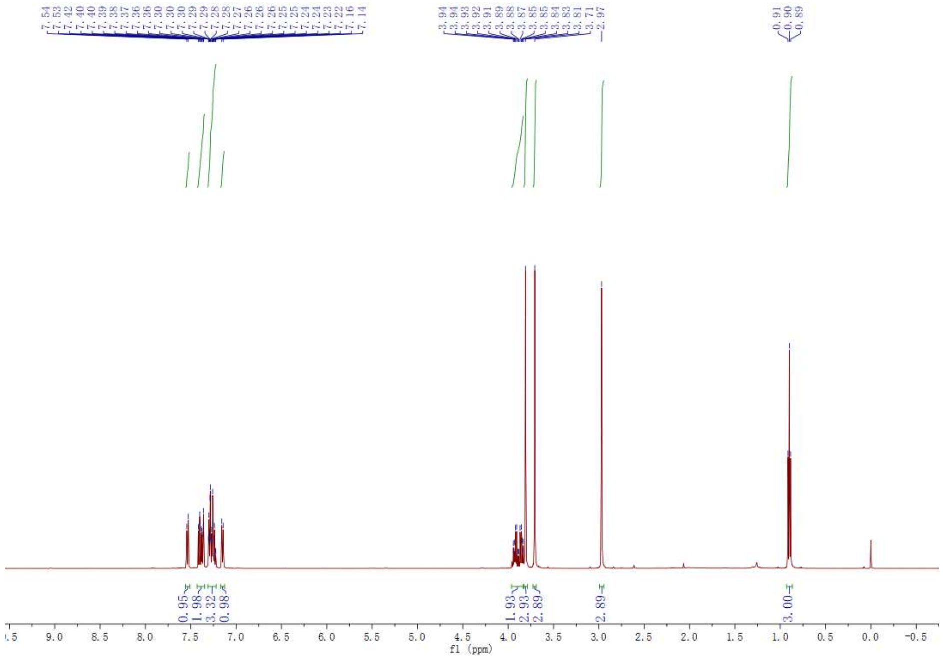

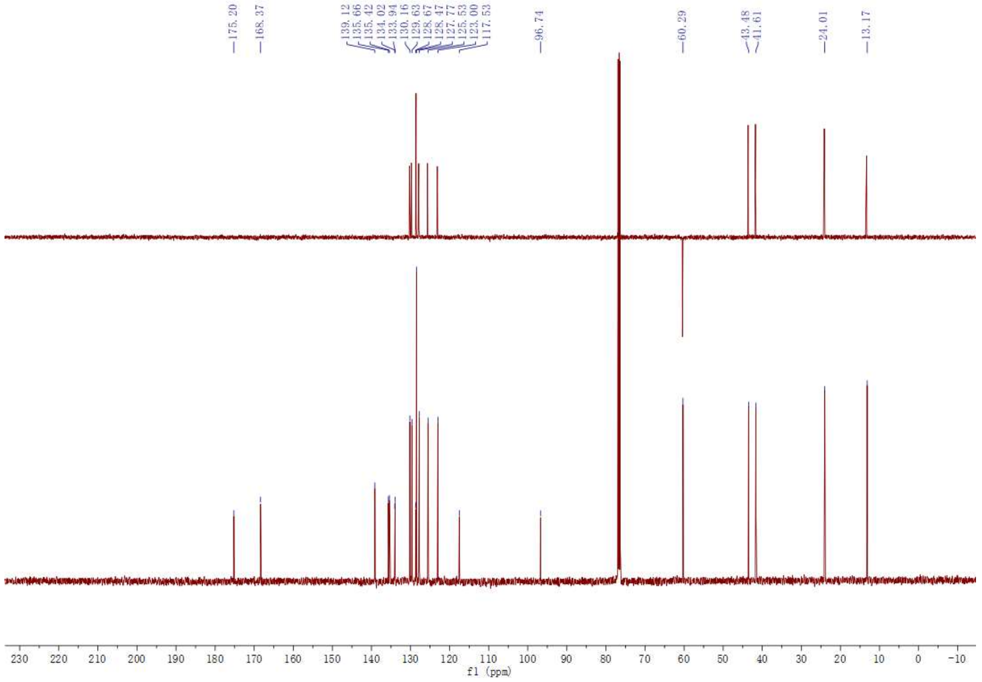

Ethyl 3-(dimethyl(oxo)-λ[6]-sulfanylidene)-2-(2-methoxyphenyl)-5-methyl-4-oxo-3,4-dihydronaphthalene-1-carboxylate(**3aj**)

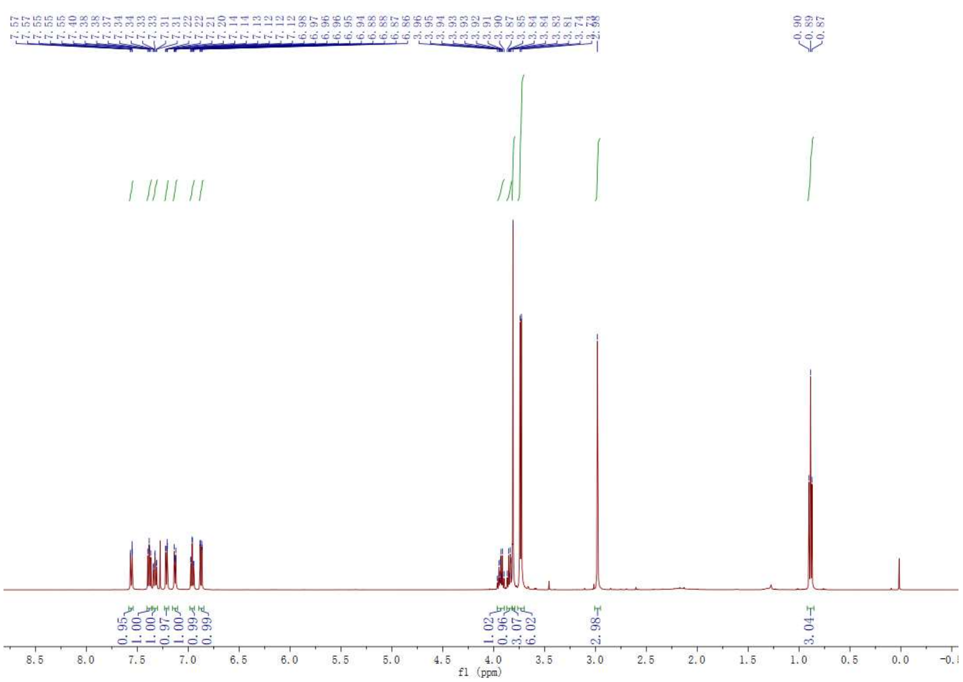

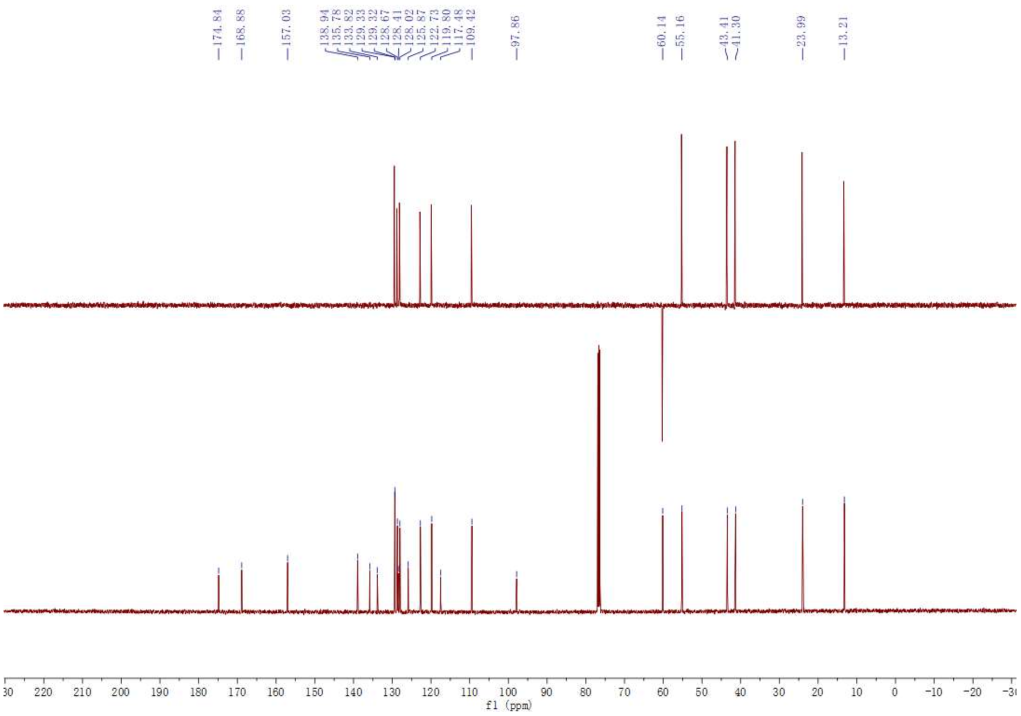

Ethyl 3-(dimethyl(oxo)-λ^6-sulfanylidene)-2,5-dimethyl-4-oxo-3,4-dihydronaphthalene-1-carboxylate(**3ak**)

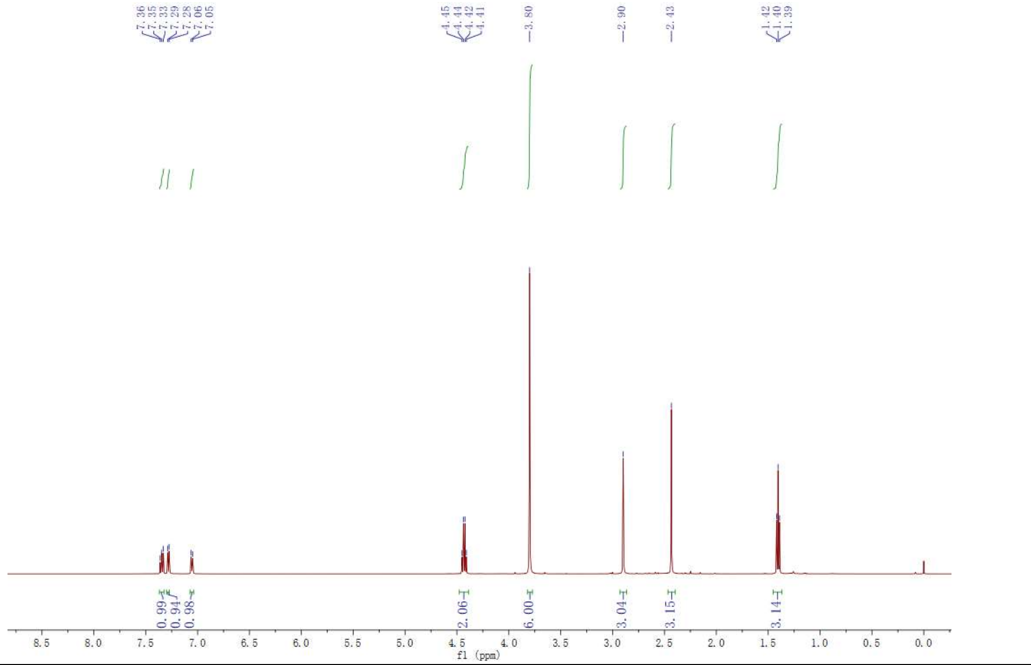

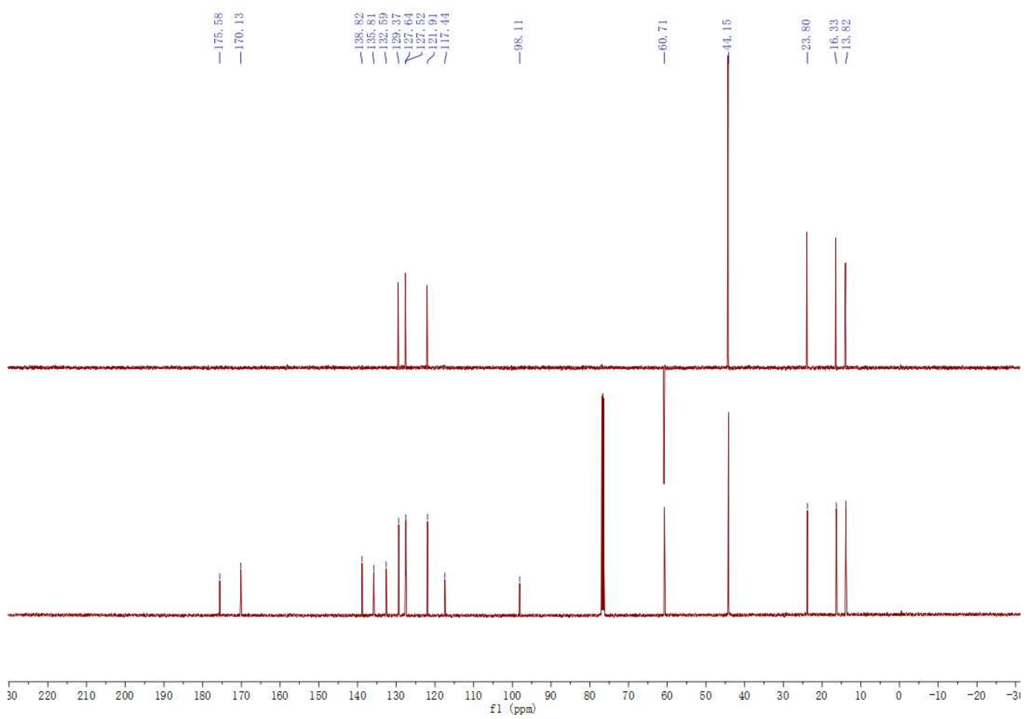

Ethyl 2-cyclopropyl-3-(dimethyl(oxo)-λ^6-sulfanylidene)-5-methyl-4-oxo-3,4-dihydronaphthalene-1-carboxylate(**3al**)

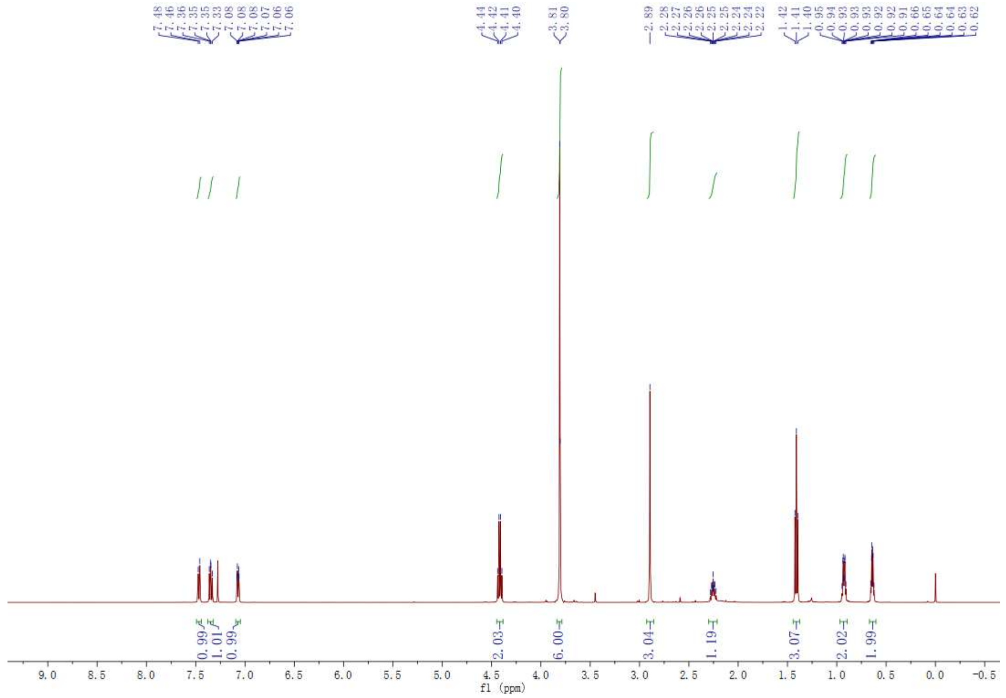

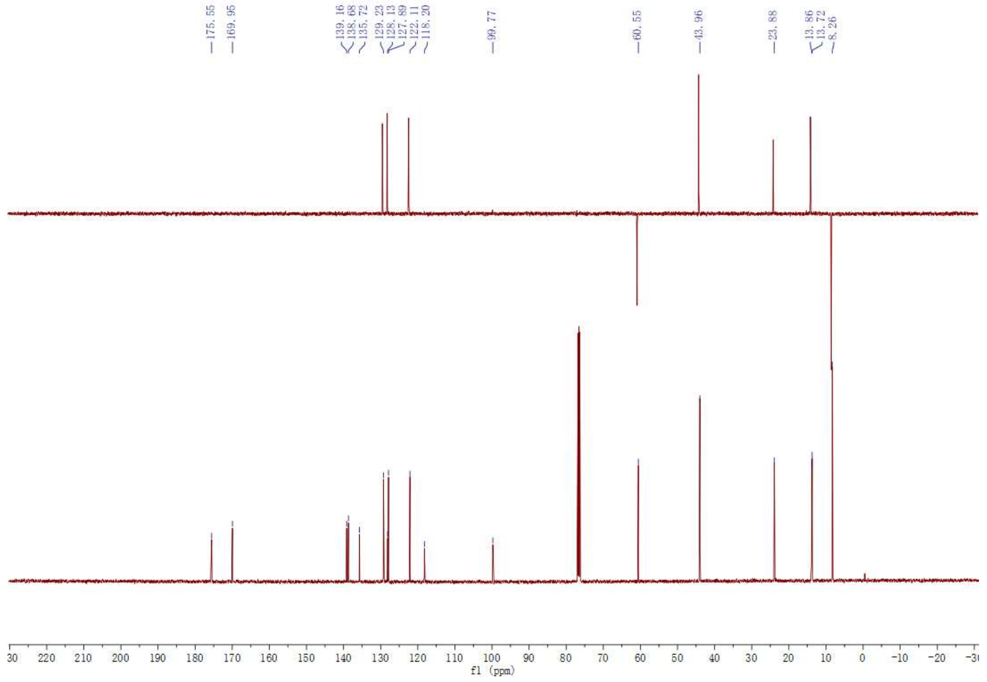

Isopropyl 3-(dimethyl(oxo)-λ^6-sulfanylidene)-2,5-dimethyl-4-oxo-3,4-dihydronaphthalene-1-carboxylate(**3am**)

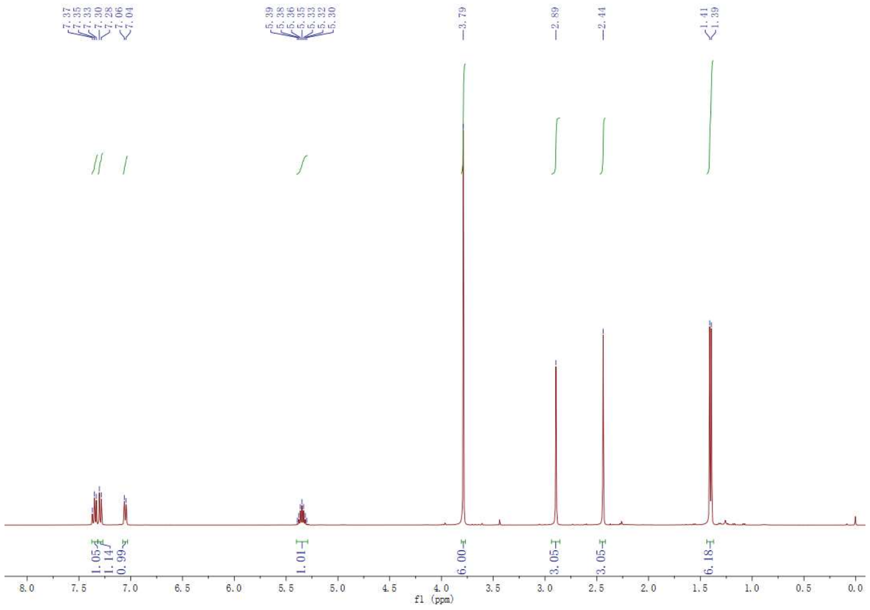

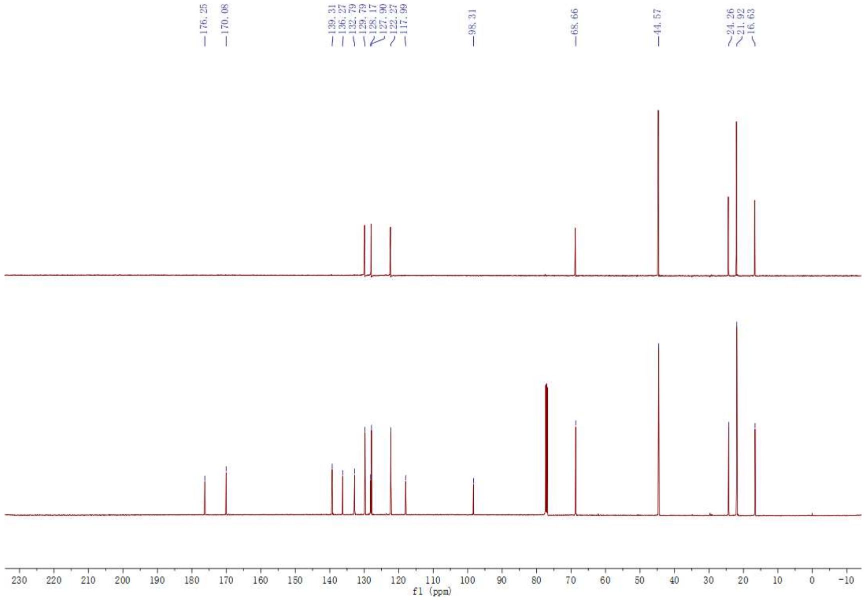

Tert-butyl 3-(dimethyl(oxo)-λ^6-sulfanylidene)-2,5-dimethyl-4-oxo-3,4-dihydronaphthalene-1-carboxylate(**3an**)

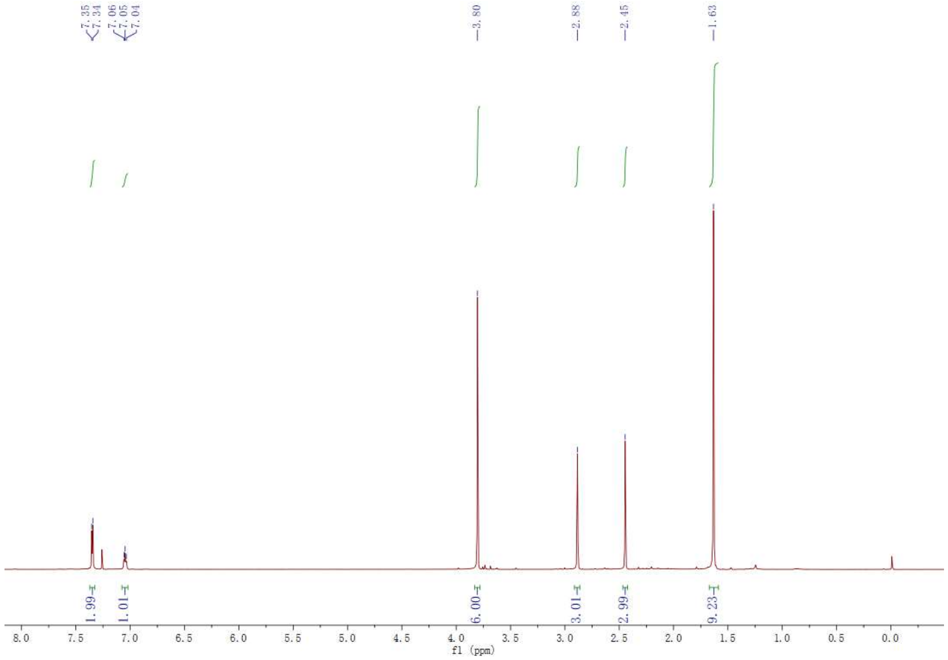

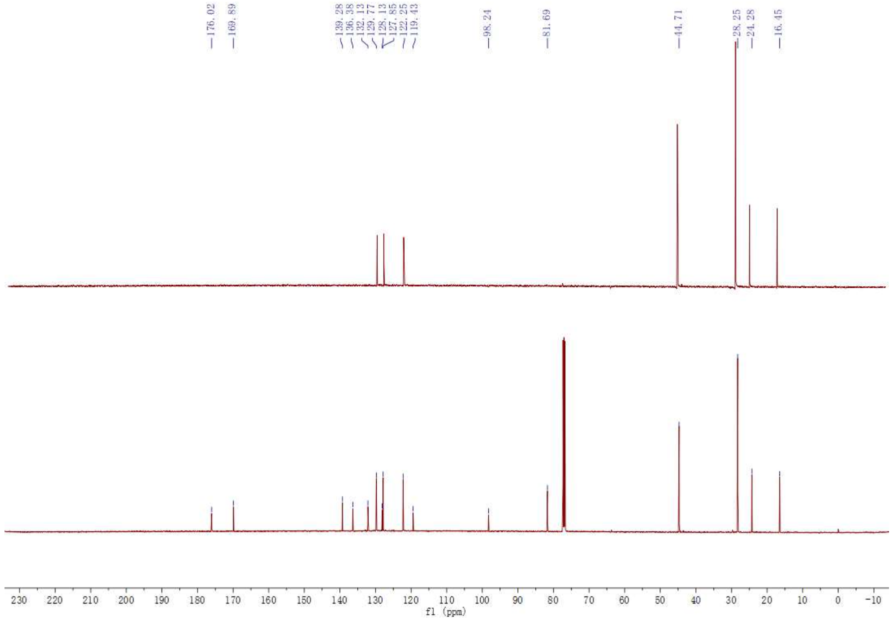

Ethyl 4-hydroxy-3-((4-methoxyphenyl)amino)-2,5-dimethyl-1-naphthoate (**5ak**)

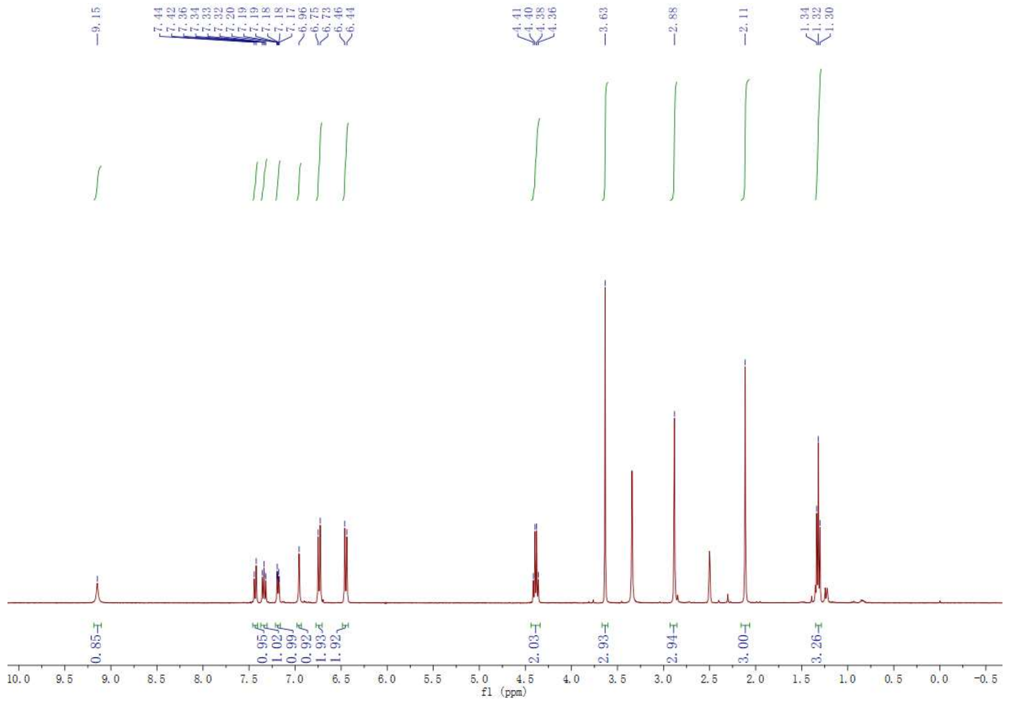

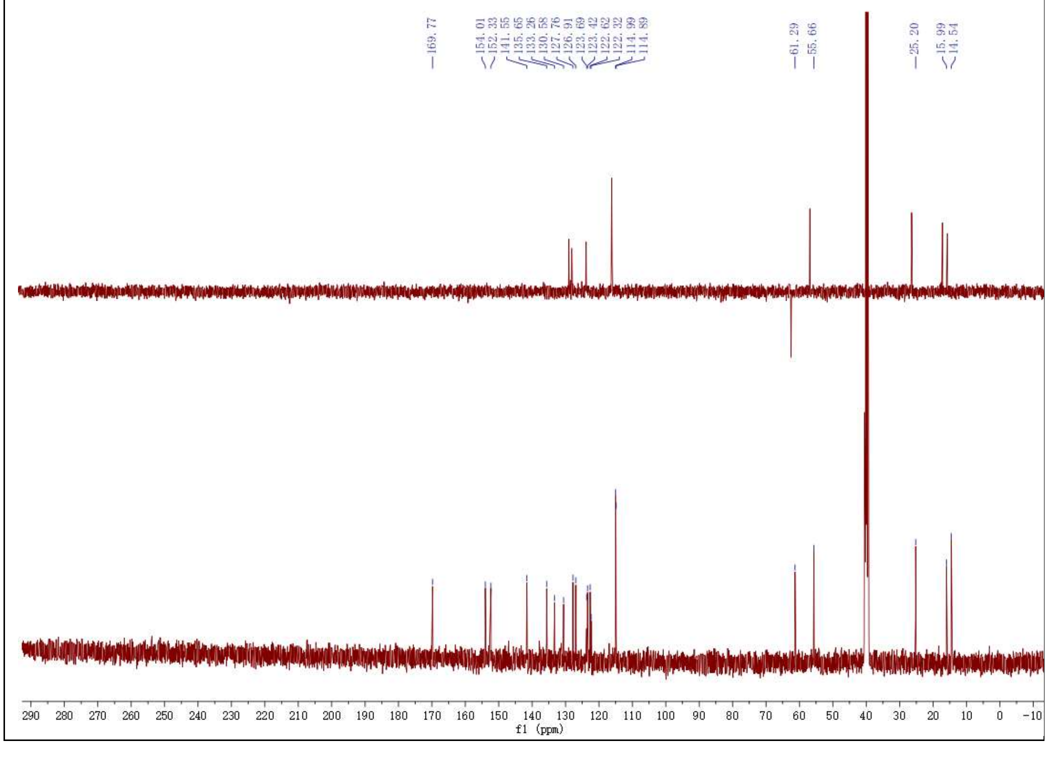

Ethyl 4-hydroxy-2,5-dimethyl-3-(methylsulfinyl)-1-naphthoate (**6ak**)

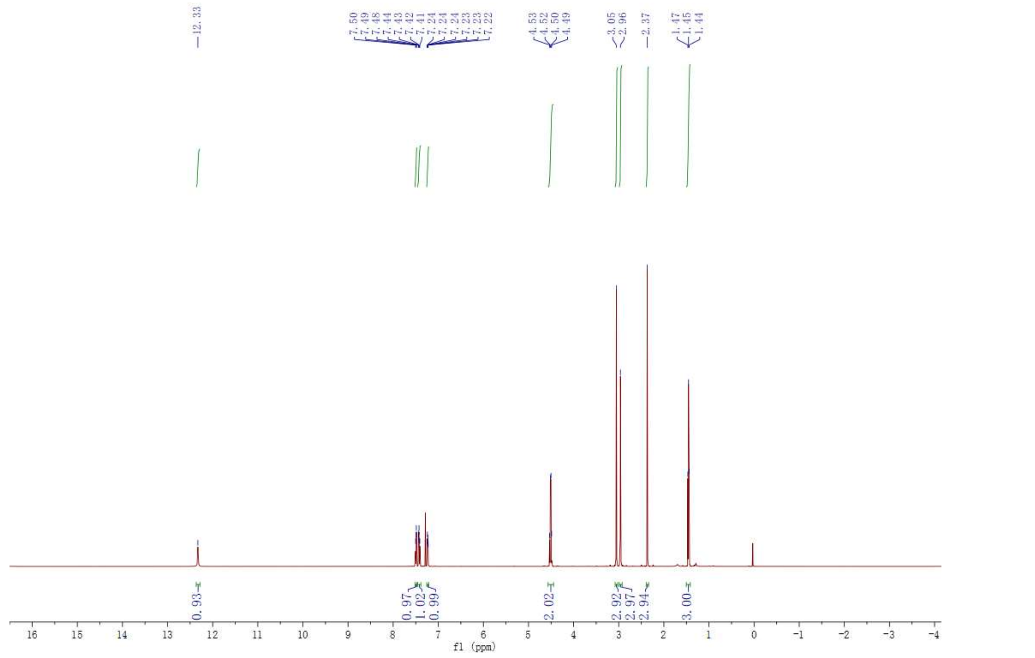

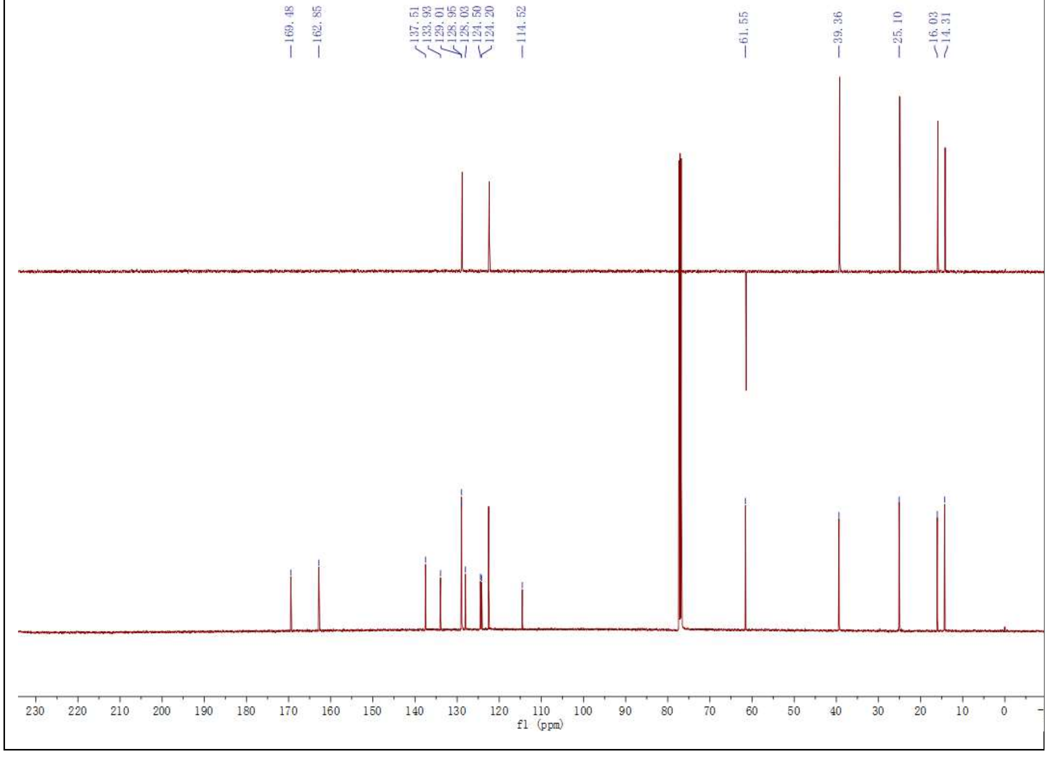

(D)Copies of ^{19}F NMR Spectra for the Products

Ethyl-3-(dimethyl(oxo)-λ^6-sulfanylidene)-4-oxo-2-phenyl-5-(trifluoromethyl)-3,4-dihydronaphthalene-1-carboxylate(**3da**)

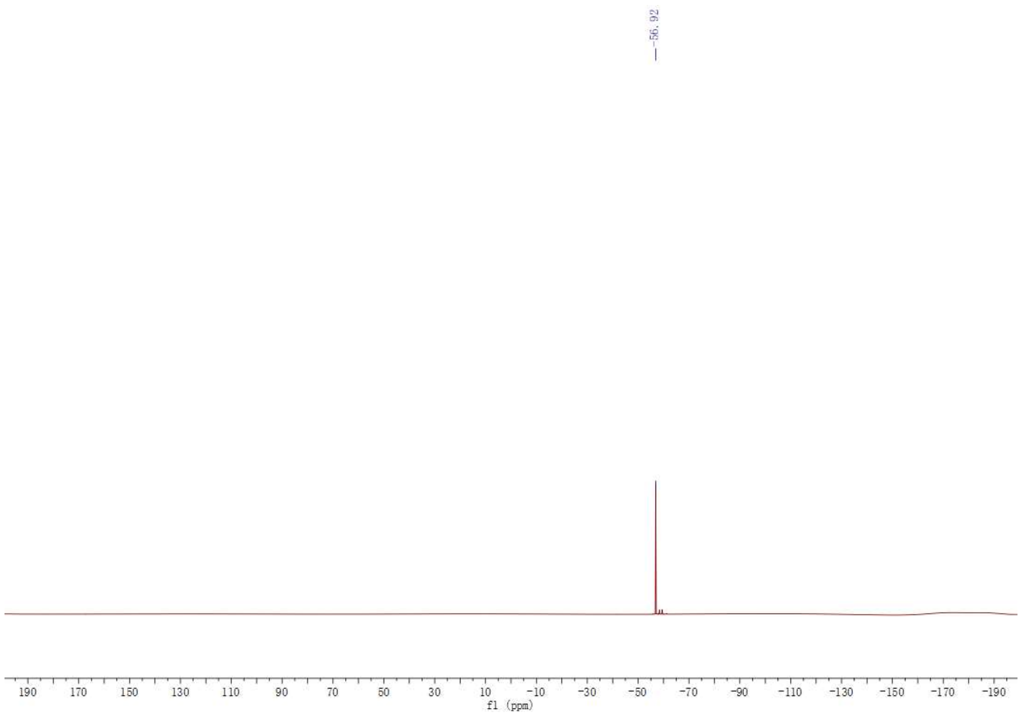

Ethyl-5-chloro-3-(dimethyl(oxo)-λ^6-sulfanylidene)-7-fluoro-4-oxo-2-phenyl-3,4-dihydronaphthalene-1-carboxylate(**3fa**)

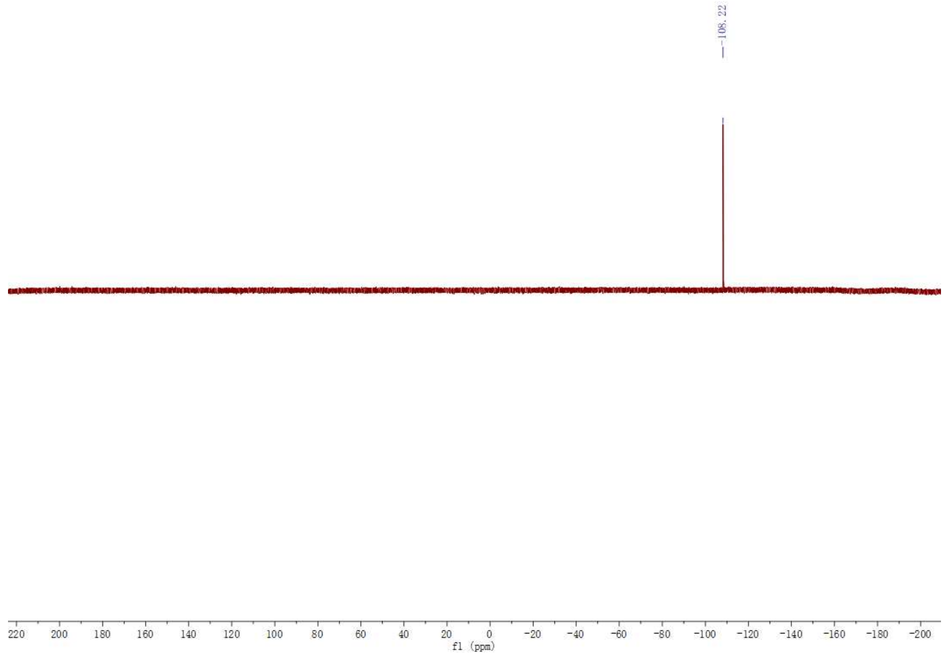

Ethyl-5-chloro-3-(dimethyl(oxo)-λ[6]-sulfanylidene)-4-oxo-2-phenyl-7-(trifluoromethyl)-3,4-dihydronaphthalene-1-carboxylate(**3ia**)

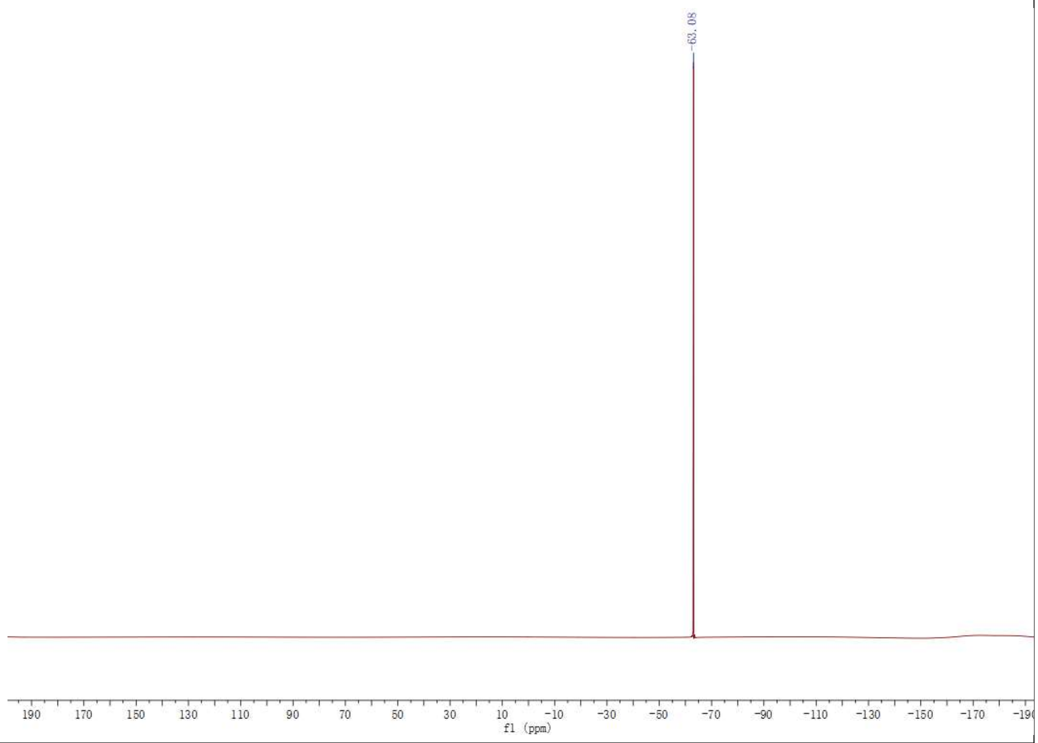

Ethyl-3-(dimethyl(oxo)-λ[6]-sulfanylidene)-2-(4-fluorophenyl)-5-methyl-4-oxo-3,4-dihydronaphthalene-1-carboxylate(**3ab**)

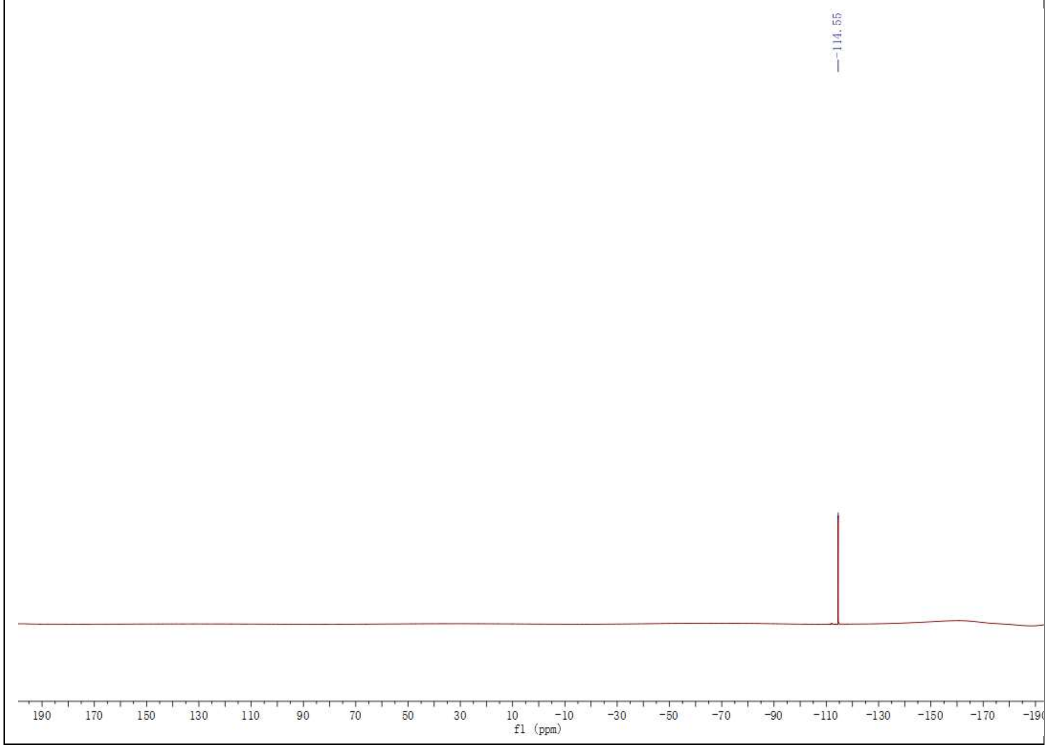

Ethyl-3-(dimethyl(oxo)-λ^6-sulfanylidene)-5-methyl-4-oxo-2-(4-(trifluoromethyl)phenyl)-3,4-dihydronaphthalene-1-carboxylate(**3ae**)

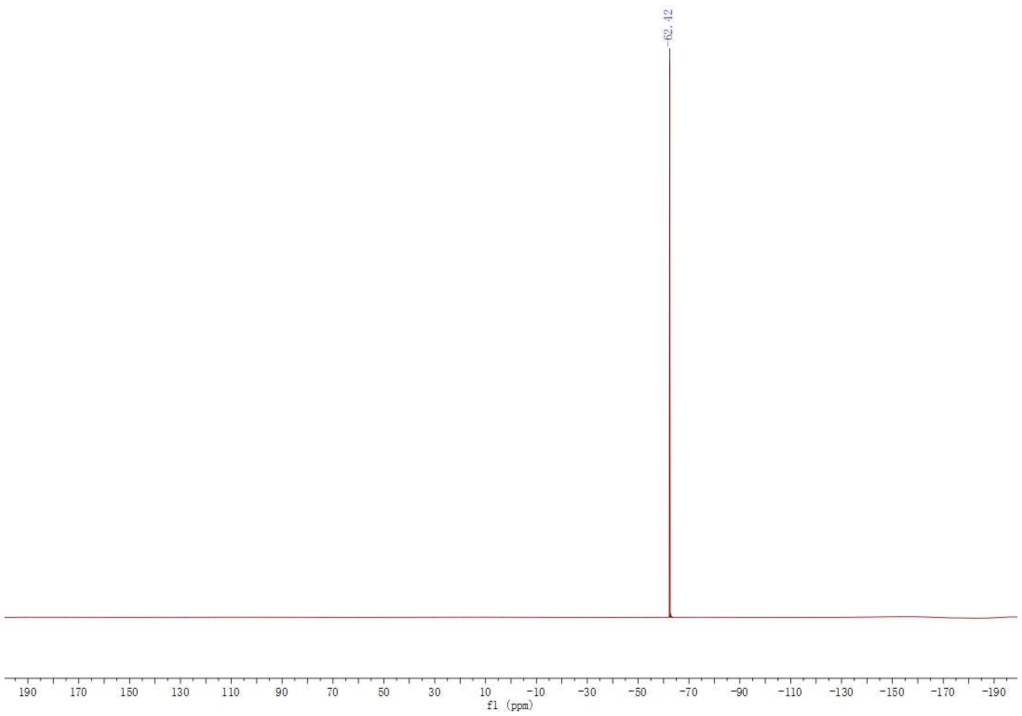

(E)Copies of High Resolution Mass Spectra for the Products

Ethyl 3-(dimethyl(oxo)-λ^6-sulfanylidene)-5-methyl-4-oxo-2-phenyl-3,4-dihydronaphthalene-1-carboxylate (**3aa**)

**Data Filename**	ESIH_20190319_LH_SXH_41.d	**Sample Name**	B6-SXH-1-1
**Sample Type**	Sample	**Position**	P1-F8
**Instrument Name**	Agilent G6520 Q-TOF	**Acq Method**	20160324_MS_ESIH_NEG_1min.m
**Acquired Time**	3/19/2019 19:51:20	**IRM Calibration Status**	Success
**DA Method**	small molecular data analysis method.m	**Comment**	ESIH by ZZY

User Spectra

Fragmentor Voltage	Collision Energy	Ionization Mode
110	0	ESI

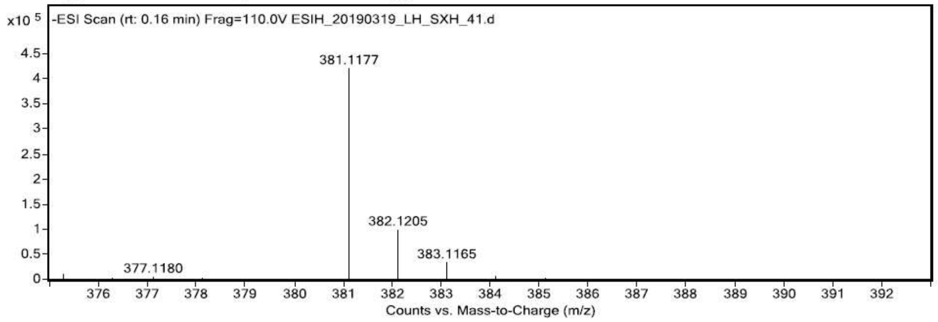

Formula Calculator Results

m/z	Calc m/z	Diff (mDa)	Diff (ppm)	Ion Formula	Ion
381.1177	381.1166	-1.06	-2.77	C22 H21 O4 S	(M-H)-

--- End Of Report ---

Ethyl 5-chloro-3-(dimethyl(oxo)-λ^6-sulfanylidene)-4-oxo-2-phenyl-3,4-dihydronaphthalene-1-carboxylate (**3ba**)

**Data Filename**	ESIH_20190123_LH_SXH_04.d	**Sample Name**	B6-SXH-1-9
**Sample Type**	Sample	**Position**	P2-A2
**Instrument Name**	Agilent G6520 Q-TOF	**Acq Method**	20160322_MS_ESIH_POS_1min.m
**Acquired Time**	1/23/2019 14:56:08	**IRM Calibration Status**	Success
**DA Method**	small molecular data analysis method.m	**Comment**	ESIH by ZZY

User Spectra

Fragmentor Voltage	Collision Energy	Ionization Mode
125	0	ESI

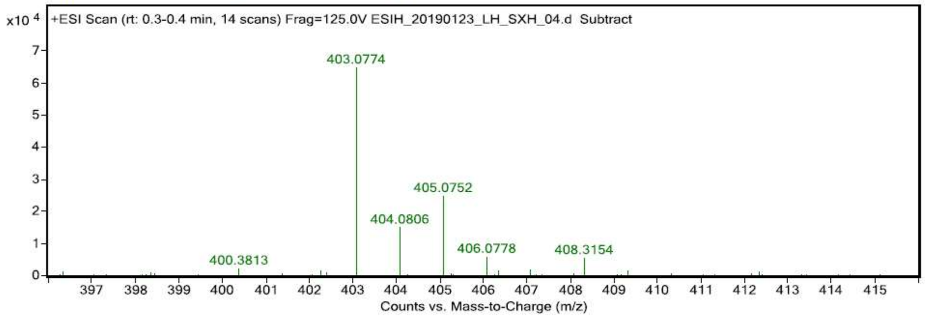

Formula Calculator Results

m/z	Calc m/z	Diff (mDa)	Diff (ppm)	Ion Formula	Ion
403.0774	403.0765	-0.9	-2.23	C21 H20 Cl O4 S	(M+H)+

--- End Of Report ---

Ethyl 5-bromo-3-(dimethyl(oxo)-λ[6]-sulfanylidene)-4-oxo-2-phenyl-3,4-dihydronaphthalene-1-carboxylate(**3ca**)

**Data Filename**	ESIH_20190122_LH_SXH_12.d	**Sample Name**	B6-SXH-3-1
**Sample Type**	Sample	**Position**	P2-B3
**Instrument Name**	Agilent G6520 Q-TOF	**Acq Method**	20160322_MS_ESIH_POS_1min.m
**Acquired Time**	1/22/2019 9:58:04	**IRM Calibration Status**	Success
**DA Method**	small molecular data analysis method.m	**Comment**	ESIH by ZZY

User Spectra

Fragmentor Voltage	Collision Energy	Ionization Mode
125	0	ESI

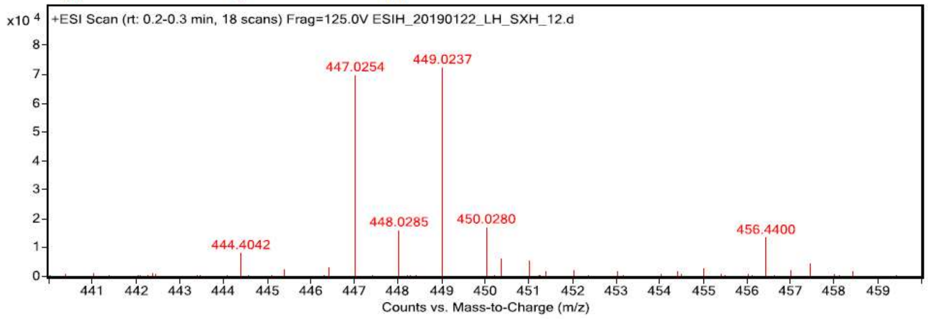

Formula Calculator Results

m/z	Calc m/z	Diff (mDa)	Diff (ppm)	Ion Formula	Ion
447.0254	447.026	0.65	1.46	C21 H20 Br O4 S	(M+H)+

--- End Of Report ---

Ethyl 3-(dimethyl(oxo)-λ[6]-sulfanylidene)-4-oxo-2-phenyl-5-(trifluoromethyl)-3,4-dihydronaphthalene-1-carboxylate(**3da**)

**Data Filename**	ESIH_20190123_LH_SXH_08.d	**Sample Name**	B6-SXH-1-8
**Sample Type**	Sample	**Position**	P2-A6
**Instrument Name**	Agilent G6520 Q-TOF	**Acq Method**	20160322_MS_ESIH_POS_1min.m
**Acquired Time**	1/23/2019 15:03:38	**IRM Calibration Status**	Success
**DA Method**	small molecular data analysis method.m	**Comment**	ESIH by ZZY

User Spectra

Fragmentor Voltage	Collision Energy	Ionization Mode
125	0	ESI

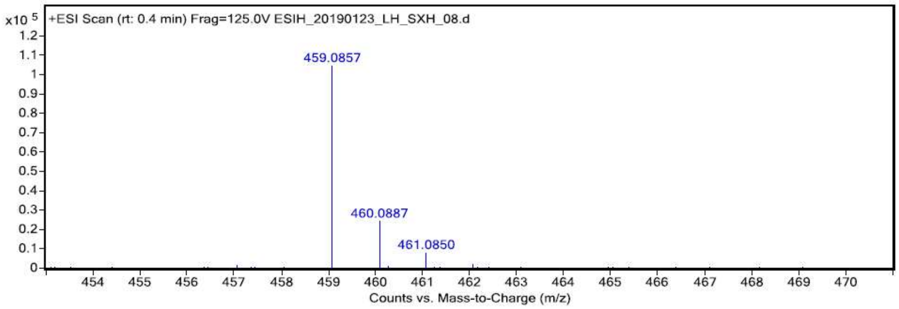

Formula Calculator Results

m/z	Calc m/z	Diff (mDa)	Diff (ppm)	Ion Formula	Ion
459.0857	459.0848	-0.86	-1.88	C22 H19 F3 Na O4 S	(M+Na)+

--- End Of Report ---

Ethyl 5-chloro-3-(dimethyl(oxo)-λ[6]-sulfanylidene)-7-methyl-4-oxo-2-phenyl-3,4-di

hydronaphthalene-1-carboxylate(**3ea**)

**Data Filename**	ESIH_20190228_LH_SXH_49.d	**Sample Name**	B6-SXH-7-3
**Sample Type**	Sample	**Position**	P1-F4
**Instrument Name**	Agilent G6520 Q-TOF	**Acq Method**	20160322_MS_ESIH_POS_1min.m
**Acquired Time**	2/28/2019 16:42:13	**IRM Calibration Status**	Success
**DA Method**	small molecular data analysis method.m	**Comment**	ESIH by ZZY

User Spectra

Fragmentor Voltage	Collision Energy	Ionization Mode
200	0	ESI

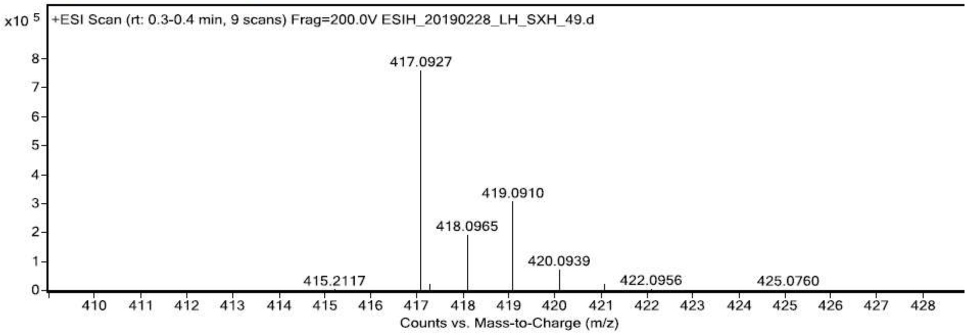

Formula Calculator Results

m/z	Calc m/z	Diff (mDa)	Diff (ppm)	Ion Formula	Ion
417.0927	417.0922	-0.51	-1.22	C22 H22 Cl O4 S	(M+H)+

--- End Of Report ---

Ethyl 5-chloro-3-(dimethyl(oxo)-λ^6-sulfanylidene)-7-fluoro-4-oxo-2-phenyl-3,4-dihydronaphthalene-1-carboxylate(**3fa**)

**Data Filename**	ESIH_20190122_LH_SXH_11.d	**Sample Name**	B6-SXH-3-5
**Sample Type**	Sample	**Position**	P2-B2
**Instrument Name**	Agilent G6520 Q-TOF	**Acq Method**	20160322_MS_ESIH_POS_1min.m
**Acquired Time**	1/22/2019 9:56:10	**IRM Calibration Status**	Success
**DA Method**	small molecular data analysis method.m	**Comment**	ESIH by ZZY

User Spectra

Fragmentor Voltage	Collision Energy	Ionization Mode
125	0	ESI

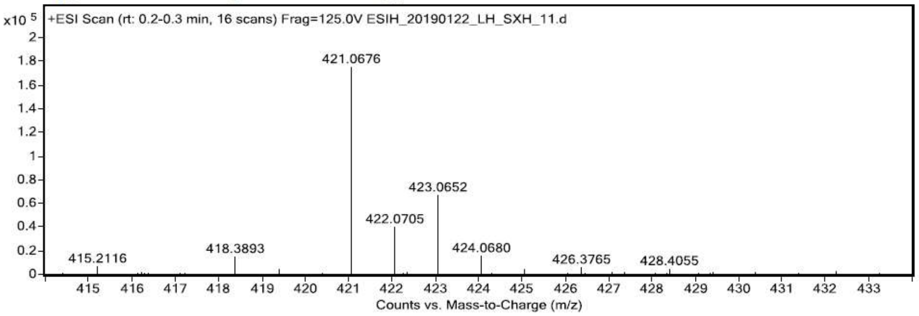

Formula Calculator Results

m/z	Calc m/z	Diff (mDa)	Diff (ppm)	Ion Formula	Ion
421.0676	421.0671	-0.46	-1.1	C21 H19 Cl F O4 S	(M+H)+

--- End Of Report ---

Ethyl 5,7-dichloro-3-(dimethyl(oxo)-λ^6-sulfanylidene)-4-oxo-2-phenyl-3,4-dihy-dro

naphthalene-1-carboxylate(**3ga**)

**Data Filename**	ESIH_20190122_LH_SXH_14.d	**Sample Name**	B6-SXH-3-4
**Sample Type**	Sample	**Position**	P2-B5
**Instrument Name**	Agilent G6520 Q-TOF	**Acq Method**	20160322_MS_ESIH_POS_1min.m
**Acquired Time**	1/22/2019 10:01:49	**IRM Calibration Status**	Success
**DA Method**	small molecular data analysis method.m	**Comment**	ESIH by ZZY

User Spectra

Fragmentor Voltage	Collision Energy	Ionization Mode
125	0	ESI

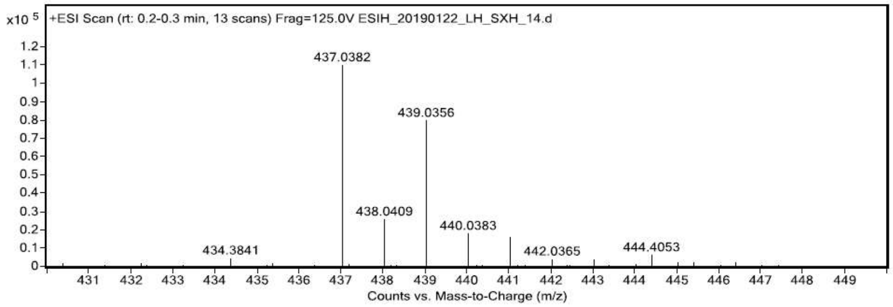

Formula Calculator Results

m/z	Calc m/z	Diff (mDa)	Diff (ppm)	Ion Formula	Ion
437.0382	437.0376	-0.64	-1.45	C21 H19 Cl2 O4 S	(M+H)+

--- End Of Report ---

Ethyl 5-chloro-3-(dimethyl(oxo)-λ^6-sulfanylidene)-7-methoxy-4-oxo-2-phenyl- 3,4-dihydronaphthalene-1-carboxylate(**3ha**)

**Data Filename**	ESIH_20190228_LH_SXH_47.d	**Sample Name**	B6-SXH-6-3
**Sample Type**	Sample	**Position**	P1-F2
**Instrument Name**	Agilent G6520 Q-TOF	**Acq Method**	20160322_MS_ESIH_POS_1min.m
**Acquired Time**	2/28/2019 16:38:33	**IRM Calibration Status**	Success
**DA Method**	small molecular data analysis method.m	**Comment**	ESIH by ZZY

User Spectra

Fragmentor Voltage	Collision Energy	Ionization Mode
200	0	ESI

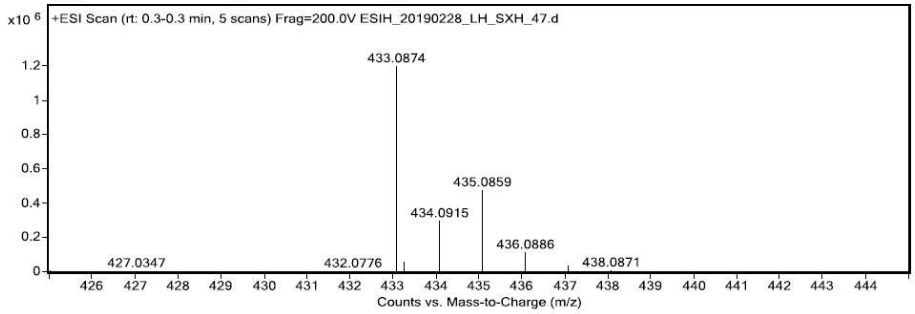

Formula Calculator Results

m/z	Calc m/z	Diff (mDa)	Diff (ppm)	Ion Formula	Ion
433.0874	433.0871	-0.29	-0.67	C22 H22 Cl O5 S	(M+H)+

--- End Of Report ---

Ethyl 5-chloro-3-(dimethyl(oxo)-λ^6-sulfanylidene)-4-oxo-2-phenyl-7-(trifluoro

methyl)-3,4-dihydronaphthalene-1-carboxylate(**3ia**)

**Data Filename**	ESIH_20190228_LH_SXH_48.d	**Sample Name**	B6-SXH-7-2
**Sample Type**	Sample	**Position**	P1-F3
**Instrument Name**	Agilent G6520 Q-TOF	**Acq Method**	20160322_MS_ESIH_POS_1min.m
**Acquired Time**	2/28/2019 16:40:22	**IRM Calibration Status**	Success
**DA Method**	small molecular data analysis method.m	**Comment**	ESIH by ZZY

User Spectra

Fragmentor Voltage	Collision Energy	Ionization Mode
200	0	ESI

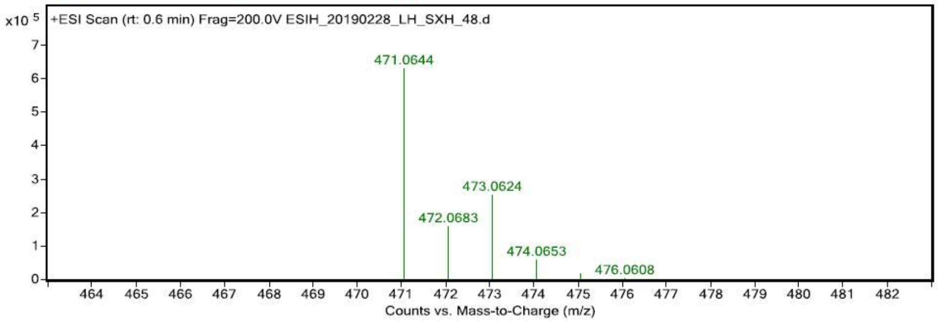

Formula Calculator Results

m/z	Calc m/z	Diff (mDa)	Diff (ppm)	Ion Formula	Ion
471.0644	471.0639	-0.53	-1.12	C22 H19 Cl F3 O4 S	(M+H)+

--- End Of Report ---

Ethyl 6-bromo-5-chloro-3-(dimethyl(oxo)-λ^6-sulfanylidene)-4-oxo-2-phenyl-3,4-dihydronaphthalene-1-carboxylate (**3ja**)

**Data Filename**	ESIH_20190416_LH_SXH_05.d	**Sample Name**	B6-8-2
**Sample Type**	Sample	**Position**	P1-A5
**Instrument Name**	Agilent G6520 Q-TOF	**Acq Method**	20160322_MS_ESIH_POS_1min.m
**Acquired Time**	4/16/2019 10:52:06	**IRM Calibration Status**	Success
**DA Method**	small molecular data analysis method.m	**Comment**	ESIH by ZZY

User Spectra

Fragmentor Voltage	Collision Energy	Ionization Mode
135	0	ESI

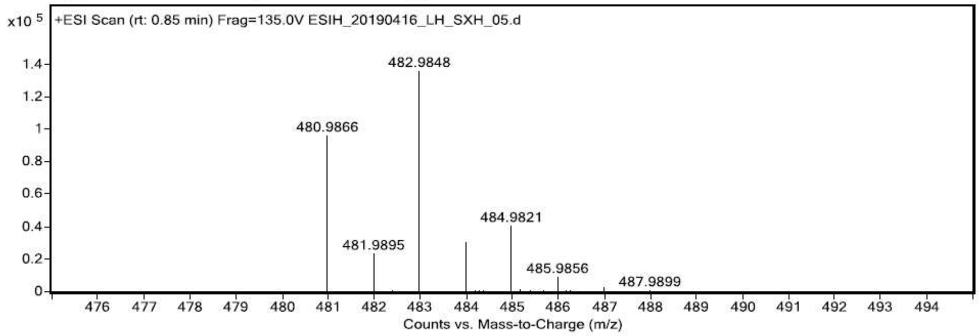

Formula Calculator Results

m/z	Calc m/z	Diff (mDa)	Diff (ppm)	Ion Formula	Ion
480.9866	480.987	0.47	0.98	C21 H19 Br Cl O4 S	(M+H)+

--- End Of Report ---

Ethyl 5-chloro-3-(dimethyl(oxo)-λ^6-sulfanylidene)-6-methyl-4-oxo-2-phenyl-3,4-di

hydronaphthalene-1-carboxylate (**3ka**)

**Data Filename**	ESIH_20190416_LH_SXH_04.d	**Sample Name**	B6-8-1
**Sample Type**	Sample	**Position**	P1-A4
**Instrument Name**	Agilent G6520 Q-TOF	**Acq Method**	20160322_MS_ESIH_POS_1min.m
**Acquired Time**	4/16/2019 10:50:16	**IRM Calibration Status**	Success
**DA Method**	small molecular data analysis method.m	**Comment**	ESIH by ZZY

User Spectra

Fragmentor Voltage	Collision Energy	Ionization Mode
135	0	ESI

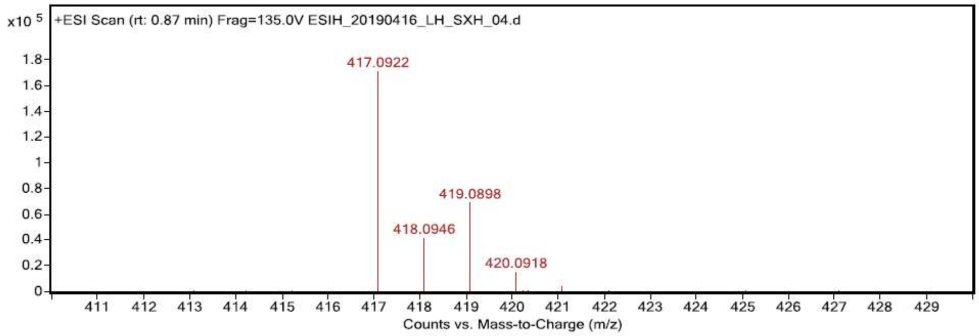

Formula Calculator Results

m/z	Calc m/z	Diff (mDa)	Diff (ppm)	Ion Formula	Ion
417.0922	417.0922	-0.04	-0.09	C22 H22 Cl O4 S	(M+H)+

--- End Of Report ---

Ethyl 5-chloro-3-(dimethyl(oxo)-λ^6-sulfanylidene)-8-methoxy-4-oxo-2-phenyl-3,4-dihydronaphthalene-1-carboxylate (**3la**)

**Data Filename**	ESIH_20190416_LH_SXH_06.d	**Sample Name**	B6-8-1
**Sample Type**	Sample	**Position**	P1-A6
**Instrument Name**	Agilent G6520 Q-TOF	**Acq Method**	20160322_MS_ESIH_POS_1min.m
**Acquired Time**	4/16/2019 10:53:56	**IRM Calibration Status**	Success
**DA Method**	small molecular data analysis method.m	**Comment**	ESIH by ZZY

User Spectra

Fragmentor Voltage	Collision Energy	Ionization Mode
135	0	ESI

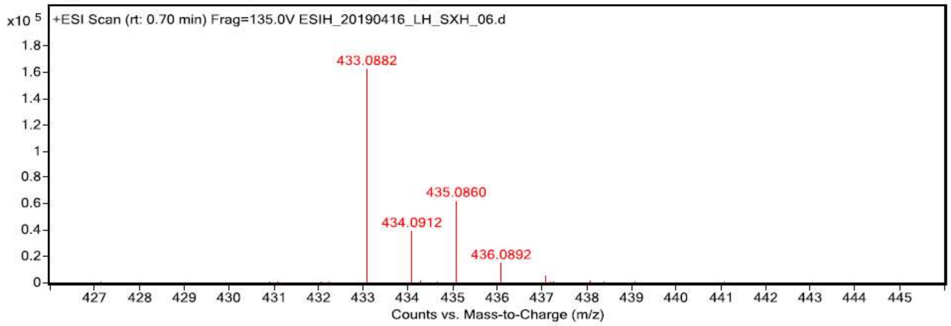

Formula Calculator Results

m/z	Calc m/z	Diff (mDa)	Diff (ppm)	Ion Formula	Ion
433.0882	433.0871	-1.14	-2.64	C22 H22 Cl O5 S	(M+H)+

--- End Of Report ---

Ethyl 3-(dimethyl(oxo)-λ^6-sulfanylidene)-5-(1-ethoxy-1,3-dioxo-3-phenylpro pan-2-yl)-4-oxo-2-phenyl-3,4-dihydronaphthalene-1-carboxylate(**3ma**)

**Data Filename**	ESIH_20190306_LH_SXH_01.d	**Sample Name**	B6-SXH-Ph
**Sample Type**	Sample	**Position**	P1-C4
**Instrument Name**	Agilent G6520 Q-TOF	**Acq Method**	20160322_MS_ESIH_POS_1min.m
**Acquired Time**	3/6/2019 17:57:28	**IRM Calibration Status**	Success
**DA Method**	small molecular data analysis method.m	**Comment**	ESIH by ZZY

User Spectra

Fragmentor Voltage	Collision Energy	Ionization Mode
135	0	ESI

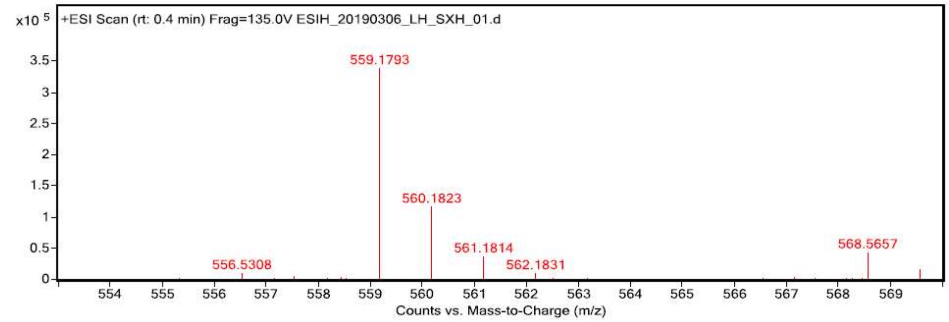

Formula Calculator Results

m/z	Calc m/z	Diff (mDa)	Diff (ppm)	Ion Formula	Ion
559.1793	559.1785	-0.83	-1.49	C32 H31 O7 S	(M+H)+

--- End Of Report ---

Ethyl 3-(dimethyl(oxo)-λ^6-sulfanylidene)-5-(1-ethoxy-1,3-dioxo-3-phenylpropan -2-yl)-7-methoxy-4-oxo-2-phenyl-3,4-dihydronaphthalene-1-carboxylate (**3na**)

**Data Filename**	ESIH_20190416_LH_SXH_02.d	**Sample Name**	B6-9-4
**Sample Type**	Sample	**Position**	P1-A2
**Instrument Name**	Agilent G6520 Q-TOF	**Acq Method**	20160322_MS_ESIH_POS_1min.m
**Acquired Time**	4/16/2019 10:46:37	**IRM Calibration Status**	Success
**DA Method**	small molecular data analysis method.m	**Comment**	ESIH by ZZY

User Spectra

Fragmentor Voltage	Collision Energy	Ionization Mode
135	0	ESI

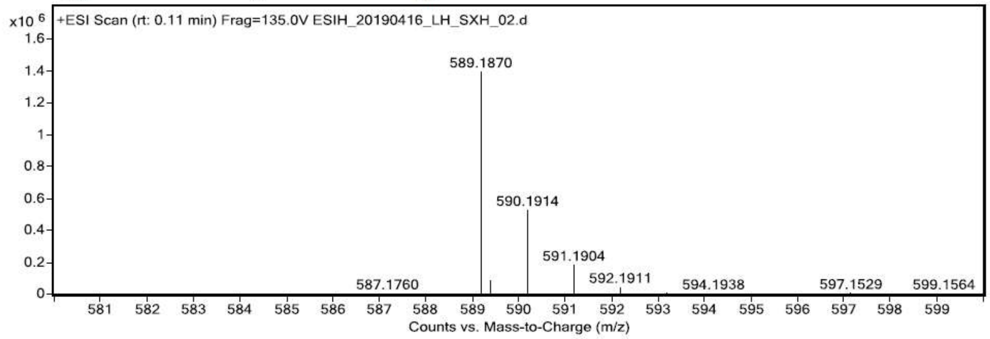

Formula Calculator Results

m/z	Calc m/z	Diff (mDa)	Diff (ppm)	Ion Formula	Ion
589.187	589.1891	2.08	3.54	C33 H33 O8 S	(M+H)+

--- End Of Report ---

Ethyl 7-(tert-butyl)-3-(dimethyl(oxo)-l6-sulfanylidene)-5-(1-ethoxy-1,3-dioxo-3-phenylpropan-2-yl)-4-oxo-2-phenyl-3,4-dihydronaphthalene-1-carboxylate (**3oa**)

**Data Filename**	ESIH_20190416_LH_SXH_01.d	**Sample Name**	B6-9-3
**Sample Type**	Sample	**Position**	P1-A1
**Instrument Name**	Agilent G6520 Q-TOF	**Acq Method**	20160322_MS_ESIH_POS_1min.m
**Acquired Time**	4/16/2019 10:44:42	**IRM Calibration Status**	Success
**DA Method**	small molecular data analysis method.m	**Comment**	ESIH by ZZY

User Spectra

Fragmentor Voltage	Collision Energy	Ionization Mode
135	0	ESI

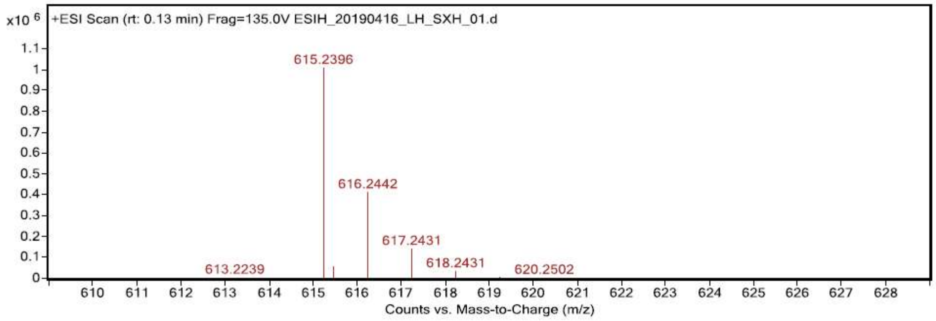

Formula Calculator Results

m/z	Calc m/z	Diff (mDa)	Diff (ppm)	Ion Formula	Ion
615.2396	615.2411	1.49	2.43	C36 H39 O7 S	(M+H)+

--- End Of Report ---

Ethyl 7-bromo-3-(dimethyl(oxo)-l6-sulfanylidene)-5-(1-ethoxy-1,3-dioxo-3-phenylpropan-2-yl)-4-oxo-2-phenyl-3,4-dihydronaphthalene-1-carboxylate (**3pa**)

**Data Filename**	ESIH_20190416_LH_SXH_03.d	**Sample Name**	B6-9-8
**Sample Type**	Sample	**Position**	P1-A3
**Instrument Name**	Agilent G6520 Q-TOF	**Acq Method**	20160322_MS_ESIH_POS_1min.m
**Acquired Time**	4/16/2019 10:48:27	**IRM Calibration Status**	Success
**DA Method**	small molecular data analysis method.m	**Comment**	ESIH by ZZY

User Spectra

Fragmentor Voltage	Collision Energy	Ionization Mode
135	0	ESI

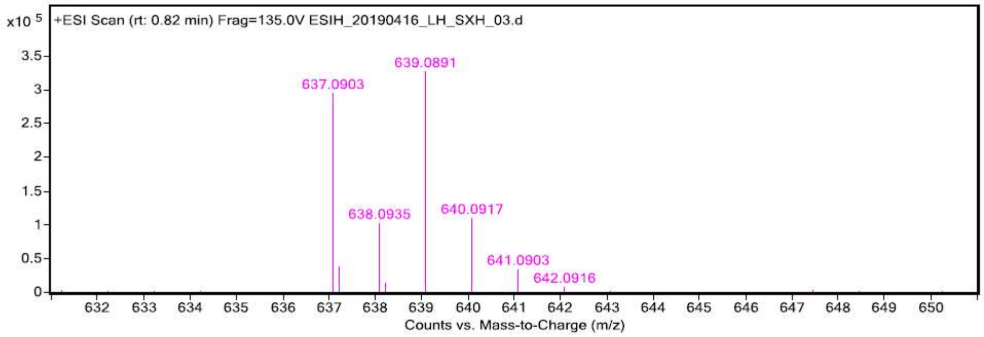

Formula Calculator Results

m/z	Calc m/z	Diff (mDa)	Diff (ppm)	Ion Formula	Ion
637.0903	637.089	-1.29	-2.02	C32 H30 Br O7 S	(M+H)+

--- End Of Report ---

Ethyl 3-(dimethyl(oxo)-λ^6-sulfanylidene)-2-(4-fluorophenyl)-5-methyl-4-oxo-3,4-dihydronaphthalene-1-carboxylate(**3ab**)

**Data Filename**	ESIH_20190122_LH_SXH_P-13.d	**Sample Name**	B6-SXH-2-2
**Sample Type**	Sample	**Position**	P1-B3
**Instrument Name**	Agilent G6520 Q-TOF	**Acq Method**	20160322_MS_ESIH_POS_1min.m
**Acquired Time**	1/22/2019 15:07:39	**IRM Calibration Status**	Success
**DA Method**	small molecular data analysis method.m	**Comment**	ESIH by ZZY

User Spectra

Fragmentor Voltage	Collision Energy	Ionization Mode
125	0	ESI

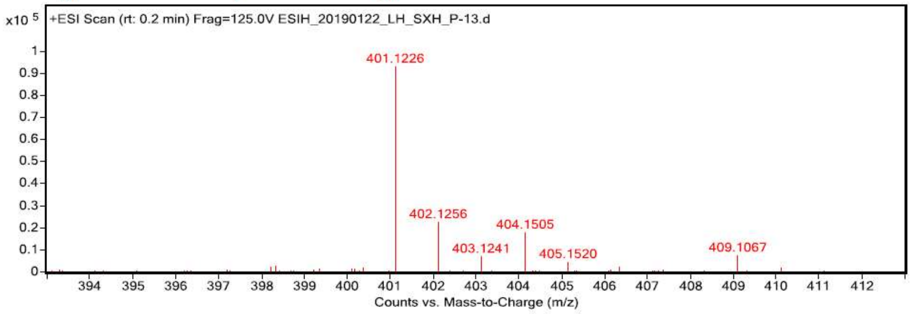

--- End Of Report ---

Ethyl 2-(4-chlorophenyl)-3-(dimethyl(oxo)-λ^6-sulfanylidene)-5-methyl-4-oxo-3,4-dihydronaphthalene-1-carboxylate(**3ac**)

**Data Filename**	ESIH_20190123_LH_SXH_07.d	**Sample Name**	B6-SXH-1-1
**Sample Type**	Sample	**Position**	P2-A5
**Instrument Name**	Agilent G6520 Q-TOF	**Acq Method**	20160322_MS_ESIH_POS_1min.m
**Acquired Time**	1/23/2019 15:01:47	**IRM Calibration Status**	Success
**DA Method**	small molecular data analysis method.m	**Comment**	ESIH by ZZY

User Spectra

Fragmentor Voltage	Collision Energy	Ionization Mode
125	0	ESI

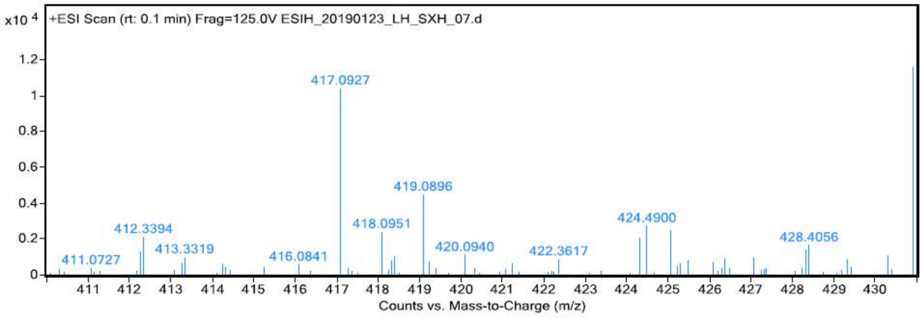

Formula Calculator Results

m/z	Calc m/z	Diff (mDa)	Diff (ppm)	Ion Formula	Ion
417.0927	417.0922	-0.53	-1.27	C22 H22 Cl O4 S	(M+H)+

Ethyl 2-(4-bromophenyl)-3-(dimethyl(oxo)-λ^6-sulfanylidene)-5-methyl-4-oxo-3,4-dihydronaphthalene-1-carboxylate(**3ad**)

**Data Filename**	ESIH_20190123_LH_SXH_N-10.d	**Sample Name**	B6-SXH-2-3
**Sample Type**	Sample	**Position**	P2-A8
**Instrument Name**	Agilent G6520 Q-TOF	**Acq Method**	20160324_MS_ESIH_NEG_1min.m
**Acquired Time**	1/23/2019 16:14:13	**IRM Calibration Status**	Success
**DA Method**	small molecular data analysis method.m	**Comment**	ESIH by ZZY

User Spectra

Fragmentor Voltage	Collision Energy	Ionization Mode
135	0	ESI

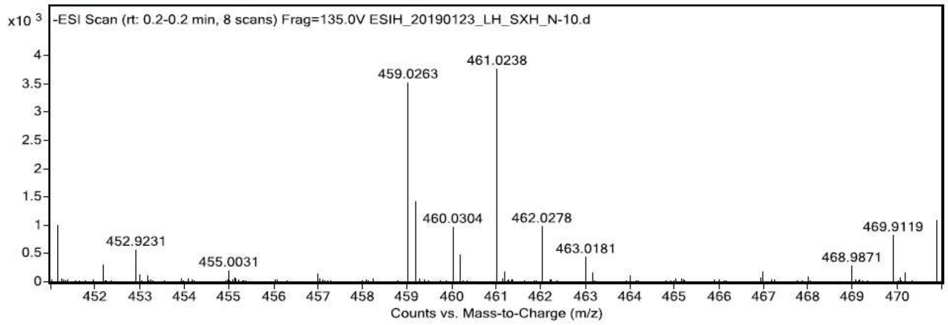

Formula Calculator Results

m/z	Calc m/z	Diff (mDa)	Diff (ppm)	Ion Formula	Ion
459.0263	459.0271	0.77	1.68	C22 H20 Br O4 S	(M-H)-

--- End Of Report ---

Ethyl 3-(dimethyl(oxo)-λ^6-sulfanylidene)-5-methyl-4-oxo-2-(4-(trifluoromethyl) ph-enyl)-3,4-dihydronaphthalene-1-carboxylate(**3ae**)

**Data Filename**	ESIH_20190125_LH_SXH_04.d	**Sample Name**	B6-SXH-2-4
**Sample Type**	Sample	**Position**	P1-B5
**Instrument Name**	Agilent G6520 Q-TOF	**Acq Method**	20160322_MS_ESIH_POS_1min.m
**Acquired Time**	1/25/2019 10:05:17	**IRM Calibration Status**	Success
**DA Method**	small molecular data analysis method.m	**Comment**	ESIH by ZZY

User Spectra

Fragmentor Voltage	Collision Energy	Ionization Mode
175	0	ESI

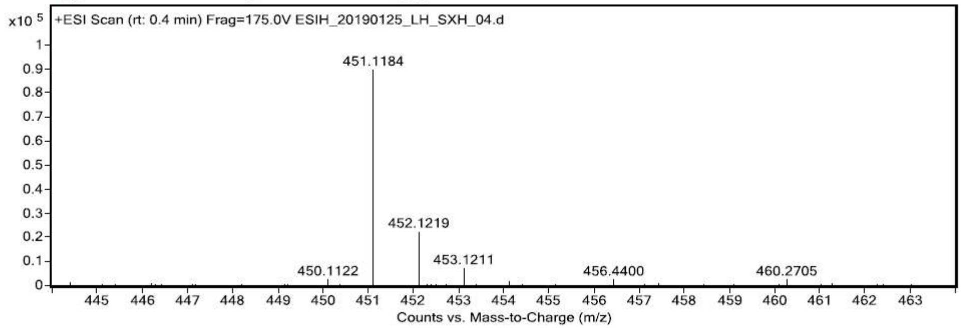

Formula Calculator Results

m/z	Calc m/z	Diff (mDa)	Diff (ppm)	Ion Formula	Ion
451.1184	451.1185	0.12	0.26	C23 H22 F3 O4 S	(M+H)+

--- End Of Report ---

Ethyl 3-(dimethyl(oxo)-λ^6-sulfanylidene)-2-(4-methoxyphenyl)-5-methyl-4-oxo-3,4 -dihydronaphthalene-1-carboxylate(**3af**)

**Data Filename**	ESIH_20190122_LH_SXH_08.d	**Sample Name**	B6-SXH-2-6
**Sample Type**	Sample	**Position**	P2-A8
**Instrument Name**	Agilent G6520 Q-TOF	**Acq Method**	20160322_MS_ESIH_POS_1min.m
**Acquired Time**	1/22/2019 9:50:38	**IRM Calibration Status**	Success
**DA Method**	small molecular data analysis method.m	**Comment**	ESIH by ZZY

User Spectra

Fragmentor Voltage	Collision Energy	Ionization Mode
125	0	ESI

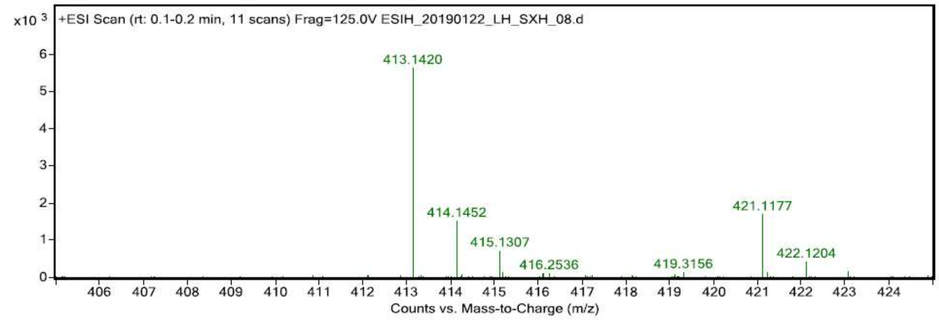

Formula Calculator Results

m/z	Calc m/z	Diff (mDa)	Diff (ppm)	Ion Formula	Ion
413.142	413.1417	-0.25	-0.59	C23 H25 O5 S	(M+H)+

--- End Of Report ---

Ethyl 3-(dimethyl(oxo)-λ^6-sulfanylidene)-2-(3-methoxyphenyl)-5-methyl-4-oxo-3,4-dihydronaphthalene-1-carboxylate (**3ag**)

**Data Filename**	ESIH_20190125_LH_SXH_02.d	**Sample Name**	B6-SXH-5-2
**Sample Type**	Sample	**Position**	P1-B3
**Instrument Name**	Agilent G6520 Q-TOF	**Acq Method**	20160322_MS_ESIH_POS_1min.m
**Acquired Time**	1/25/2019 10:01:35	**IRM Calibration Status**	Success
**DA Method**	small molecular data analysis method.m	**Comment**	ESIH by ZZY

User Spectra

Fragmentor Voltage	Collision Energy	Ionization Mode
175	0	ESI

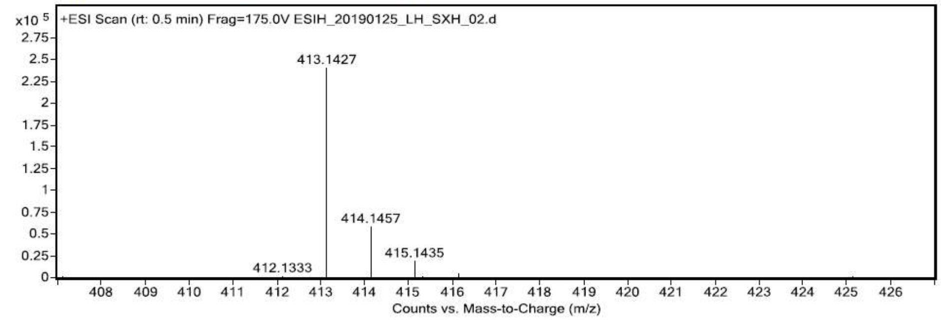

Formula Calculator Results

m/z	Calc m/z	Diff (mDa)	Diff (ppm)	Ion Formula	Ion
413.1427	413.1417	-1.02	-2.47	C23 H25 O5 S	(M+H)+

--- End Of Report ---

Ethyl 2-(3-bromophenyl)-3-(dimethyl(oxo)-λ^6-sulfanylidene)-5-methyl-4-oxo- 3,4-dihydronaphthalene-1-carboxylate (**3ah**)

**Data Filename**	ESIH_20190125_LH_SXH_03.d	**Sample Name**	B6-SXH-4-3
**Sample Type**	Sample	**Position**	P1-B4
**Instrument Name**	Agilent G6520 Q-TOF	**Acq Method**	20160322_MS_ESIH_POS_1min.m
**Acquired Time**	1/25/2019 10:03:27	**IRM Calibration Status**	Success
**DA Method**	small molecular data analysis method.m	**Comment**	ESIH by ZZY

User Spectra

Fragmentor Voltage	Collision Energy	Ionization Mode
175	0	ESI

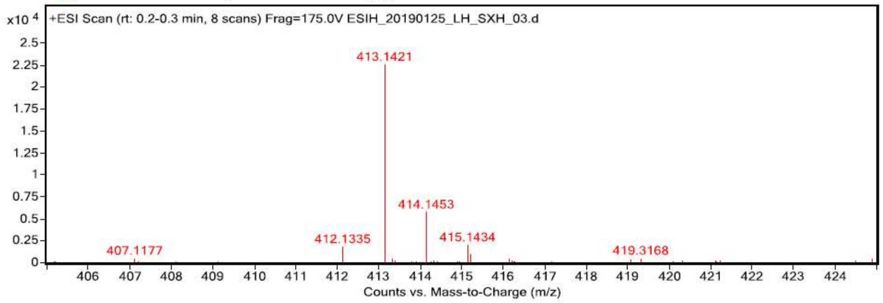

Formula Calculator Results

m/z	Calc m/z	Diff (mDa)	Diff (ppm)	Ion Formula	Ion
413.1421	413.1417	-0.4	-0.98	C23 H25 O5 S	(M+H)+

--- End Of Report ---

Ethyl 2-(2-chlorophenyl)-3-(dimethyl(oxo)-λ^6-sulfanylidene)-5-methyl-4-oxo- 3,4-dihydronaphthalene-1-carboxylate (**3ai**)

**Data Filename**	ESIH_20190228_LH_SXH_49.d	**Sample Name**	B6-SXH-7-3
**Sample Type**	Sample	**Position**	P1-F4
**Instrument Name**	Agilent G6520 Q-TOF	**Acq Method**	20160322_MS_ESIH_POS_1min.m
**Acquired Time**	2/28/2019 16:42:13	**IRM Calibration Status**	Success
**DA Method**	small molecular data analysis method.m	**Comment**	ESIH by ZZY

User Spectra

Fragmentor Voltage	Collision Energy	Ionization Mode
200	0	ESI

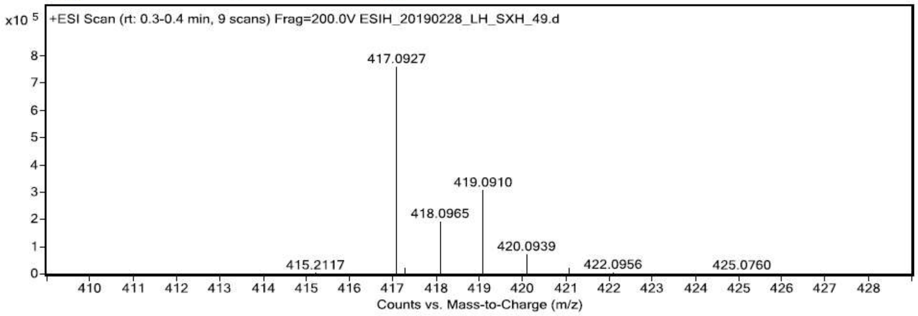

Formula Calculator Results

m/z	Calc m/z	Diff (mDa)	Diff (ppm)	Ion Formula	Ion
417.0927	417.0922	-0.51	-1.22	C22 H22 Cl O4 S	(M+H)+

--- End Of Report ---

Ethyl 3-(dimethyl(oxo)-λ^6-sulfanylidene)-2-(2-methoxyphenyl)-5-methyl-4-oxo-3,4-dihydronaphthalene-1-carboxylate(**3aj**)

**Data Filename**	ESIH_20190125_LH_SXH_03.d	**Sample Name**	B6-SXH-4-3
**Sample Type**	Sample	**Position**	P1-B4
**Instrument Name**	Agilent G6520 Q-TOF	**Acq Method**	20160322_MS_ESIH_POS_1min.m
**Acquired Time**	1/25/2019 10:03:27	**IRM Calibration Status**	Success
**DA Method**	small molecular data analysis method.m	**Comment**	ESIH by ZZY

User Spectra

Fragmentor Voltage	Collision Energy	Ionization Mode
175	0	ESI

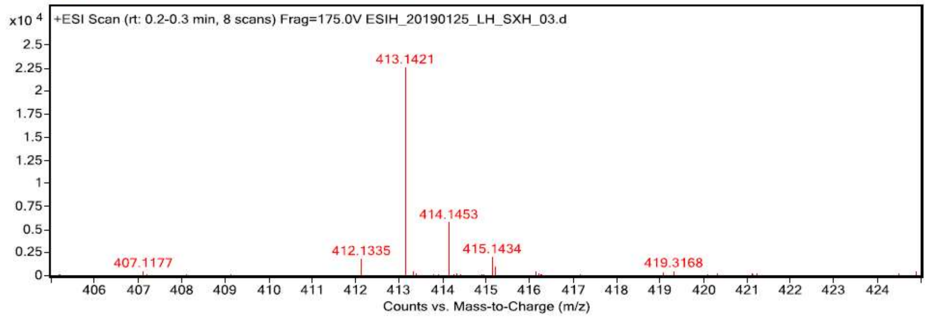

Formula Calculator Results

m/z	Calc m/z	Diff (mDa)	Diff (ppm)	Ion Formula	Ion
413.1421	413.1417	-0.4	-0.98	C23 H25 O5 S	(M+H)+

--- End Of Report ---

Ethyl 3-(dimethyl(oxo)-λ^6-sulfanylidene)-2,5-dimethyl-4-oxo-3,4-dihydronaphthalene-1-carboxylate(**3ak**)

**Data Filename**	ESIH_20190123_LH_SXH_06.d	**Sample Name**	B6-SXH-1-2
**Sample Type**	Sample	**Position**	P2-A4
**Instrument Name**	Agilent G6520 Q-TOF	**Acq Method**	20160322_MS_ESIH_POS_1min.m
**Acquired Time**	1/23/2019 14:59:53	**IRM Calibration Status**	Success
**DA Method**	small molecular data analysis method.m	**Comment**	ESIH by ZZY

User Spectra

Fragmentor Voltage	Collision Energy	Ionization Mode
125	0	ESI

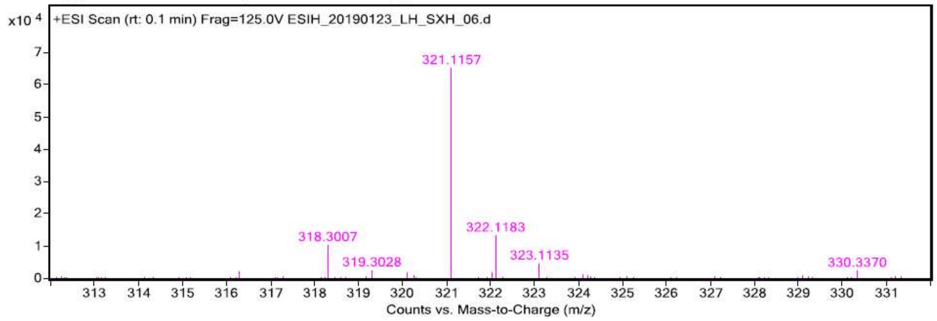

Formula Calculator Results

m/z	Calc m/z	Diff (mDa)	Diff (ppm)	Ion Formula	Ion
321.1157	321.1155	-0.23	-0.71	C17 H21 O4 S	(M+H)+

--- End Of Report ---

Ethyl 2-cyclopropyl-3-(dimethyl(oxo)-λ^6-sulfanylidene)-5-methyl-4-oxo-3,4-dihydronaphthalene-1-carboxylate(**3al**)

**Data Filename**	ESIH_20190123_LH_SXH_N-05.d	**Sample Name**	B6-SXH-1-3
**Sample Type**	Sample	**Position**	P2-A3
**Instrument Name**	Agilent G6520 Q-TOF	**Acq Method**	20160324_MS_ESIH_NEG_1min.m
**Acquired Time**	1/23/2019 16:10:34	**IRM Calibration Status**	Success
**DA Method**	small molecular data analysis method.m	**Comment**	ESIH by ZZY

User Spectra

Fragmentor Voltage	Collision Energy	Ionization Mode
135	0	ESI

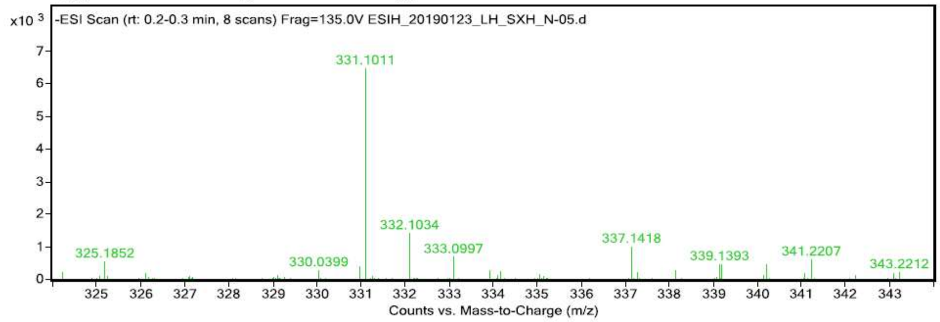

Formula Calculator Results

m/z	Calc m/z	Diff (mDa)	Diff (ppm)	Ion Formula	Ion
331.1011	331.101	-0.16	-0.49	C18 H19 O4 S	(M-H)-

--- End Of Report ---

Isopropyl 3-(dimethyl(oxo)-λ^6-sulfanylidene)-2,5-dimethyl-4-oxo-3,4-dihydronaphthalene-1-carboxylate(**3am**)

**Data Filename**	ESIH_20190125_LH_SXH_05.d	**Sample Name**	B6-SXH-2-5
**Sample Type**	Sample	**Position**	P1-B6
**Instrument Name**	Agilent G6520 Q-TOF	**Acq Method**	20160322_MS_ESIH_POS_1min.m
**Acquired Time**	1/25/2019 10:07:06	**IRM Calibration Status**	Success
**DA Method**	small molecular data analysis method.m	**Comment**	ESIH by ZZY

User Spectra

Fragmentor Voltage	Collision Energy	Ionization Mode
175	0	ESI

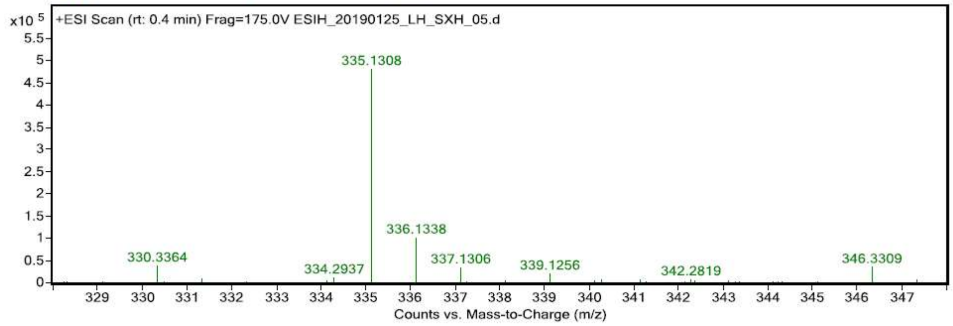

Formula Calculator Results

m/z	Calc m/z	Diff (mDa)	Diff (ppm)	Ion Formula	Ion
335.1308	335.1312	0.33	0.98	C18 H23 O4 S	(M+H)+

--- End Of Report ---

Tert-butyl 3-(dimethyl(oxo)-λ^6-sulfanylidene)-2,5-dimethyl-4-oxo-3,4-dihydronaphthalene-1-carboxylate(**3an**)

**Data Filename**	ESIH_20190306_LH_SXH_02.d	**Sample Name**	B6-SXH-0-6
**Sample Type**	Sample	**Position**	P1-C5
**Instrument Name**	Agilent G6520 Q-TOF	**Acq Method**	20160322_MS_ESIH_POS_1min.m
**Acquired Time**	3/6/2019 17:59:18	**IRM Calibration Status**	Success
**DA Method**	small molecular data analysis method.m	**Comment**	ESIH by ZZY

User Spectra

Fragmentor Voltage	Collision Energy	Ionization Mode
135	0	ESI

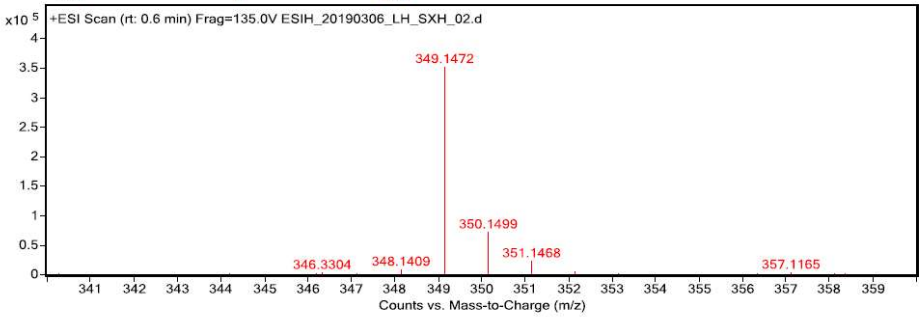

Formula Calculator Results

m/z	Calc m/z	Diff (mDa)	Diff (ppm)	Ion Formula	Ion
349.1472	349.1468	-0.36	-1.02	C19 H25 O4 S	(M+H)+

--- End Of Report ---

Ethyl 4-hydroxy-3-((4-methoxyphenyl)amino)-2,5-dimethyl-1-naphthoate (**5ak**)

**Data Filename**	ESIH_20190307_LH_SXH_N_04.d	**Sample Name**	B6-SXH-OMe
**Sample Type**	Sample	**Position**	P1-B2
**Instrument Name**	Agilent G6520 Q-TOF	**Acq Method**	20160324_MS_ESIH_NEG_1min.m
**Acquired Time**	3/7/2019 14:50:27	**IRM Calibration Status**	Success
**DA Method**	small molecular data analysis method.m	**Comment**	ESIH by ZZY

User Spectra

Fragmentor Voltage	Collision Energy	Ionization Mode
135	0	ESI

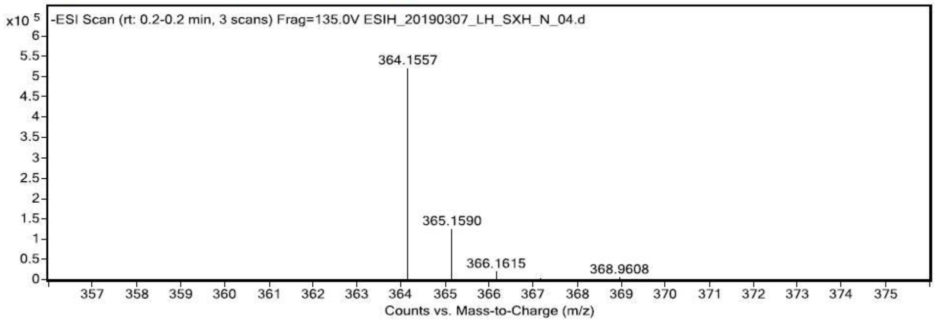

Formula Calculator Results

m/z	Calc m/z	Diff (mDa)	Diff (ppm)	Ion Formula	Ion
364.1557	364.1554	-0.31	-0.84	C22 H22 N O4	(M-H)-

--- End Of Report ---

Ethyl 4-hydroxy-2,5-dimethyl-3-(methylsulfinyl)-1-naphthoate (**6ak**)

**Data Filename**	ESIH_20190307_LH_SXH_N_05.d	**Sample Name**	B6-SXH-NaH
**Sample Type**	Sample	**Position**	P1-B3
**Instrument Name**	Agilent G6520 Q-TOF	**Acq Method**	20160324_MS_ESIH_NEG_1min.m
**Acquired Time**	3/7/2019 14:52:16	**IRM Calibration Status**	Success
**DA Method**	small molecular data analysis method.m	**Comment**	ESIH by ZZY

User Spectra

Fragmentor Voltage	Collision Energy	Ionization Mode
135	0	ESI

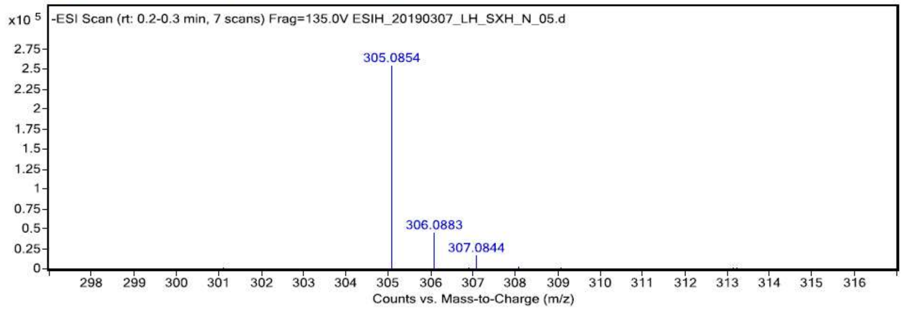

Formula Calculator Results

m/z	Calc m/z	Diff (mDa)	Diff (ppm)	Ion Formula	Ion
305.0854	305.0853	-0.07	-0.22	C16 H17 O4 S	(M-H)-

--- End Of Report ---
